# PUResNet: prediction of protein-ligand binding sites using deep residual neural network

**DOI:** 10.1186/s13321-021-00547-7

**Published:** 2021-09-08

**Authors:** Jeevan Kandel, Hilal Tayara, Kil To Chong

**Affiliations:** 1 Graduate School of Integrated Energy-AI, Jeonbuk National University, Jeonju, 54896 South Korea; 2 School of International Engineering and Science, Jeonbuk National University, Jeonju, 54896 South Korea; 3 Department of Electronics and Information Engineering, Jeonbuk National University, Jeonju, 54896 South Korea; 4 Advanced Electronics and Information Research Center, Jeonbuk National University, Jeonju, 54896 South Korea

**Keywords:** Ligand binding sites, Binding site prediction, Deep residual network, Convolutional neural network, Data cleaning

## Abstract

**Background:**

Predicting protein-ligand binding sites is a fundamental step in understanding the functional characteristics of proteins, which plays a vital role in elucidating different biological functions and is a crucial step in drug discovery. A protein exhibits its true nature after binding to its interacting molecule known as a ligand that binds only in the favorable binding site of the protein structure. Different computational methods exploiting the features of proteins have been developed to identify the binding sites in the protein structure, but none seems to provide promising results, and therefore, further investigation is required.

**Results:**

In this study, we present a deep learning model PUResNet and a novel data cleaning process based on structural similarity for predicting protein-ligand binding sites. From the whole scPDB (an annotated database of druggable binding sites extracted from the Protein DataBank) database, 5020 protein structures were selected to address this problem, which were used to train PUResNet. With this, we achieved better and justifiable performance than the existing methods while evaluating two independent sets using distance, volume and proportion metrics.

**Supplementary Information:**

The online version contains supplementary material available at 10.1186/s13321-021-00547-7.

## Introduction

In living organisms, all biological processes involve proteins that are dynamic molecules with functions almost invariably dependent on the interactions with other molecules, which are affected in physiologically important ways through subtle, or striking changes in the protein conformation [1]. Such interactions occur in a specific site of a protein known as binding site, and any interacting molecule, ion, or protein is known as ligand. Elucidating the characteristics and function of a protein depends solely on its interaction with the ligand at a suitable binding site. The prediction of such binding sites is the first step towards understanding the functional properties of the proteins leading to drug discovery.

In recent years, numerous methods have been proposed to identify the potential druggable binding sites. Fpocket [2] is a geometry-based method, which is based on Voronoi tessellation and alpha spheres. The alpha sphere is a sphere that contacts four atoms on its boundary and contains no internal atom, which was introduced by Liang and Edelsbrunner [3]. LIGSITE [4] and POCKET [5] are based on a regular Cartesian grid, where if an area of solvent-accessible grid points are enclosed on both sides by the protein atoms, then it has a higher chance of being located in a pocket or cavity. EASYMIFs and SITEHOUND [6] are energy-based methods, where the molecular interaction fields (MIFs) are used to identify the probable binding sites through filtering and clustering. ConCavity [7] is a geometry-based method that combines evolutionary sequence conservation. COACH [8] is a consensus method based on a template in which the pocket is predicted by using a support vector machine (SVM). Other than the traditional methods for predicting the binding site, which are based on geometry, energy, evolutionary, consensus, and template, machine learning and deep learning methods have successfully emerged in recent years.

P2Rank [9] is a machine learning-based method for predicting ligand binding sites in the protein structures that imposes the random forest algorithm, where the 1D feature vector represents 35 numerical features and is trained on CHEN11 [10] dataset. DeepSite [11], kalasanty [12], DeepSurf [13] and DeepPocket [14] are deep learning approaches, which are based on 3D convolutional neural networks. In DeepSite and kalasanty methods, the protein structure is treated as a 3D image discretized into a grid of 1 × 1 × 1 Å^3^ sized voxel. DeepSite uses 16 × 16 × 16 voxels, whereas kalasanty uses 36 × 36 × 36 voxels to represent a protein structure. DeepSurf is a surface based learning approach where a new representation of the 3D protein surface is introduced, based on local voxel grids centered at sample points of the surface and uses 16 × 16 × 16 voxels. DeepPocket is a multi-step approach to get the final pocket location where first Fpocket is used to get the pockets and later classified whether they are binding site or not. All these methods shows promising results and uses scPDB [15] dataset. To improve result, filtering of scPDB dataset based on structural similarity is required which is not done by any mentioned deep learning methods. Although, DeepSite employs sequence similarity method to eliminate similar protein structure but we are more focused on structural similarity.

Our work is focused on improving the training data, so that our deep learning model can generalize more and provide better predictions. Therefore, we developed an independent training dataset, which is a subset of scPDB [15], a publicly available dataset released in 2017, containing 16034 entries, 4782 proteins, and 6326 ligands. Among 16034 protein structures present in scPDB, we selected 5020 structures. First, each of the protein structures from scPDB were grouped according to the UniProt ID [16], and then the Tanimoto coefficient [17] was calculated. Second, longest sequenced protein structure was selected from each UniProt ID cluster according to the Tanimoto coefficient (if Tanimoto coefficient $\ge$ 80%, then it is regarded as a similar structure [17]). Finally, manual inspection was performed using PYMOL [18] and 5020 protein structures were selected out of 16034.

In this study, ResNet [19] architecture is used as a backbone for our model (PUResNet). ResNet is one of the popular deep learning architecture due to residual learning and identity mapping by shortcuts [19]. PUResNet comprises two blocks, encoder and decoder, where there is a skip connection between encoder and decoder as well as within the layers of encoder and decoder. Skip connections are used to address the vanishing gradient problem, which is the most common problem in training deep neural networks [20].

Protein structure is treated as a 3D image of the shape (36 × 36 × 36 × 18) which is input to PUResNet, and the output is the same as the input shape with a single channel (i.e., 36 × 36 × 36 × 1), where each voxel (point in 3D space) in the output has a probability that whether or not the voxel belongs to the cavity. Later, these predictions can be saved as mol2 files, which can be later visualized using the molecular modeling software (PYMOL).

## Materials

In this study, new training dataset is developed, which is a subset of scPDB. scPDB dataset consists of protein structures belonging to 2050 different protein families [21]. The family Pkinase contains the highest number of protein structures (1486) whereas 555 protein families contain only a single structure. As an independent validation dataset, we selected COACH420 [22] test dataset, which consists of 420 protein structure with known ligands, and among them 122 protein structures were removed since they were present in our dataset. Finally, 298 protein structure with ligand were selected. Additionally, BU48 [23] dataset consisting of 48 pairs of bounded and unbounded protein structure, among which 31 pair were selected as an independent dataset, after removing protein structure contained in our training set.

### Data cleaning

**Fig. 1:**
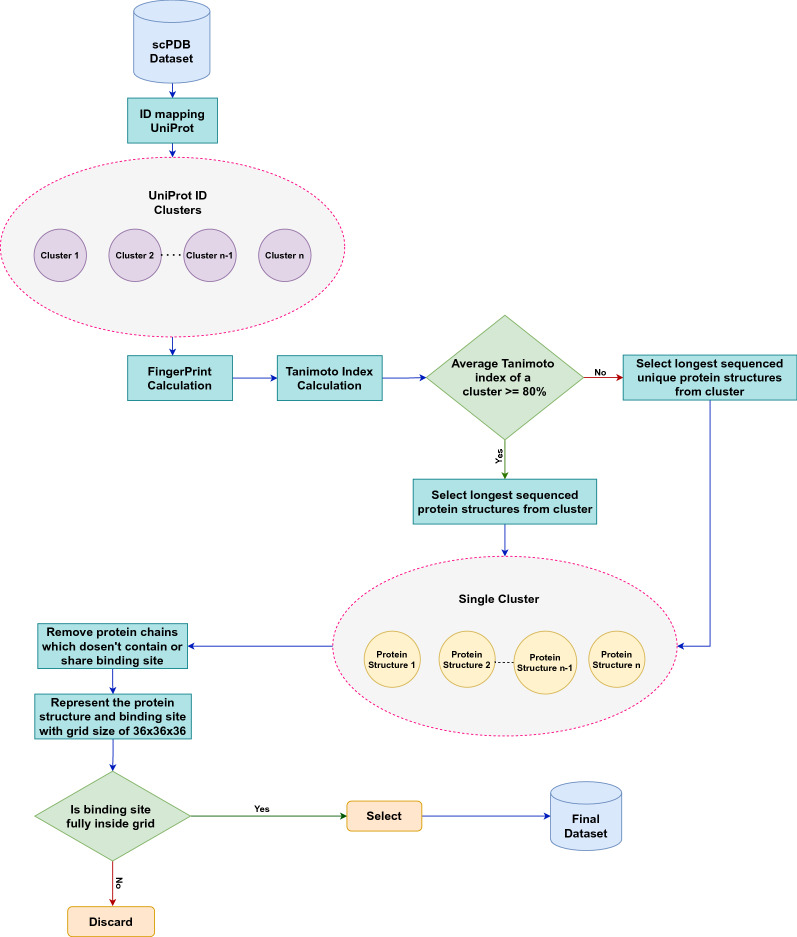
Flow diagram of data cleaning process

304 protein structures that were erroneous while loading using openbabel [24, 25] were removed from scPDB dataset. Then, we followed the process of data cleaning, as depicted in Fig. 1. First, the grouping of protein structure according to the UniProtID was conducted using the Retrieve/ID mapping tool available online (https://www.uniprot.org/uploadlists/). A total of 5462 clusters of UniPort ID were obtained, of which 2964 contained a single protein structure and 2498 contained multiple protein structures. The cluster of UniPort ID (P00388), which had 19 protein structures, was the largest of all. Second, each protein structure in the cluster fingerprint was determined, where we used a substructure-based fingerprint calculation molecular access system (MACCS) [26], and then the Tanimoto index was calculated within each cluster.

**Fig. 2:**
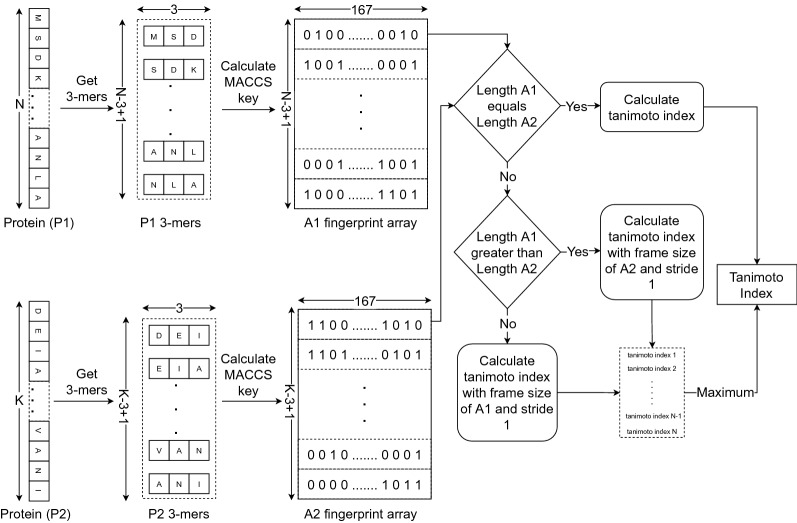
Flow diagram showing calculation of Tanimoto index

For calculating Tanimoto index as shown in Fig. 2, protein structure having N amino acids, we obtained N-3+1 number of 3-mers (consecutive amino acid substrings of length three within a protein sequence obtained using frame size of three and stride of one), where each 3-mers is represented as a single molecule using openbabel and 167-bit MACCS key was obtained. Then, fingerprint for the protein structure is an array of size (N-3+1,167), where each row contains MACCS key for corresponding 3-mers. Let, A1 and A2 be fingerprint arrays for two protein structures. If the length of A1 and A2 is equal then the Tanimoto index is calculated between A1 and A2. Else if the length of A1 is greater than A2 then the Tanimoto index is calculated with the frame size of A2 with stride 1 and the maximum Tanimoto index is taken from calculated ones. Else Tanimoto index is calculated with the frame size of A1 with stride 1 and maximum Tanimoto index is taken from obtained values.

On an average, for each cluster having multiple protein structure, the Tanimoto index was found to be 80%, and therefore, we decided to select the longest sequenced protein structure from each cluster because of high similarity between the protein structure in the cluster [17]. The total number of selected protein structures was 5462 corresponding to unique UniPort IDs as a single cluster. For each protein structure in a single cluster, manual inspection was performed using PYMOL. Protein structures along with their binding sites were loaded in PYMOL, the chain with the binding site was retained, and the others were removed. As we treat the protein structure as a 3D image and classify each voxel as a binding site or not. Data imbalance occurs when data points are not equally distributed among classes. In our case, the number of voxels not belonging to the binding site is very high which makes our problem to be highly imbalanced. In a protein structure represented in 3D image, the ratio of voxels belonging to binding site to the voxels not belonging to binding site is about 0.001. Due to high data imbalance, the removal of chains without a binding site is necessary to address this problem. Although we cannot fully eradicate this problem, this step provides little leverage to the model. After that, the distance between the binding site coordinates to the center of the protein structure was calculated; if the distance between any coordinate of the binding site and the protein structure center is greater than 70 Å, then it is removed because such a binding site cannot be represented in voxels, and this will lead to training data without a binding site or a portion of the binding site. Finally, 5020 protein structures were selected for training, corresponding to 5020 Uniport ID and 1243 protein families, among which the Pkinase family contained 186 protein structures, and was largest of all.

We split our data into four folds by addressing the problem of data leakage during validation, based on the protein family, all the structures belonging to one family were kept in the same set of each fold (either on training or validation set). In each fold, the training set consisted of 3765 protein structures, whereas the validation set had 1255. We used k-fold [27, 28] training to tune the hyperparameters and validate PUResNet. After selecting the optimal parameters, the model was trained on the entire dataset for better performance.

### Data representation

Here, the protein structure was treated as a 3D image of size 36 × 36 × 36 × 18, where a 3D cube of size 36 × 36 × 36 is placed at the center of a protein with 70 Å distance in each direction, and was described based on nine atomic features [29], such as hybridization, heavy atoms, heteroatoms, hydrophobic, aromatic, partial charge, acceptor, donor, and ring. Plotting of each atomic feature used in the study of the protein structure (1A80) is provided in Additional file 1. Finally, one protein structure was represented with 3D voxels of size 36 × 36 × 36 × 18.

To treat it as a binary segmentation problem where input size is 36 × 36 × 36 × 18 and output size is 36 × 36 × 36 × 1, each binding site was represented using same sized 3D voxels (36 × 36 × 36 × 1) placed at the protein center, and for each voxel, if the binding site was present, then the assigned value was 1 or else 0.

## Model

PUResNet is derived from the concept of U-Net [30] and ResNet. U-Net was originally developed for the segmentation of biomedical images, which are composed of convolutional and max-pooling layers in the encoder side and convolutional and up sampling layers in the decoder side. Moreover, there is a skip connection between the encoder and decoder blocks. Here, we propose a variant of U-Net that consists of three basic blocks (convolution, identity, and up-sampling blocks), as depicted in Additional file 2: Figure 1S, 2S and 3S which are based on the concept of ResNet. Unlike the 2D segmentation problem, which uses 2D convolution, we used 3D convolution to address our problem.

**Fig. 3:**
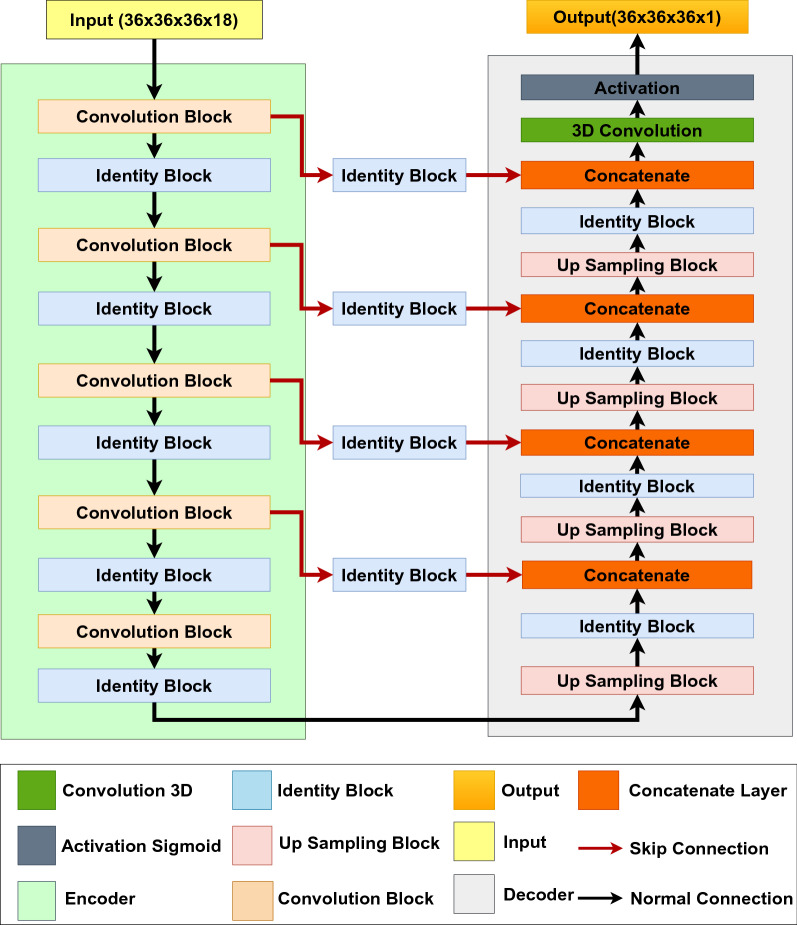
Model PUResNet architecture showing both encoder and decoder block with skip connections

PUResNet is divided into two blocks, an encoder and a decoder, as depicted in Fig. 3, where the encoder is composed of a convolution block and an identity block (which has convolution layers as shown in Additional file 2: Figure 2S ), and the decoder is composed of an up-sampling block and an identity block. Instead of directly passing the skip connection to the decoder block like U-net, we first pass it through the identity block and then to the decoder, which leads to use of identity operation [19] in the skip connection. The idea behind designing this model is to address the vanishing gradient problem. To validate, whether skip connection helps in eliminating the vanishing gradient problem, we visualized the training process of two variants of PUResNet, one with skip connection and one without. As shown in Additional file 4: Figure 9S we can see that the accuracy of the PUResNet (learning rate = 10^−5^, kernel regularizer as L2 with value of 10^−3^, batch size of 5) without skip connections is almost constant which implies as the model is deep, gradients are either exploding or vanishing (shown in Additional file 4 Figure 10S). To counter this problem, skip connection inspired from ResNet architecture, are added in PUResNet which drastically changes the performance of the model as shown in Additional file 4: Figure 11S. One of the benefits of using skip connection is to eliminate exploding or vanishing gradients(as shown in Additional file 4: Figure 12S) in deep neural networks [20]. Here in PUResNet, there are 12 layers in the convolution block, 10 layers in the identity block, and 14 layers in the up sampling block. Altogether, there are 5 convolution blocks, 13 identity blocks, and 4 up sampling blocks. Number of filters used in each block is provided in Additional file 2: Table S1 and input/output size of each block is shown in Additional file 2: Figure S4. In total, there are 252 layers in PUResNet with 13,840,903 trainable parameters and 16,992 non-trainable parameters. Although PUResNet is deep but has a smaller number of parameters than kalasanty, which has 23 million parameters. A detailed explanation of this model is provided in Additional file 2.

### Model optimization

Our approach to optimize the hyperparameters was conducted through K-fold training, and we implemented the hit and trial approach using a heuristic method for optimizing the model. To select the value of K during the K-fold training, we assessed the validation and training curves for different values of K and found that K = 4 exhibits a smoother validation and training curve for our dataset. Hyperparameter optimization was conducted through selecting two sets of hyperparameters in such a way that the difference in values was high. K-fold training was conducted using the two sets of hyperparameters and determined which set had good performance, and then, the average value of the two sets was computed. After that, K-fold training was performed using individual values keeping others the same, and the results were obtained. If the performance was better than the previous result, then those values were selected and otherwise discarded. Further, we selected the top two results from K-fold training, which was conducted recursively until optimal parameters were obtained. Here, while selecting the optimal parameter, we considered every data point as the validation data using cross-validation so that our parameters were not biased towards a certain protein structure. Finally, after obtaining a set of optimal hyperparameters, we conducted K-fold cross-validation using K = 4, and the results were obtained. Final training was performed on the entire dataset with the obtained optimal parameters (learning rate = 10^−4^, kernel regularizer as L2 with value of 10^−4^, batch size of 5, number of trainable parameters 13,840,903, and others as default values as in keras [31]). Dice loss and binary crossentropy are widely used loss functions in the case of binary segmentation problems. To find out the performance of these loss functions, we carried out 4 fold experiment. As shown in Additional file 4: Figure 1S to 8S, we can observe that the dice loss has better performance than binary crossentropy (learning rate = 30^−7^, kernel regularizer as L2 with value of 10^−5^, batch size of 5). As expected, dice loss performs better in the case of highly a imbalanced dataset [32]. Therefore, we selected dice loss as our loss function.

## Results

Distance center center (DCC) and discretized volume overlap (DVO) are the matrices used to evaluate model in different studies [9, 11, 12]. In this study, we propose new metrics, the Proportion of Ligand Inside (PLI) for the accountability of ligands and predicted binding sites.

- Distance center center (DCC)
  It is distance between center of predicted binding site to the center of actual binding site or ligand. If the distance is $\le$ 4 Å, then it is determined to be correctly predicted site, which is used to measure the success rate of the model and defined as follows:
  1 $$\begin{aligned} Success Rate = \frac{Number \, of \, sites \, having \, DCC\le 4A^o }{Total \, number \, of \, sites} \end{aligned}$$
- Discretized volume overlap (DVO)
  DCC metric does not consider the volume and shape of the predicted and actual binding sites or ligands. Therefore, DVO, which provides insight into the volume and shape, is the ratio between the volumetric intersection between the predicted(V_pbs_) and actual binding site(V_abs_) to their union. For predicted binding sites having DCC $\le$ 4 Å, DVO was calculated as follows:
  2 $$\begin{aligned} DVO=\frac{V_{pbs} \, \cap \, V_{abs}}{V_{pbs} \, \cup \, V_{abs}} \end{aligned}$$
- Proportion of Ligand Inside (PLI)
  In case of ligands using DVO metrics to find overlap does not provide a comprehensive idea of the overlap, the binding sites are usually larger than the ligand. The DVO metric is similar to a shape analysis between the two binding sites, but in case of ligands and binding sites, it is not appropriate. Therefore, we developed a new matrix to determine the proportion of ligand (V_L_) resides inside binding site(V_pbs_). For predicted binding sites with a DCC less than or equal to 4 Å, PLI was calculate as follows:
  3 $$\begin{aligned} PLI=\frac{V_{L} \, \cap \, V_{pbs}}{V_{L}} \end{aligned}$$

DCC was calculated by taking the center of the predicted and actual binding sites, and DVO by representing both the predicted and actual binding sites (for PLI Ligand) in a 3D grid of size 36x36x36. To calculate the F1 score, we considered a predicted binding site with a DCC less than or equal to 4 Å as true positive (TP), greater than 4 Å as false positive (FP) and no prediction as false negative (FN). In this problem, there is no true negative since every protein structure has a binding site.

### K-Fold cross validation result

**Fig. 4:**
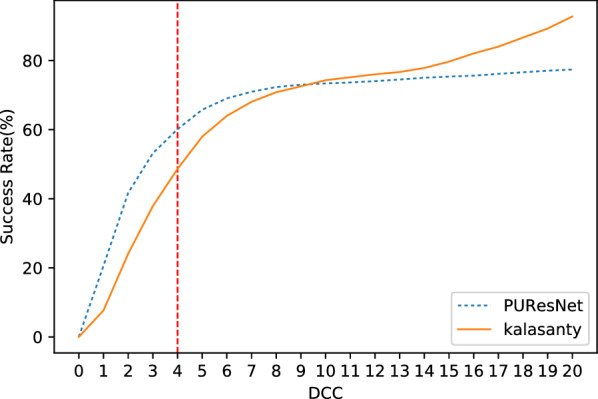
Success rate plot for different DCC values combining all fold (kalasanty vs PUResNet)

**Fig. 5:**
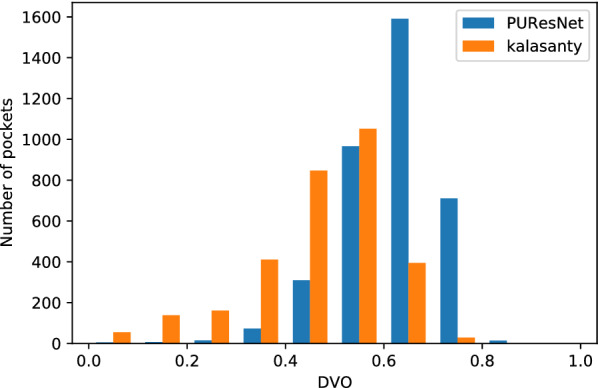
Histogram of DVO values combining all folds (kalasanty vs PUResNet)

We conducted our experiment in 4 folds, where the entire dataset was divided into four parts, leaving one part as the validation set and the other as the training set; and thus, we obtained four different models. Each model was compared with the kalasanty, which we trained on each fold-keeping with obtained optimal (using our optimization technique) parameters (learning rate = 10^−3^, kernel regularizer as L2 with value of 10^−4^, batch size of 5 and others as default values as in keras [31]). Combining all folds, out of 5020 protein structures, kalasanty did not identify any binding site for 76 protein structures (i.e., 6% of total protein structure) and PUResNet did not identify any binding site for 122 protein structures (i.e., 10% of total protein structure). For 64% of protein structures, kalasanty returned a single binding site, whereas PUResNet returned a single binding site for 93% of protein structures. Here, we were able to achieve an average F1 score of 0.83, which is 0.22 more than that of kalasanty, as shown in Table 1. PUResNet achieved a 61% success rate, whereas kalasanty achieved 51%, as shown in Fig. 4. Average DVO (shown in Fig. 5) of kalasanty is 0.46, whereas that of PUResNet is 0.61 combining results of all fold. Therefore, PUResNet can predict the binding sites more precisely and accurately compared to kalasanty. More detailed results for each fold are provided in Additional file 3.

**Table 1:** KFold validation result

Fold	Model	TP	FP	FN	F1 score
	kalasanty	741	1041	37	0.58
1st	PUResNet	916	349	42	0.82
	kalasanty	781	894	18	0.63
2nd	PUResNet	903	384	29	0.81
	kalasanty	815	983	7	0.62
3rd	PUResNet	960	293	19	0.86
	kalasanty	751	1049	14	0.59
4th	PUResNet	913	365	32	0.82
				kalasanty	0.61
Average				PUResNet	0.83

True Positive (TP), False Positive (FP), False Negative (FN) and F1 score obtained in different fold by kalasanty and PUResNet.

### Independent set result

**Fig. 6:**
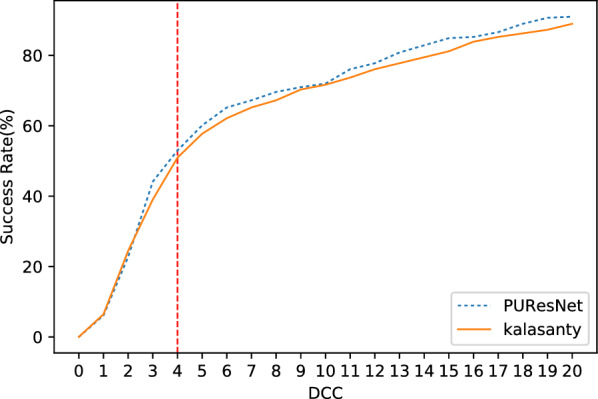
Success rate plot for different DCC values in Coach420 dataset (PUResNet vs kalasanty)

**Fig. 7:**
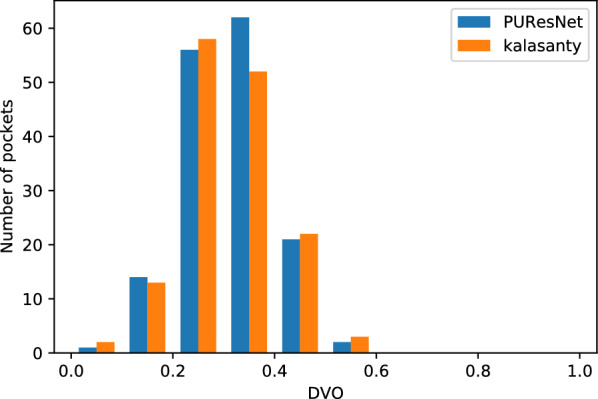
Histogram of DVO values for protein structure having DCC $\le$ 4 Å in Coach420

**Fig. 8:**
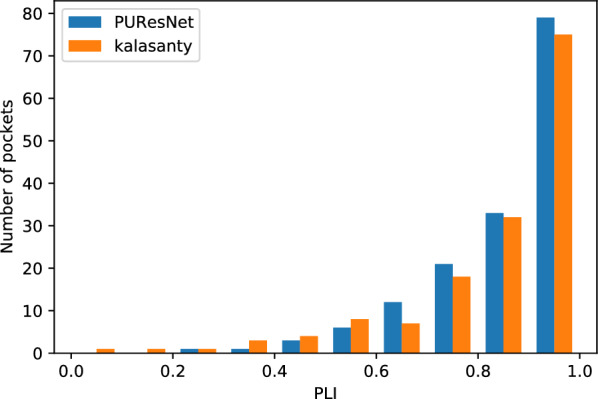
Histogram of PLI values for protein structure having DCC $\le$ 4 Å in Coach420

The evaluation was conducted using the Coach420 and BU48 datasets individually to determine the performance of PUResNet and kalasanty. In the Coach420 dataset, kalasanty did not provide any output for 26 protein structures (i.e., 8% of total protein structure), whereas PUResNet did not provide any output for 19 protein structures (i.e., 6% of total protein structure), as shown in Table  2. PUResNet has a success rate of 53%, average DVO of 0.32, and average PLI of 0.87, whereas kalasanty has a success rate of 51%, average DVO of 0.30, and PLI of 0.82, as shown in Table  2 and Figs. 6, 7,8. Kalasanty has an F1 score of 0.64, whereas PUResNet has an F1 score of 0.66, as shown in Table 2.

**Fig. 9:**
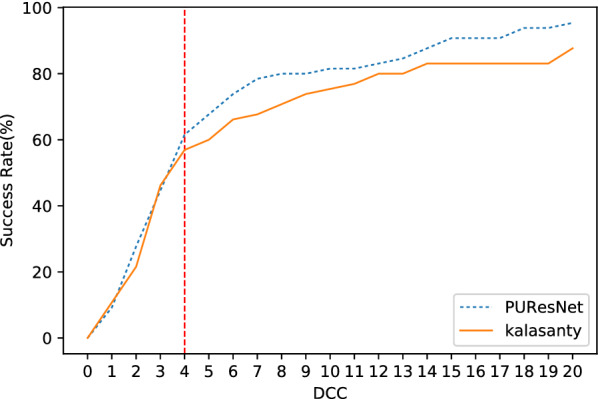
Success rate plot for different DCC values in BU48 dataset (PUResNet vs kalasanty)

**Fig. 10:**
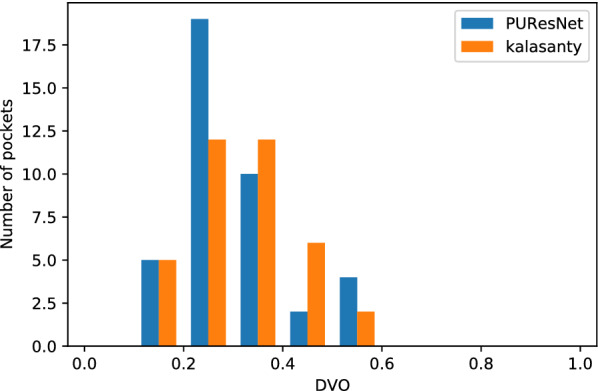
Histogram of DVO values for protein structure having DCC $\le$ 4 Å in BU48

**Fig. 11:**
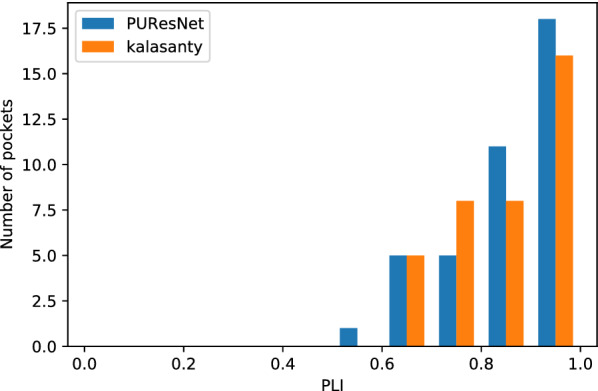
Histogram of PLI values for protein structure having DCC $\le$ 4 Å in BU48

In case of BU48 dataset, PUResNet did not provide any output for 3 protein structures (i.e., 4% of total protein structure), whereas kalasanty did not provide any output for 7 protein structures (i.e., 11% of total protein structure), as shown in Table  2. PUResNet has a success rate, average DVO, and average PLI of 62%, 0.31, and 0.89, respectively, whereas kalasanty has 57%, 0.30, and 0.82, respectively, as shown in Table 2 and Figs. 9, 10 and 11. F1 score was calculated to be 0.71 for both models, as shown in Table 2. Clearly, in both independent dataset PUResNet has better performance than kalasanty.

**Table 2:** Independent test results

Dataset	Model	TP	FP	FN	F1 score	Success rate (%)	Avg DVO	Avg PLI
	kalasanty	150	142	26	0.64	51	0.30	0.82
Coach 420	PUResNet	156	141	19	0.66	53	0.32	0.87
	kalasanty	37	23	7	0.71	57	0.30	0.82
BU48	PUResNet	40	30	3	0.71	62	0.31	0.89

Comparison between kalasanty and PUResNet in terms of True Positive (TP), False Positive (FP), False Negative (FN), F1 score, Success Rate, Average (Avg) DVO and Average (Avg) PLI obtained in independent test

## Discussion

**Fig. 12:**
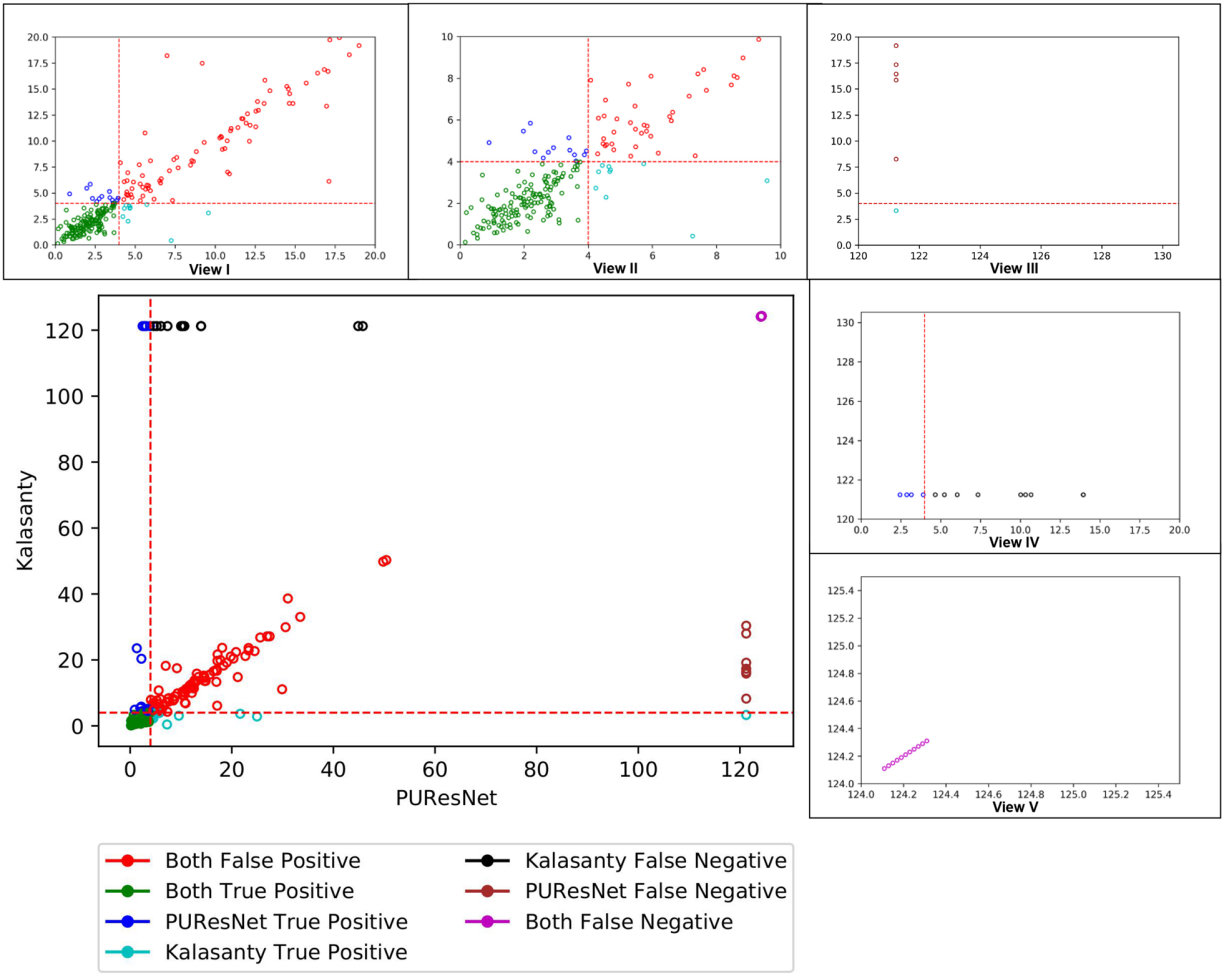
Scatter plot showing DCC values of Coach420 dataset predicted by kalasanty and PUResNet with different views(I-V), View I showing DCC values $\le$ 20 Å from PUResNet and kalasanty, View II showing DCC values $\le$ 10 Å from PUResNet and kalasanty, View III showing DCC values $\ge$ 120 Å from PUResNet and $\le$ 20 Å from kalasanty, View IV showing DCC values $\ge$ 120 Å from kalasanty and $\le$ 20Å from PUResNet and View V showing DCC values $\ge$ 124 Å from kalasanty and PUResNet

**Fig. 13:**
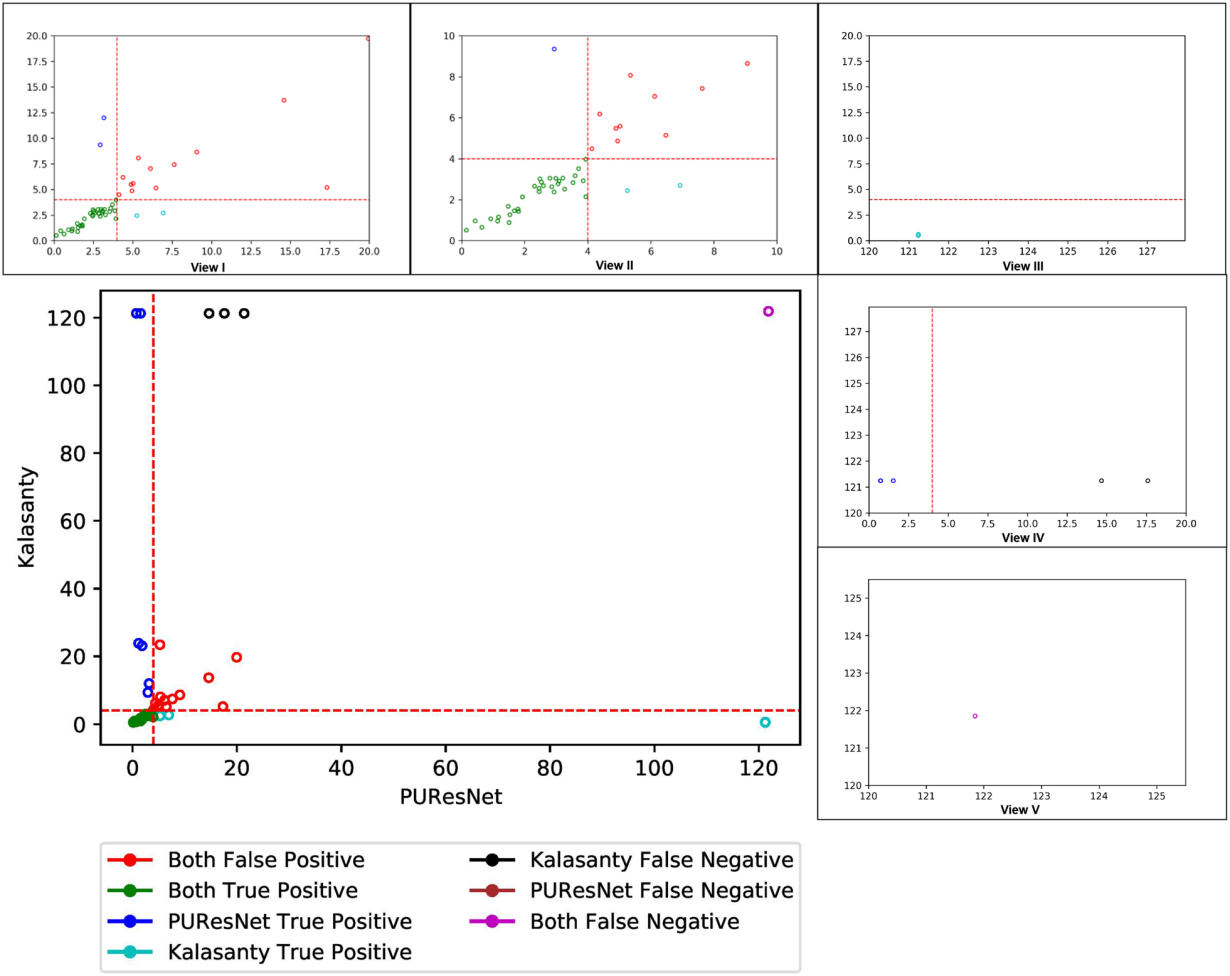
Scatter plot showing DCC values of BU48 dataset predicted by kalasanty and PUResNet with different views (I–V), View I showing DCC values $\le$ 20 Å from PUResNet and kalasanty, View II showing DCC values $\le$ 10 Å from PUResNet and kalasanty, View III showing DCC values $\ge$ 120 Å from PUResNet and $\le$ 20 Å from kalasanty, View IV showing DCC values $\ge$ 120 Å from kalasanty and $\le$ 20 Å from PUResNet and View V showing DCC values $\ge$ 120 Å from kalasanty and PUResNet

To better understand the performance of PUResNet, we further investigated each individual prediction made using PUResNet and kalasanty in the Coach420 and BU48 datasets. Figures 12 and 13 show the DCC values for individual protein structures predicted by kalasanty and PUResNet present in the Coach 420 and BU48 datasets, respectively. DCC values greater than or equal to 121.24 Å corresponds to the protein structures for which not even a single binding site was identified.

Out of 298 protein structures in the Coach420 dataset, both the models correctly predicted 137 protein structures, incorrectly predicted 100 protein structures, and for 11 protein structures, no site was predicted, as shown in Fig. 12 View I, II and V. Excluding the common predictions, kalasanty specifically provided output for eight protein structures (Fig. 12 View III) for which PUResNet did not provide any output. Among them, one protein structure was correctly predicted by kalasanty. Moreover, PUResNet predicted 14 protein structures (Fig. 12 View IV) for which no prediction was provided by kalasanty, and among them, four were correctly predicted. Additionally, 15 protein structures were correctly predicted by PUResNet, which were falsely predicted by kalasanty, whereas 12 protein structures were correctly predicted by kalasanty, which were falsely predicted by PUResNet. The average DVO for the common correctly predicted structures by both the models was 0.31, whereas the average PLI for PUResNet was 0.87, and that of kalasanty was 0.85.

Similarly, for BU48 dataset containing 62 protein structures (31 pairs of bound and unbound structures), 33 structures were correctly predicted, 14 were incorrectly predicted, and for one structure, no site was predicted, which was common among both the models, as shown in Fig. 13 View I, II and V. Excluding common predictions, 7 protein structures were correctly predicted by PUResNet; and among them, for two protein structures, kalasanty did not predict any site (Fig. 13 View IV), whereas 4 structures that were correctly predicted by kalasanty, among them for one PUResNet did not returned any site (Fig. 13 View III). For the three protein structures that were falsely predicted by PUResNet, kalasanty did not return any site. The average DVO for common correct prediction by each model is 0.28, whereas the average PLI of kalasanty and PUResNet is 0.86 and 0.87, respectively.

**Fig. 14:**
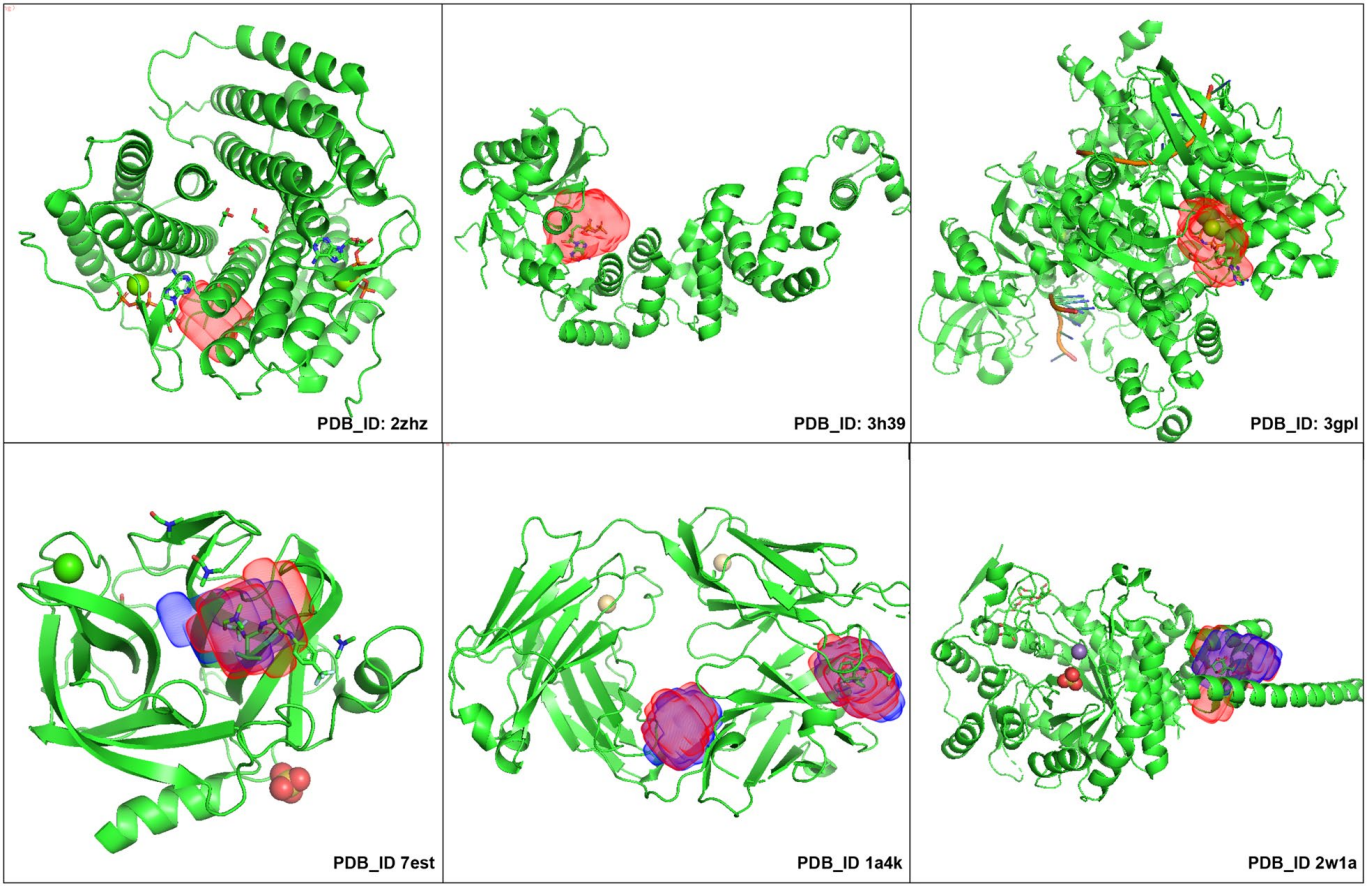
Protein strucutre ( 2zhz, 3h39, 3gpl, 7est, 2w1a, 1a4k) from Coach420, showing predicted binding site by kalasanty(Blue region) and PUResNet (Red Region)

In the Coach420 dataset, protein structures 2zhz, 3h39, and 3gpl (shown in Fig. 14) have binding sites for the ATP(ADENOSINE-5’-TRIPHOSPHATE) ligand, which was completely missed by kalasanty, although there were 401 protein structures having ATP binding site in the scPDB dataset, whereas PUResNet predicted the binding site for all three structures, and among them, correct prediction was made for 3h39 and 3gpl (shown in Fig. 14). Protein Structure’s (7est, 2w1a, 1a4k as shown in Fig. 14) binding site in both the model’s prediction are different in shape and size.

**Fig. 15:**
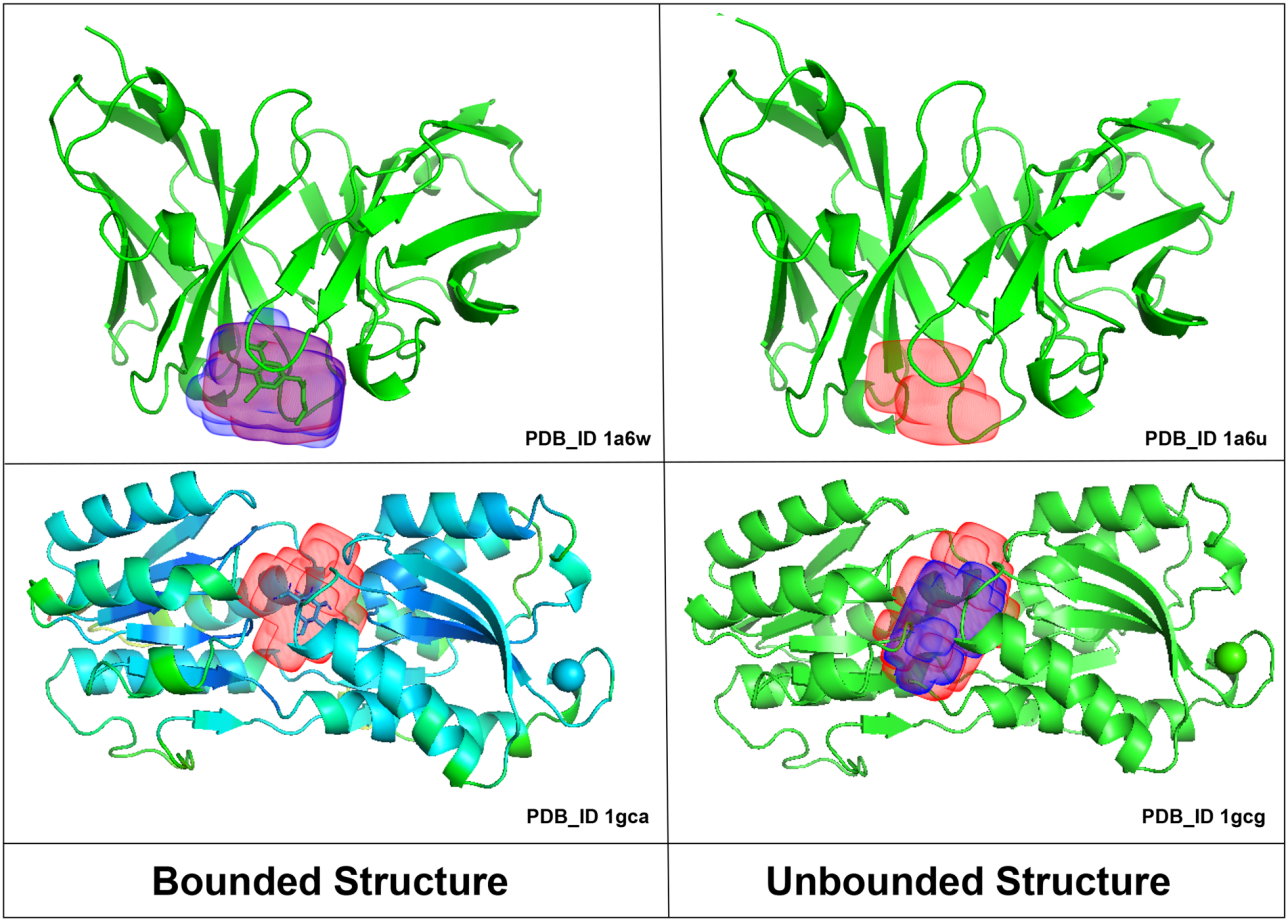
Bound and Unbound pair ((1a6u,1a6w), (1gcg,1gca)), showing predicted binding site by kalasanty(Blue region) and PUResNet (Red Region)

In BU48 dataset consisting of 31 pairs of bound and unbound structures, kalasanty completely missed to predict the unbound structures (1a6u,1krn,2ctv,2pk4 and 6ins) and bound structures (5cna and 1gca); however, PUResNet predicted all unbounded structure and did not predict bound structures (1rob, 6rsa and 5cna). For pairs ((1a6u, 1a6w), and (1gcg, 1gca) as shown in Fig. 15), PUResNet correctly predicted the binding sites, whereas kalasanty correctly predicted for 1gcg and 1a6w only. The binding site predicted by PUResNet for bound (1gca, 1a6w) and unbound (1a6u, 1gcg) structures has different shapes and sizes as shown in Fig. 15. Interestingly, for the pair (5cna, 2ctv), PUResNet was able to correctly predict the unbound 2ctv but kalasanty completely missed it. Therefore, we can conclude that the prediction made by PUResNet is distinct and better than that made by kalasanty.

To validate, whether our data cleaning process improves the performance of PUResNet, we performed an experiment in which PUResNet is trained on the original scPDB dataset. As shown in Additional file 4: Figure 11S,12S,13S,14S,15S, and 16S, we found out that in BU48 dataset as well as in Coach420 dataset, PUResNet trained on filtered dataset has better performance than PUResNet (learning rate = 30^−5^, kernel regularizer as L2 with value of 10^−4^, batch size of 10) trained on the original dataset.

## Conclusion

We introduced a new deep learning model, PUResNet, to predict the ligand-binding sites on protein structures trained on a newly formed dataset, which is a subset of scPDB. We compared our results with those of kalasanty, which was previously mentioned to exhibit better performance than DeepSite, Fpocket, and Concavity. Our results suggest that PUResNet provides a better prediction than kalasanty. In K-fold experiment, PUResNet has a success rate of 61% whereas kalasanty has a success rate of 51%. The results from independent test sets (Coach420 and BU48) revealed that PUResNet exhibits better accuracy than kalasanty (PUResNet had success rates of 53% and 62%, respectively, in Coach420 and BU48, whereas kalasanty had 51% and 57%, respectively). It is important to note that although PUResNet is trained with approximately 1/3 of the dataset that was used to train kalasanty, we were able to exceed kalasanty in terms of performance while evaluating K-fold as well as in independent tests. The model was developed in Python using the Keras library. All the information regarding the use of the trained model is publicly available at https://github.com/jivankandel/PUResNet, along with the trained model and all datasets used in this work. Predicted sites are provided in a mol2 file and can be visualized using different software, such as PYMOL. This work can be further improved by using a sequence alignment tool before calculating similarity using our method which will remove the step of taking maximum over shifted sequences, representing the protein structures along with water molecules, as well as differentiating the surface residue and incorporating the depth of the residues.

## Supplementary Information

**Additional file 1. **Feature visualization. Includes 3D plot of different features used in the study

**Additional file 2.**Model description. Includes description of different model blocks with figures.

**Additional file 3. **KFold training and validation results. Includes validation, training graph, success rate graph and histogram of DVO of different folds

**Additional file 4. **Miscellaneous results.

## Data Availability

All the codes and datasets related to this work are are publicly available at https://github.com/jivankandel/PUResNet. Training dataset: Cleaned training data: https://github.com/jivankandel/PUResNet/blob/main/scpdb_subset.zip. Independent dataset: BU48: https://github.com/jivankandel/PUResNet/blob/main/BU48.zip Coach420: https://github.com/jivankandel/PUResNet/blob/main/coach.zip. Model Code: https://github.com/jivankandel/PUResNet/blob/main/ResNet.py. Model weights: https://github.com/jivankandel/PUResNet/blob/main/whole_trained_model1.hdf. Usage Information: https://github.com/jivankandel/PUResNet/blob/main/README.md

## Abbreviations

PDB: Protein Data Bank
MIF: Molecular Interaction Fields
SVM: Support Vector Machine
MACCS: Molecular ACCess System
DCC: Distance Center Center
DVO: Discretized Volume Overlap
PLI: Proportion of Ligand Inside
$\mathrm{V}_{\mathrm{L}}$: Volume of Ligand
$\mathrm{V}_{\mathrm{pbs}}$: Volume of Predicted Binding Site
$V_{\mathrm{abs}}$: Volume of Actual Binding Site
TP: True Positive
FP: False Positive
FP: False Negative
Avg: Average
ATP: ADENOSINE-5’-TRIPHOSPHATE
