# Supplementary material for: PUResNet: prediction of protein-ligand binding sites using deep residual neural network
Source: J Cheminform. 2021 Sep 8;13:65. doi: 10.1186/s13321-021-00547-7 (PMC8424938; doi:10.1186/s13321-021-00547-7)
Supplement: Supplementary file 1 — Additional file 1. Feature visualization. Includes 3D plot of different features used in the study [file 13321_2021_547_MOESM1_ESM.docx]

PUResNet: Predicting protein-ligand binding sites using deep convolutional neural network.

Jeevan Kandel^1^, Hilal Tayara^2*^, and Kil To Chong^3*^

**Additional File 1**

Contents

[List of Figures 2](#_Toc70500006)

[Data Representation 3](#_Toc70500007)

# List of Figures

[Figure 1S: Figure showing atom’s hybridization in the protein structure 1A80. 3](#_Toc70499994)

[Figure 2S: Figure showing hydrophobic and hydrophilic atoms in the protein structure 1A80. 4](#_Toc70499995)

[Figure 3S: Figure showing partial charge of an atom in the protein structure 1A80. 5](#_Toc70499996)

[Figure 4S: Figure showing number of heteroatoms attached to an atom in the protein structure 1A80. 6](#_Toc70499997)

[Figure 5S: Figure showing number of non-hydrogens (heavy atoms) attached to an atom in the protein structure 1A80. 7](#_Toc70499998)

[Figure 6S: Figure showing non-acceptor and acceptor atoms in the Protein Structure 1A80. 8](#_Toc70499999)

[Figure 7S: Figure showing aliphatic and aromatic atoms in the protein structure 1A80. 9](#_Toc70500000)

[Figure 8S: Figure showing donor and non-donor atoms in the protein structure 1A80. 10](#_Toc70500001)

[Figure 9S: Figure showing atoms in and not in ring in the protein structure 1A80. 11](#_Toc70500002)

# Data Representation

3D plot of 1A80 protein structure showing different properties used in study.


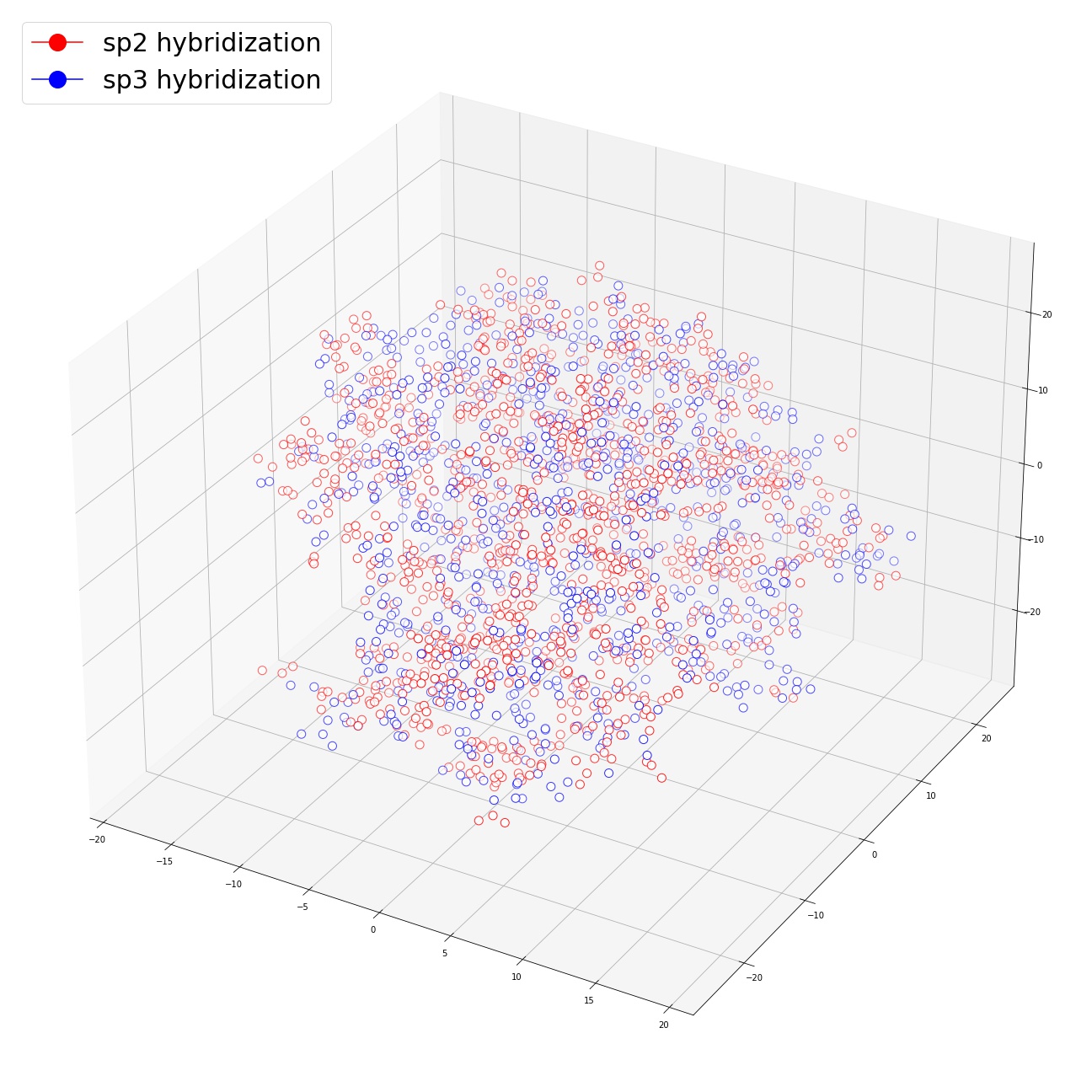


Figure 1S: Figure showing atom’s hybridization in the protein structure 1A80.


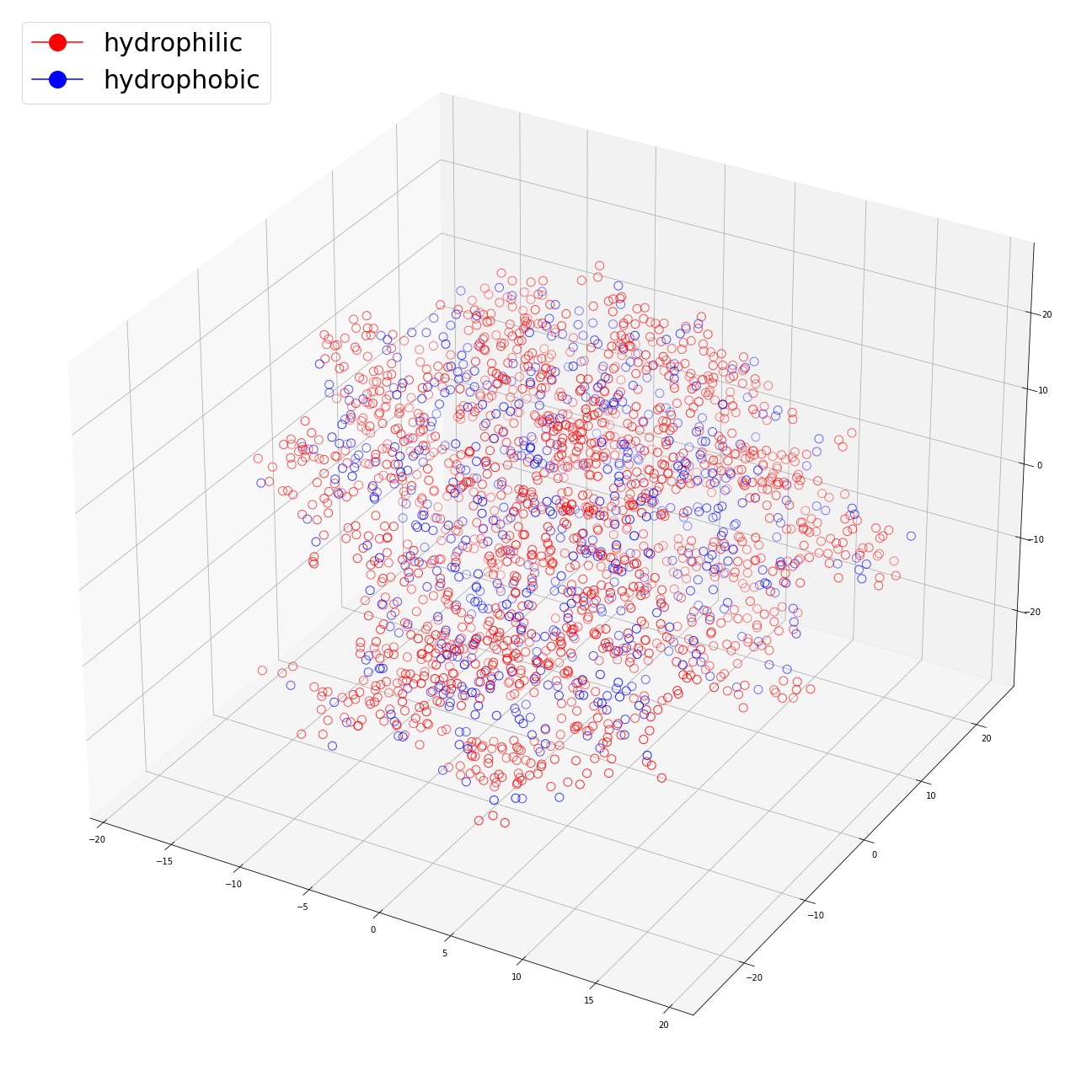


Figure 2S: Figure showing hydrophobic and hydrophilic atoms in the protein structure 1A80.


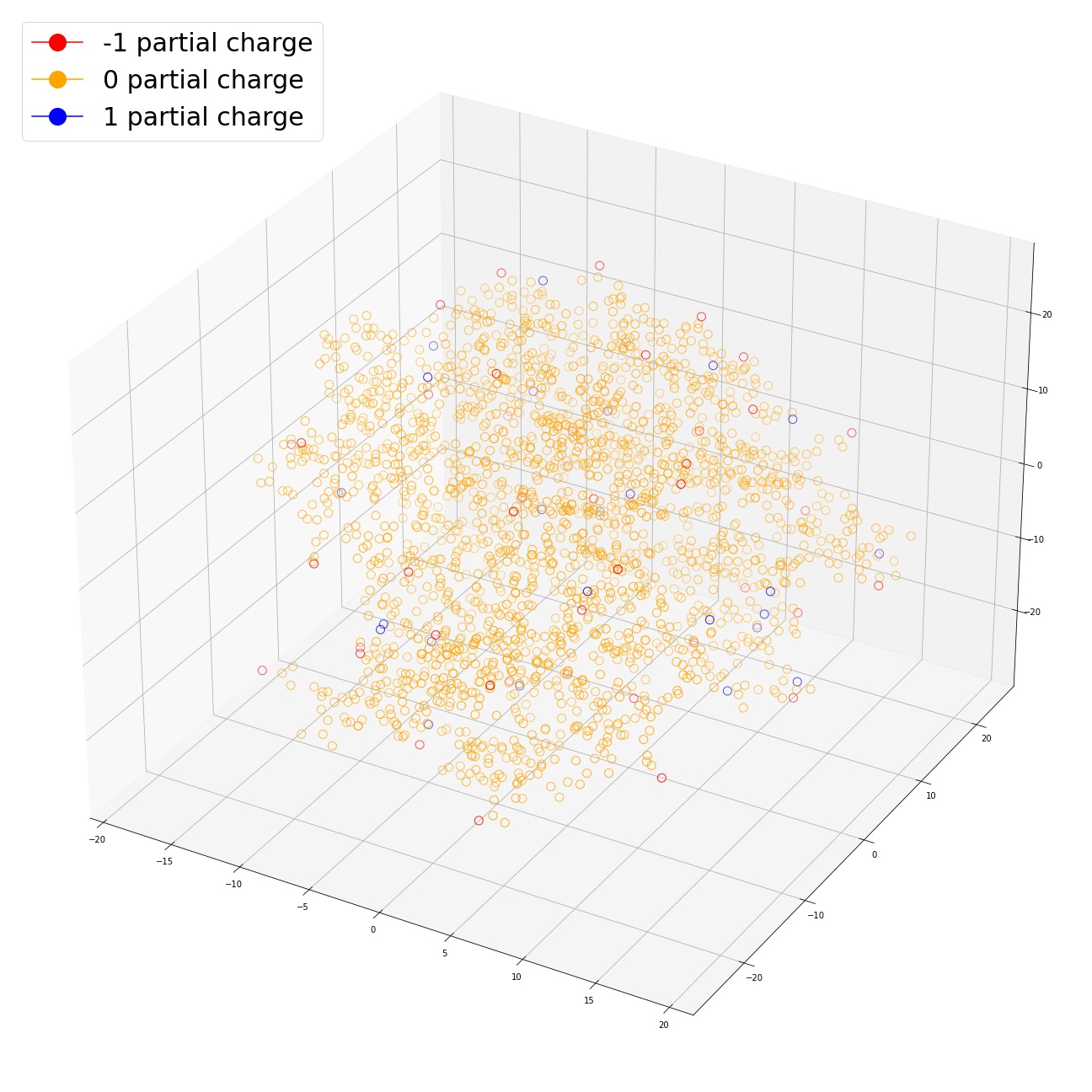


Figure 3S: Figure showing partial charge of an atom in the protein structure 1A80.


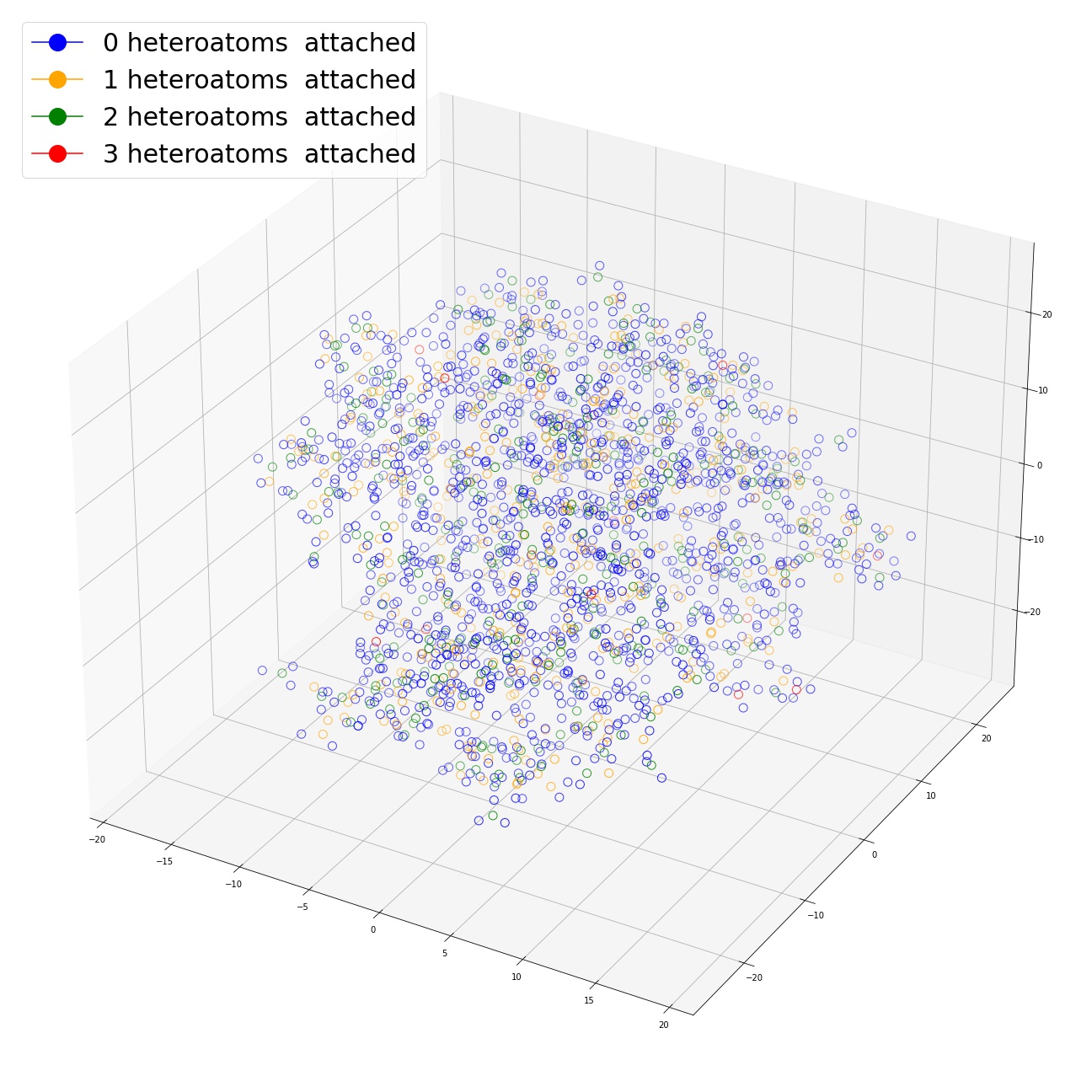


Figure 4S: Figure showing number of heteroatoms attached to an atom in the protein structure 1A80.


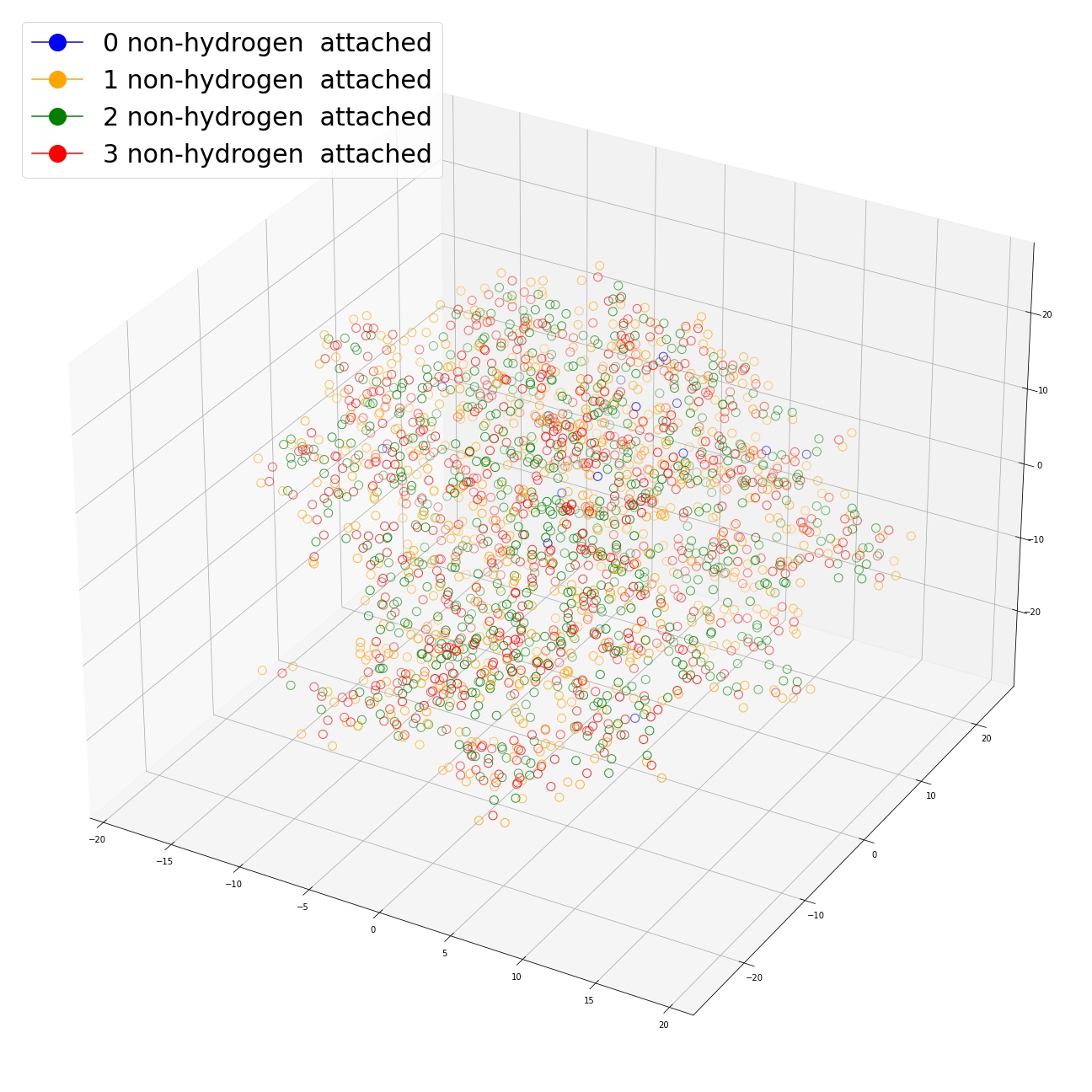


Figure 5S: Figure showing number of non-hydrogens (heavy atoms) attached to an atom in the protein structure 1A80.


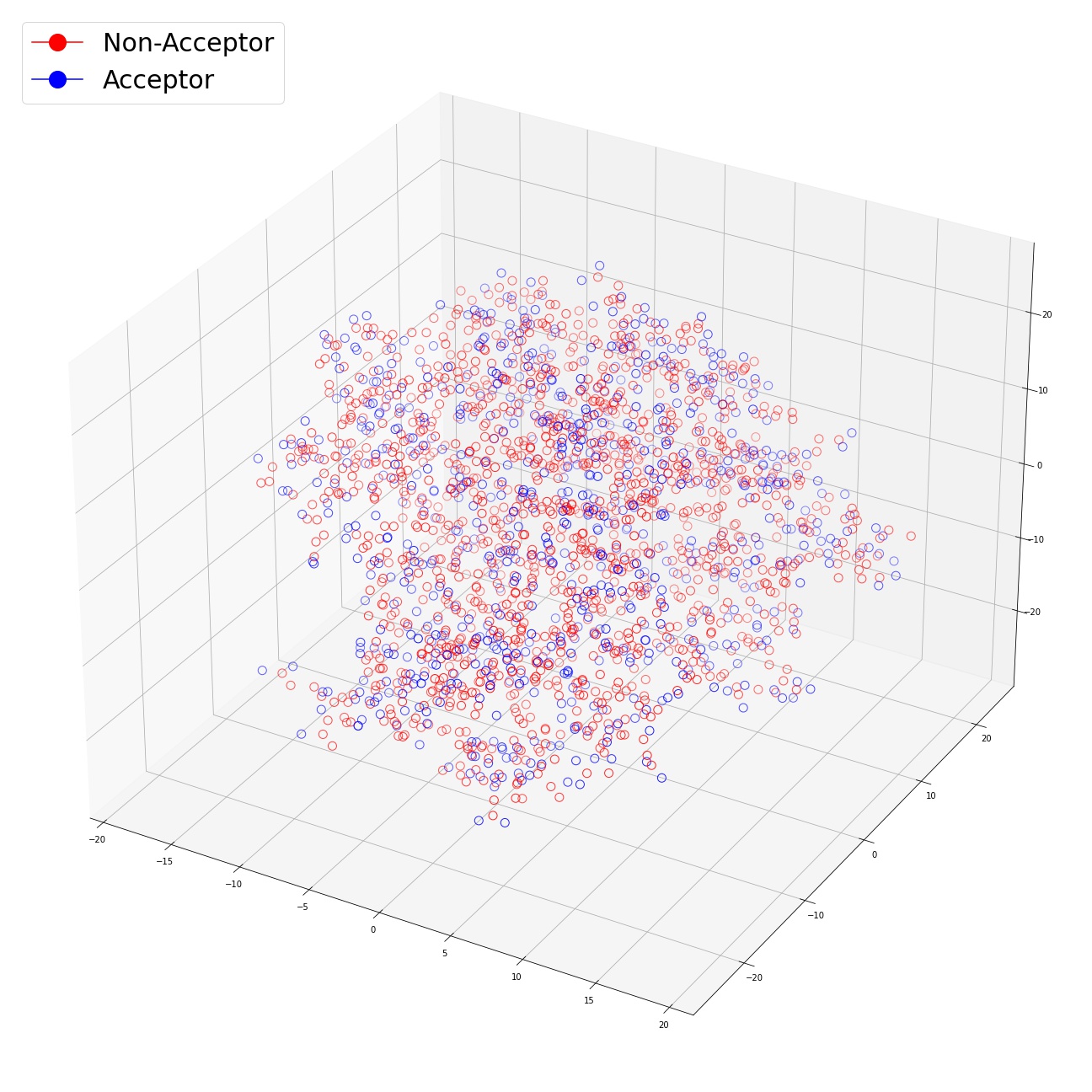


Figure 6S: Figure showing non-acceptor and acceptor atoms in the Protein Structure 1A80.


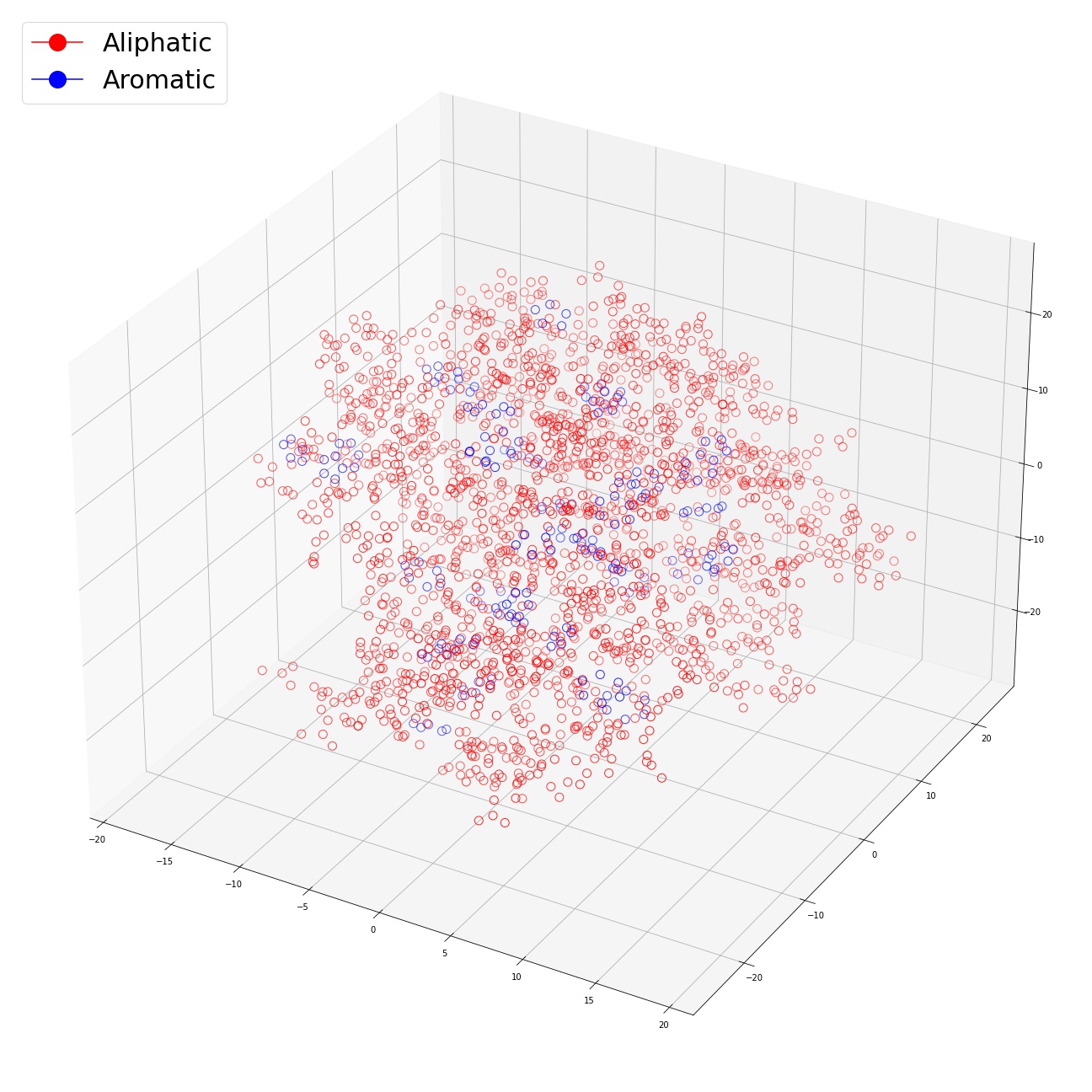


Figure 7S: Figure showing aliphatic and aromatic atoms in the protein structure 1A80.


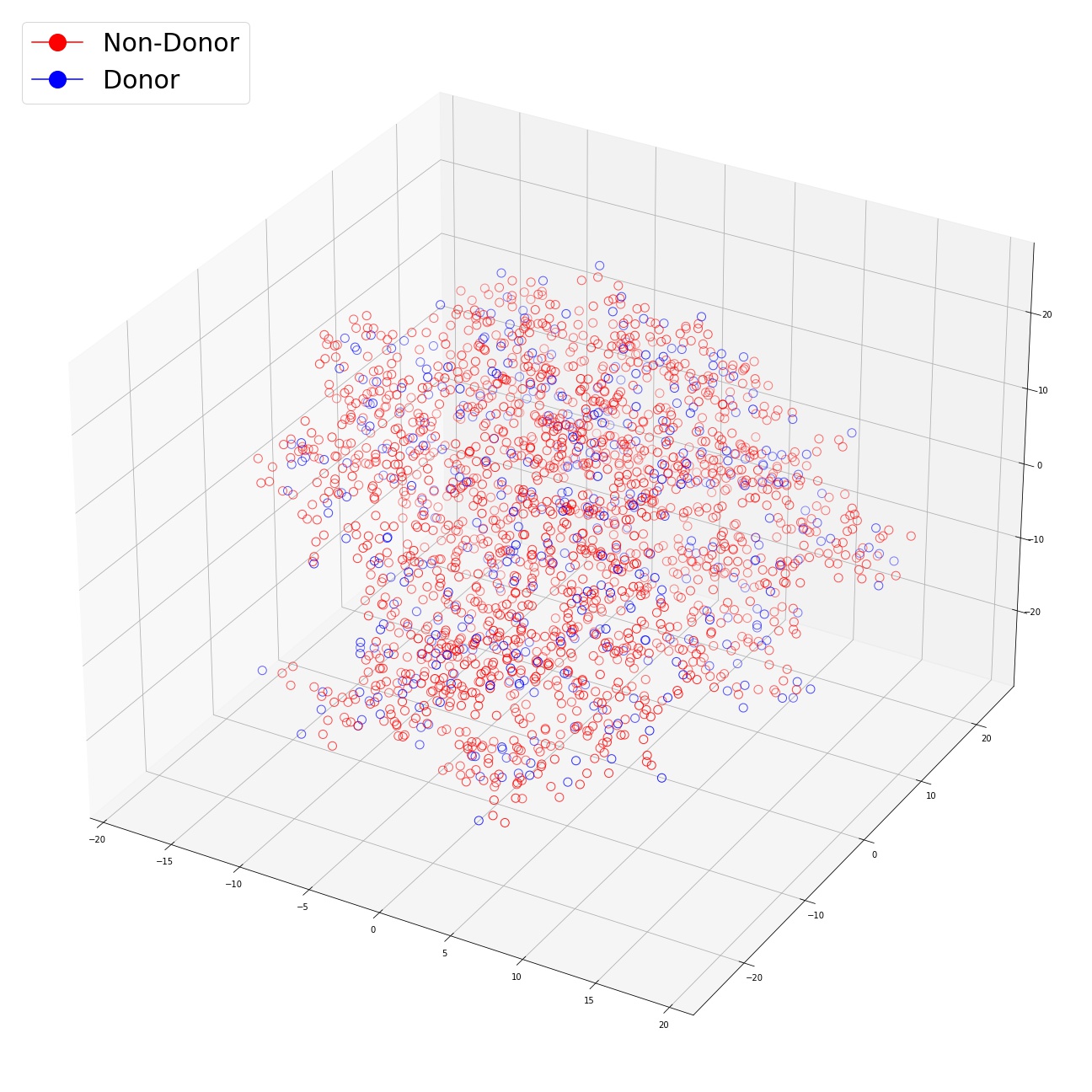


Figure 8S: Figure showing donor and non-donor atoms in the protein structure 1A80.


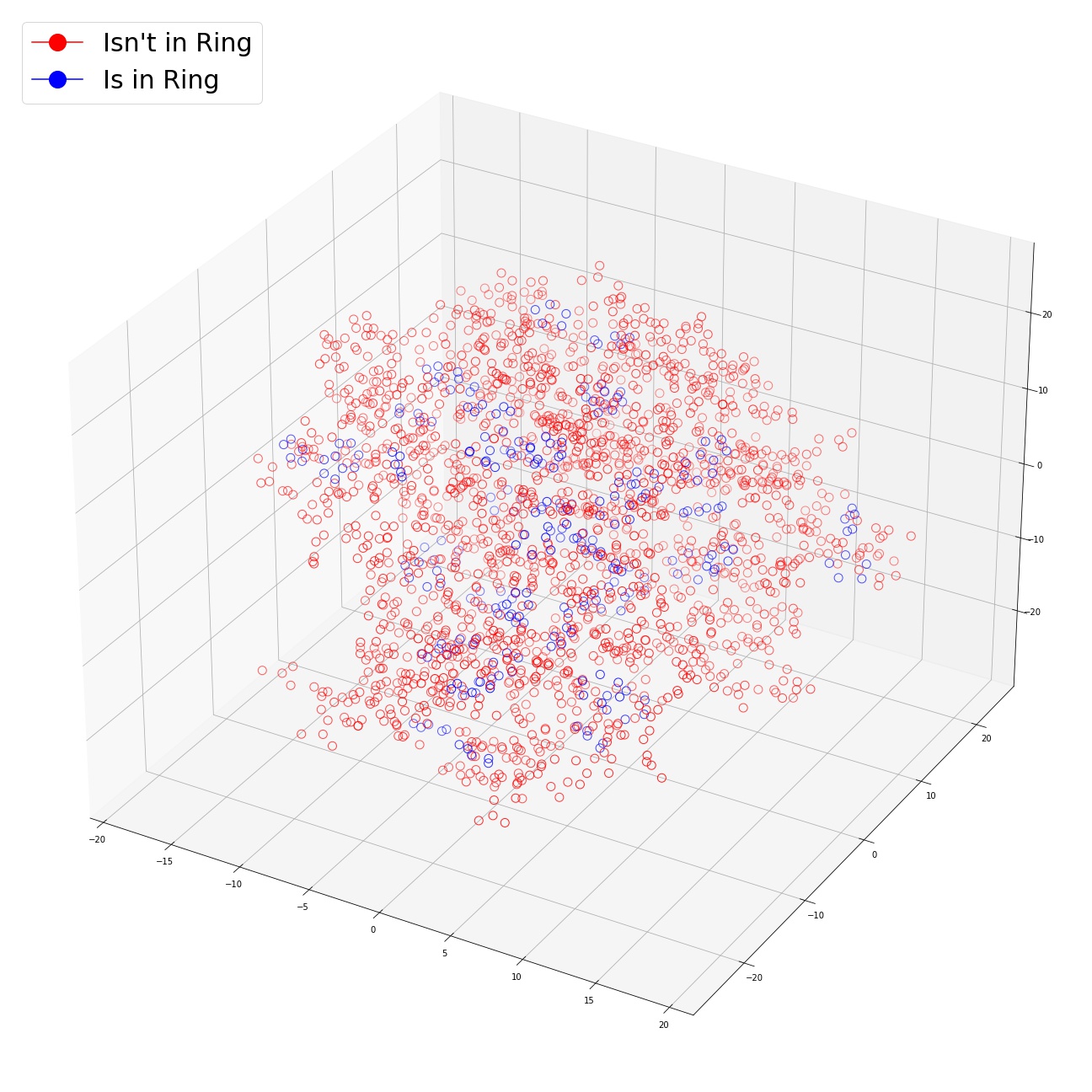


Figure 9S: Figure showing atoms in and not in ring in the protein structure 1A80.
