# Supplementary material for: PUResNet: prediction of protein-ligand binding sites using deep residual neural network
Source: J Cheminform. 2021 Sep 8;13:65. doi: 10.1186/s13321-021-00547-7 (PMC8424938; doi:10.1186/s13321-021-00547-7)
Supplement: Supplementary file 2 — Additional file 2.Model description. Includes description of different model blocks with figures. [file 13321_2021_547_MOESM2_ESM.docx]

PUResNet: Predicting protein-ligand binding sites using deep convolutional neural network.

Jeevan Kandel^1^, Hilal Tayara^2*^, and Kil To Chong^3*^

**Additional File 2**

Contents

[List of Figures 2](#_Toc70500091)

[List of Tables 2](#_Toc70500092)

[Model 3](#_Toc70500093)

[Convolution Block 3](#_Toc70500094)

[Identity Block 4](#_Toc70500095)

[Up Sampling Block 5](#_Toc70500096)

# List of Figures

[Figure 1S: Convolution block of PUResNet model 3](#_Toc70500082)

[Figure 2S: Identity block of PUResNet model 4](#_Toc70500083)

[Figure 3S: Up sampling block of PUResNet Model 5](#_Toc70500084)

[Figure 4S: PUResNet model with input and output size of each block. 6](#_Toc70500085)

# List of Tables

[Table 1S: Table showing number of filters and strides/pool size in each block of PUResNet. 7](#_Toc70438400)

# Model

PUResNet consists of three basic blocks convolution block, identity block, and up sampling block. The convolution Block shown in Figure 1S is a 12-layered block consisting of 3d convolution, batch normalization, add, and activation layer. The identity block shown in Figure 2S has the same type of layer except it is a 10-layered block. Up sampling block shown in Figure 3S is a 14-layered block consisting additional 3d up sampling layer. Each block is equipped with a shortcut path.

## Convolution Block


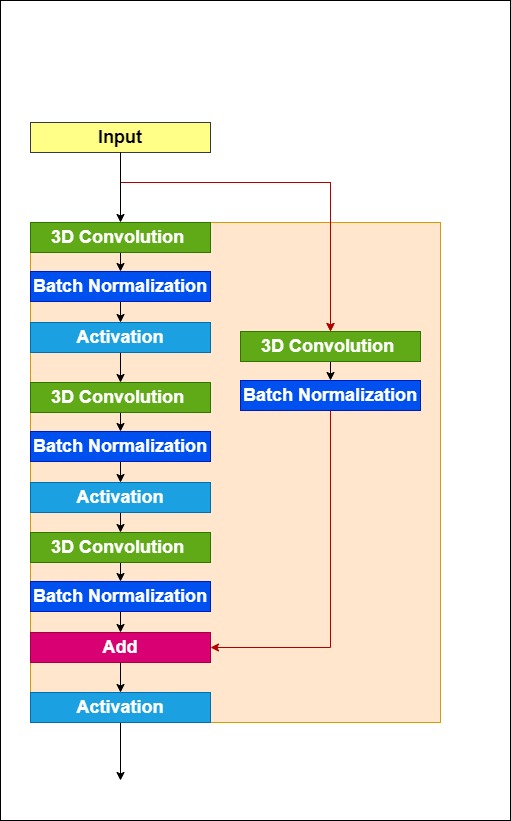


Figure 1S: Convolution block of PUResNet model

Convolution Block shown in figure 1S has two pair of 3D convolution, batch normalization and relu activation layer followed by a pair of 3D convolution, batch normalization, add and relu activation layer and is equipped with a shortcut path which passed through a pair of 3D convolution and batch normalization layer as shown in Figure 1S. First, third and skip path 3D Convolution layer has kernel size of 1x1x1 whereas second has kernel size of 3x3x3. For the first and shortcut path, 3D convolution stride size (as in table 1) is given as an input parameter to the block for adjusting output size as shown in figure 4 whereas the second and third have stride size of 1x1x1. The number of filters for each 3D convolution is equal to one-third of the number of filters (given in table 1) passed to the block as an input parameter.

## Identity Block


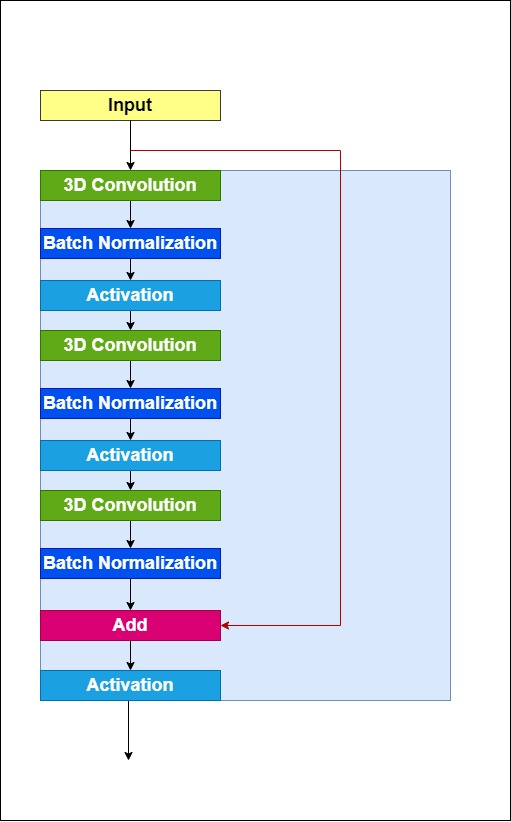


Figure 2S: Identity block of PUResNet model

Identity Block shown in figure 2 is identical to convolution block except shortcut path is not passed through a pair of 3D convolution and batch normalization.

## Up Sampling Block


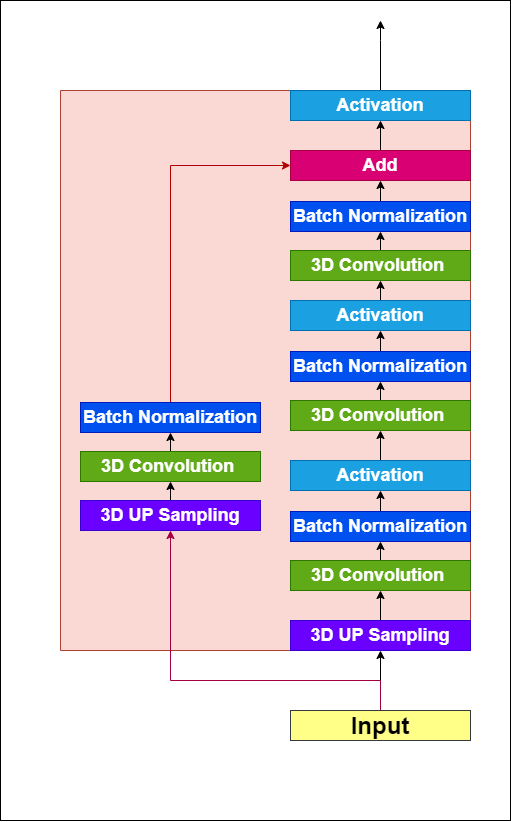


Figure 3S: Up sampling block of PUResNet model

Up Sampling block shown in figure 3S is inspired from convolution block and is almost similar except there is an additional 3D up sampling layer. All 3d convolution layer has a stride of size 1x1x1 and 3d up sampling layer pool size (shown is table 1S) is given as an input parameter to the block for adjusting the output size as shown as in figure 4S.


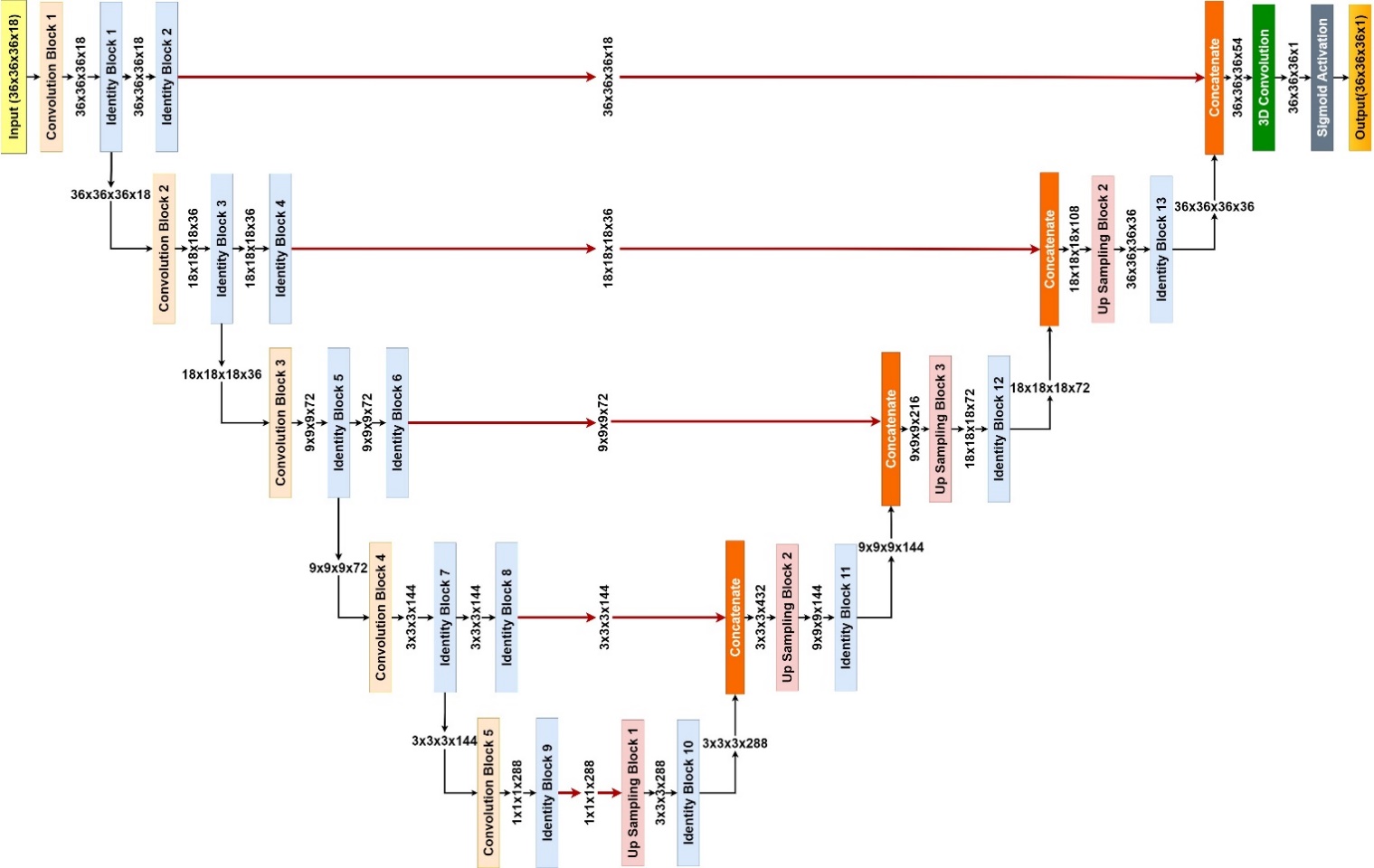


Figure 4S: PUResNet model with input and output size of each block.

| **Block** | **Number of filters** | **Stride / Pool_Size** |
| --- | --- | --- |
| Convolution Block 1 | 54 | (1,1,1) |
| Identity Block 1 | 54 | (1,1,1) |
| Identity Block 2 | 54 | (1,1,1) |
| Convolution Block 2 | 108 | (2,2,2) |
| Identity Block 3 | 108 | (1,1,1) |
| Identity Block 4 | 108 | (1,1,1) |
| Convolution Block 3 | 216 | (2,2,2) |
| Identity Block 5 | 216 | (1,1,1) |
| Identity Block 6 | 216 | (1,1,1) |
| Convolution Block 4 | 432 | (3,3,3) |
| Identity Block 6 | 432 | (1,1,1) |
| Identity Block 7 | 432 | (1,1,1) |
| Convolution Block 5 | 864 | (3,3,3) |
| Identity Block 8 | 864 | (1,1,1) |
| Identity Block 9 | 864 | (1,1,1) |
| Up Sampling Block 1 | 864 | (3,3,3) |
| Identity Block 10 | 864 | (1,1,1) |
| Up Sampling Block 2 | 432 | (3,3,3) |
| Identity Block 11 | 432 | (1,1,1) |
| Up Sampling Block 3 | 216 | (2,2,2) |
| Identity Block 12 | 216 | (1,1,1) |
| Up Sampling Block 4 | 108 | (2,2,2) |
| Identity Block 13 | 108 | (1,1,1) |
| Convolution3D | 1 | (1,1,1) |

Table 1S: Table showing number of filters and strides/pool size in each block of PUResNet.
