# Supplementary material for: PUResNet: prediction of protein-ligand binding sites using deep residual neural network
Source: J Cheminform. 2021 Sep 8;13:65. doi: 10.1186/s13321-021-00547-7 (PMC8424938; doi:10.1186/s13321-021-00547-7)
Supplement: Supplementary file 3 — Additional file 3. KFold training and validation results. Includes validation, training graph, success rate graph and histogram of DVO of different folds [file 13321_2021_547_MOESM3_ESM.docx]

PUResNet: Predicting protein-ligand binding sites using deep convolutional neural network.

Jeevan Kandel^1^, Hilal Tayara^2*^, and Kil To Chong^4*^

**Additional File 3**

Contents

[List of Figures 2](#_Toc70438668)

[Results 3](#_Toc70438669)

[K-fold Results 3](#_Toc70438670)

[Fold 1 3](#_Toc70438671)

[Fold 2 5](#_Toc70438672)

[Fold 3 7](#_Toc70438673)

[Fold 4 9](#_Toc70438674)

# List of Figures

[Figure 1S: Validation loss vs training loss (PUResNet) 3](#_Toc78124586)

[Figure 2S: Validation accuracy vs training accuracy (PUResNet) 3](#_Toc78124587)

[Figure 3S: Success Rate plot for different DCC values (Kalasanty Vs PUResNet) 4](#_Toc78124588)

[Figure 4S: Histogram of DVO values for protein structure having DCC ≤ 4Å (Kalasanty Vs PUResNet) 4](#_Toc78124589)

[Figure 5S: Validation loss vs training Loss (PUResNet) 5](#_Toc78124590)

[Figure 6S: Validation accuracy vs training accuracy (PUResNet) 5](#_Toc78124591)

[Figure 7S: Success Rate plot for different DCC values (Kalasanty Vs PUResNet) 6](#_Toc78124592)

[Figure 8S: Histogram of DVO values for protein structure having DCC ≤ 4Å (Kalasanty Vs PUResNet) 6](#_Toc78124593)

[Figure 9S: Validation loss vs training Loss (PUResNet) 7](#_Toc78124594)

[Figure 10S: Validation accuracy vs training accuracy (PUResNet) 7](#_Toc78124595)

[Figure 11S: Success Rate plot for different DCC values (Kalasanty Vs PUResNet) 8](#_Toc78124596)

[Figure 12S: Histogram of DVO values for protein structure having DCC ≤ 4Å (Kalasanty Vs PUResNet) 8](#_Toc78124597)

[Figure 13S: Validation loss vs training loss (PUResNet) 9](#_Toc78124598)

[Figure 14S: Validation accuracy vs training accuracy (PUResNet) 9](#_Toc78124599)

[Figure 15S: Success Rate plot for different DCC values (Kalasanty Vs PUResNet) 10](#_Toc78124600)

[Figure 16S: Histogram of DVO values for protein structure having DCC ≤ 4Å (Kalasanty Vs PUResNet) 10](#_Toc78124601)

# Results

## K-fold Results

### Fold 1


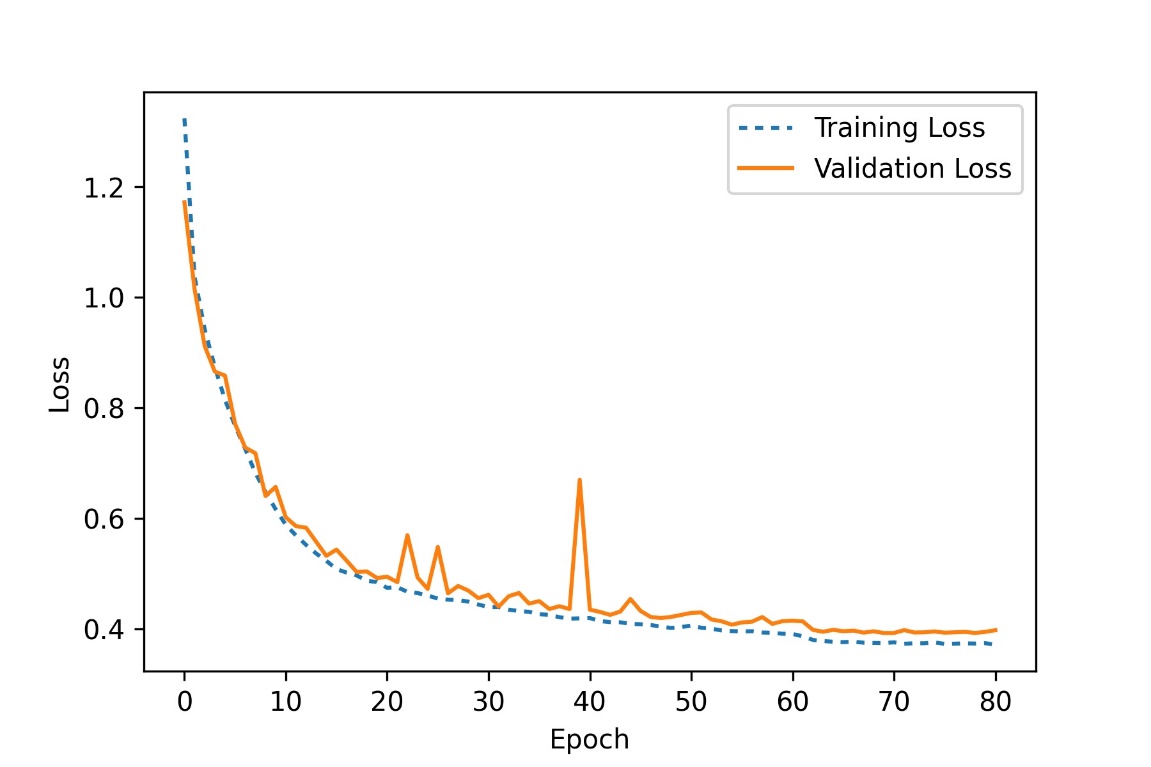


Figure 1S: Validation loss vs training loss (PUResNet)


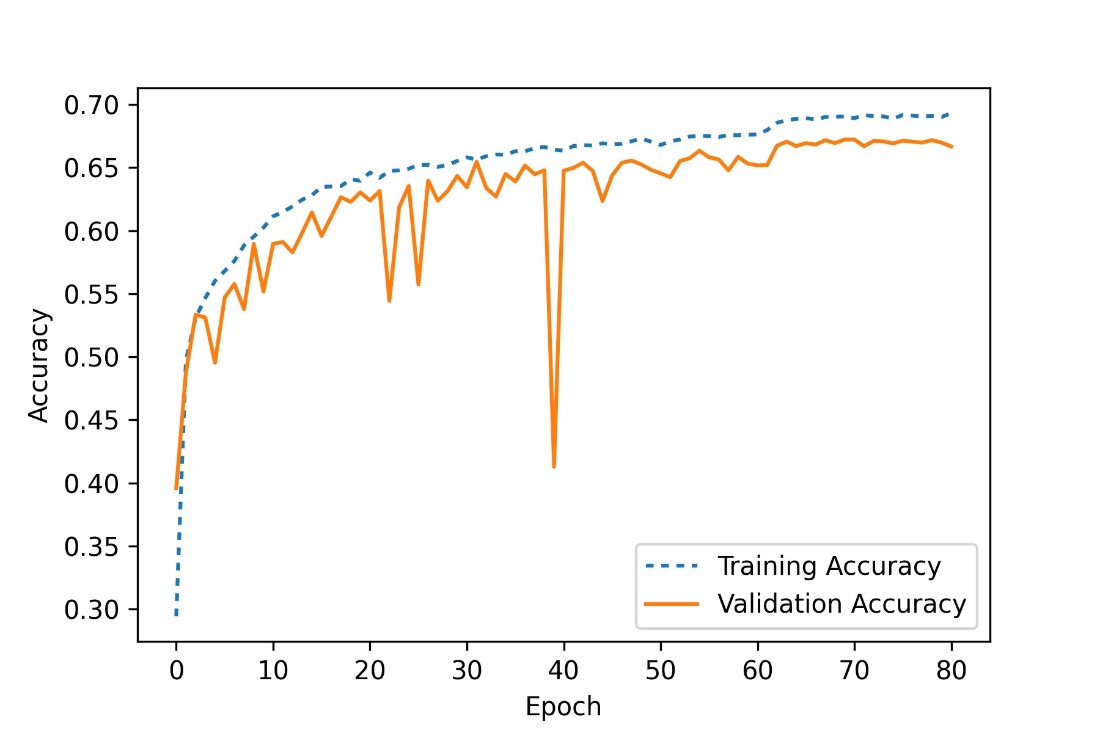


Figure 2S: Validation accuracy vs training accuracy (PUResNet)


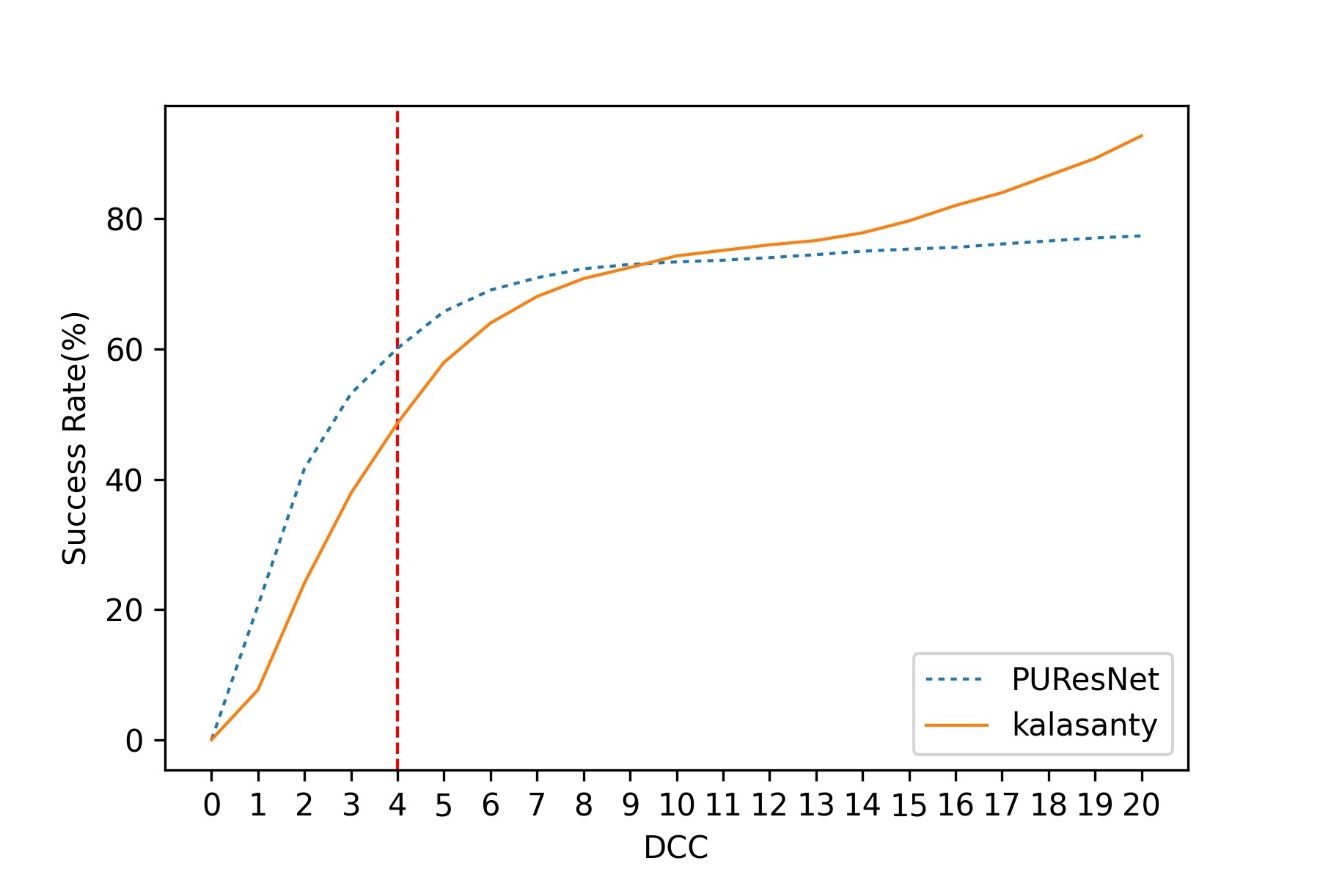


Figure 3S: Success Rate plot for different DCC values (Kalasanty Vs PUResNet)


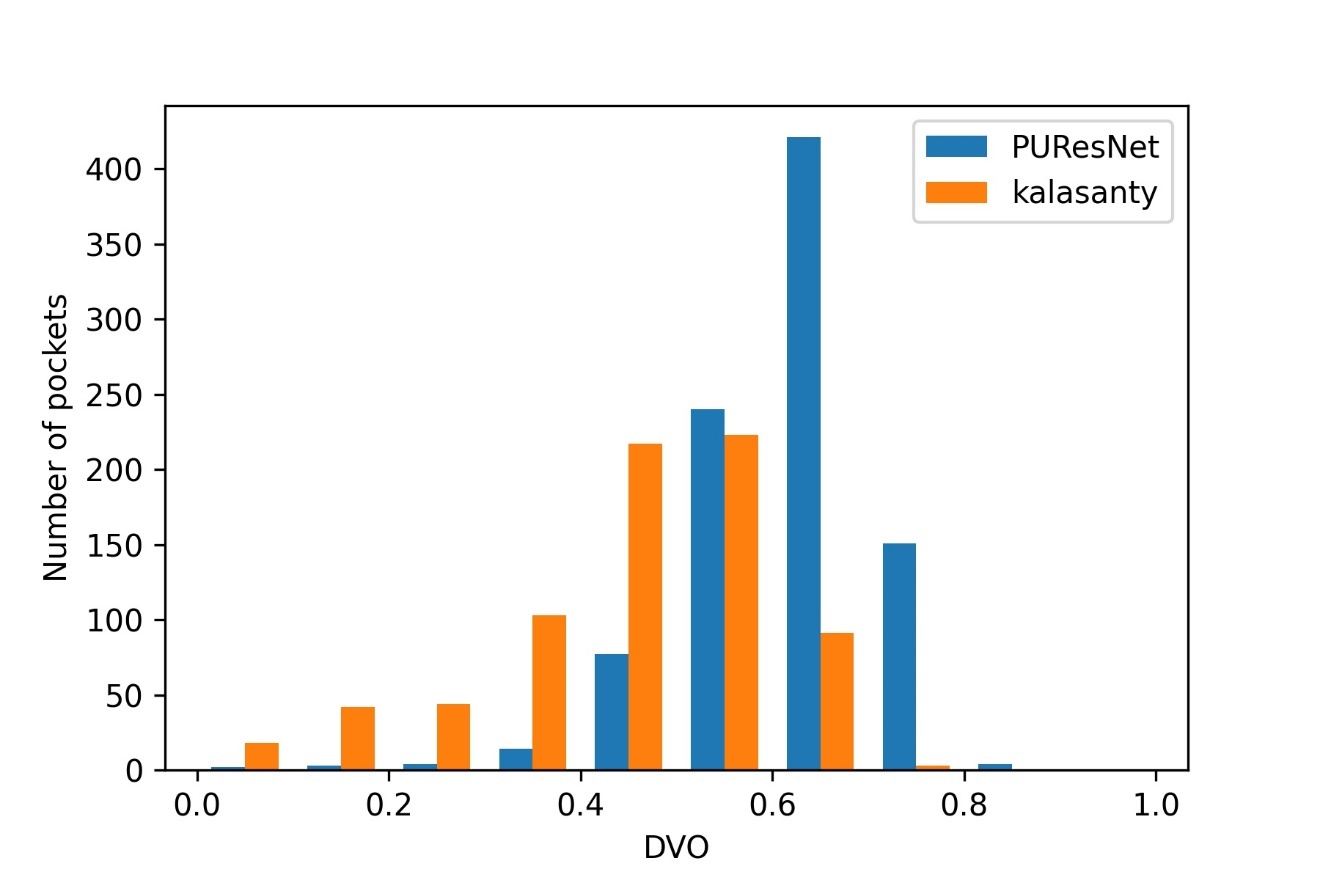


Figure 4S: Histogram of DVO values for protein structure having DCC ≤ 4Å (Kalasanty Vs PUResNet)

### Fold 2


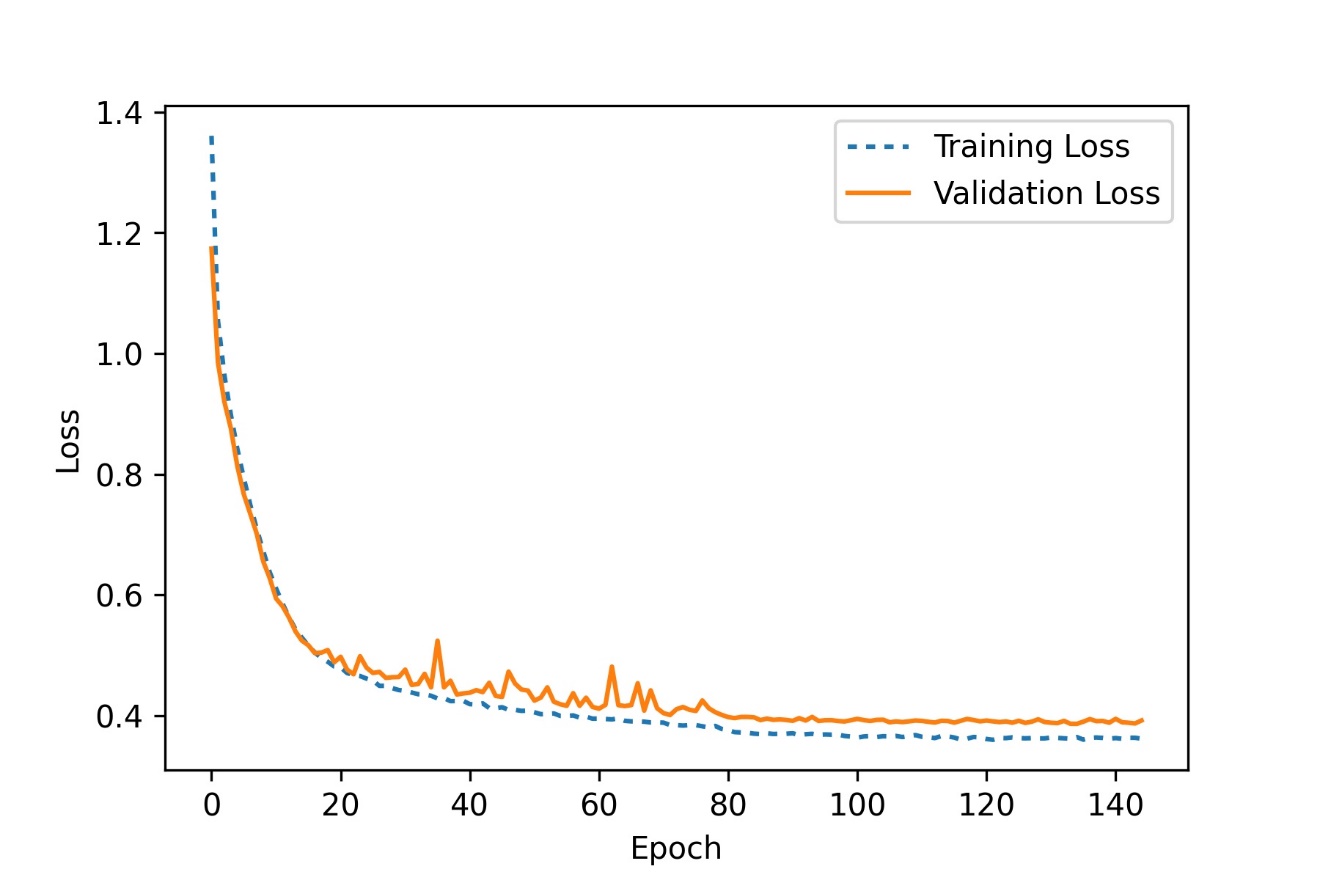


Figure 5S: Validation loss vs training Loss (PUResNet)


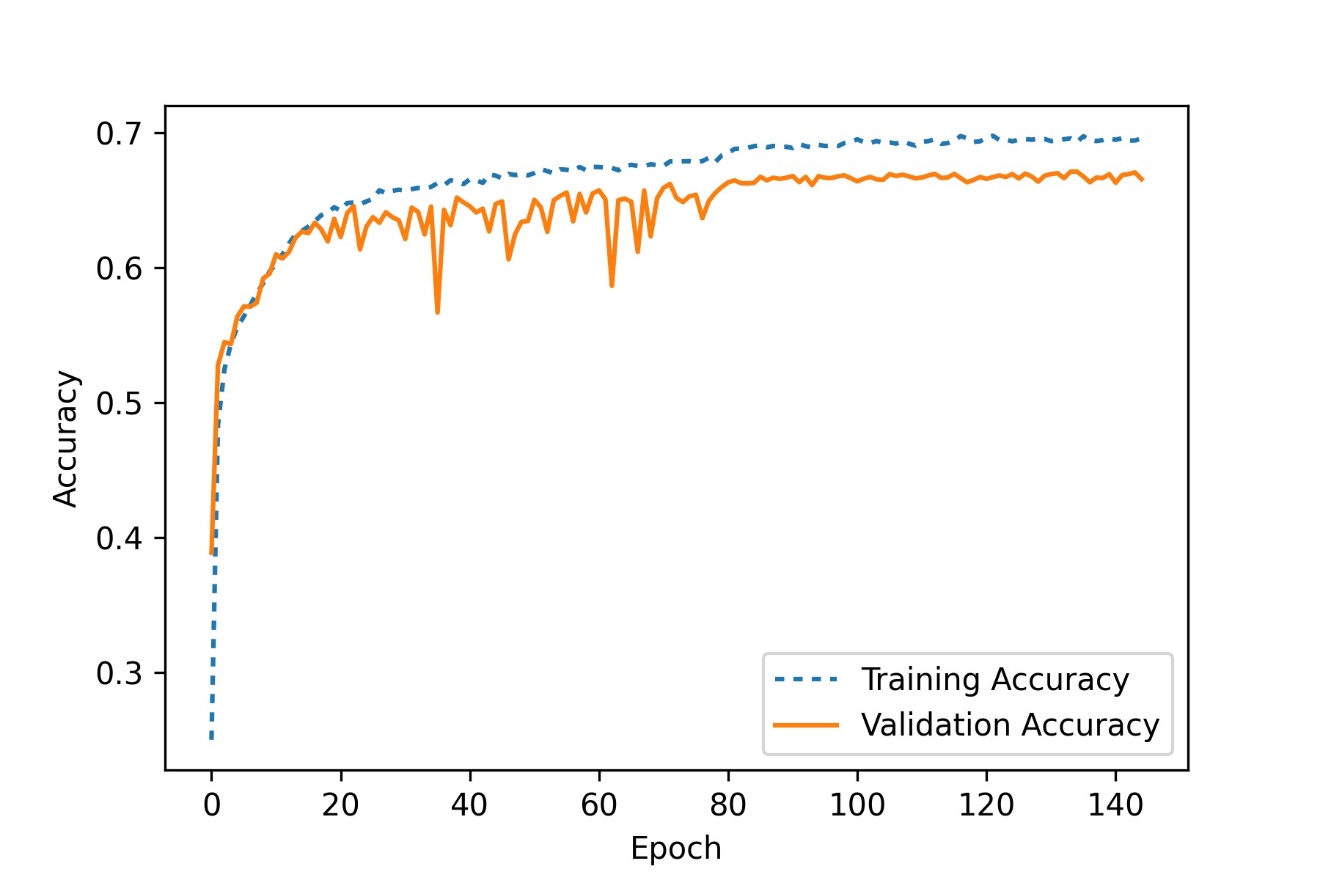


Figure 6S: Validation accuracy vs training accuracy (PUResNet)


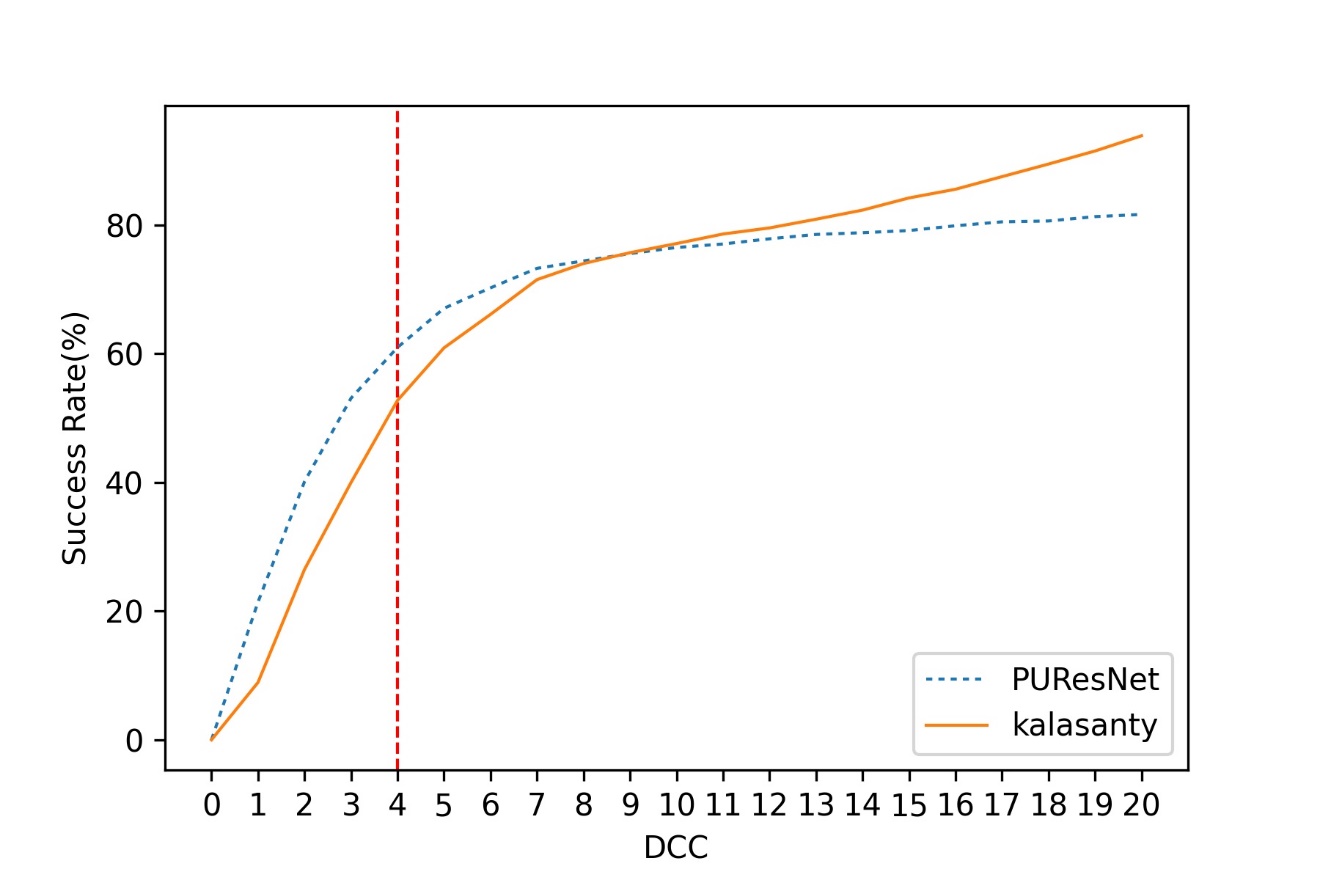


Figure 7S: Success Rate plot for different DCC values (Kalasanty Vs PUResNet)


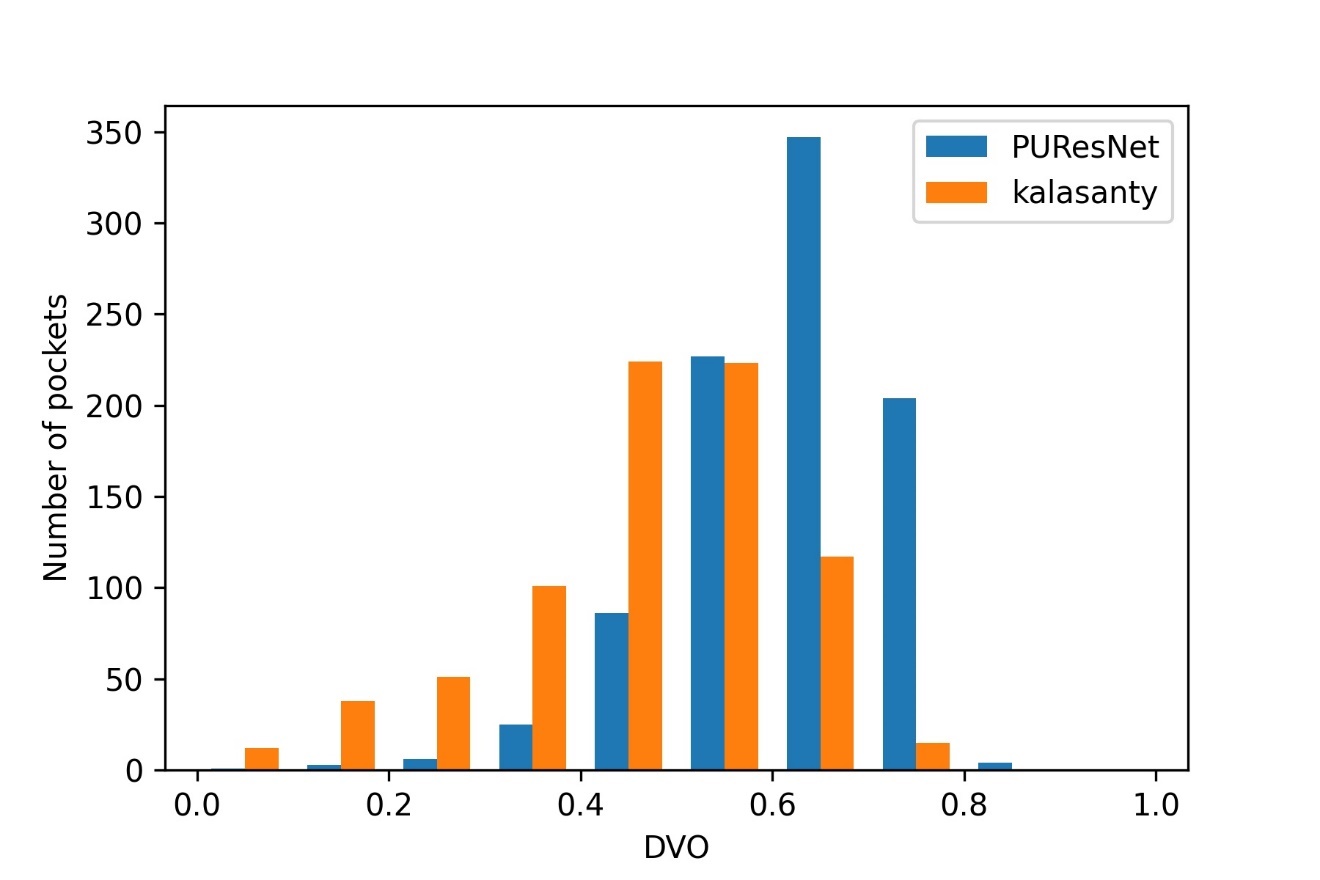


Figure 8S: Histogram of DVO values for protein structure having DCC ≤ 4Å (Kalasanty Vs PUResNet)

### Fold 3


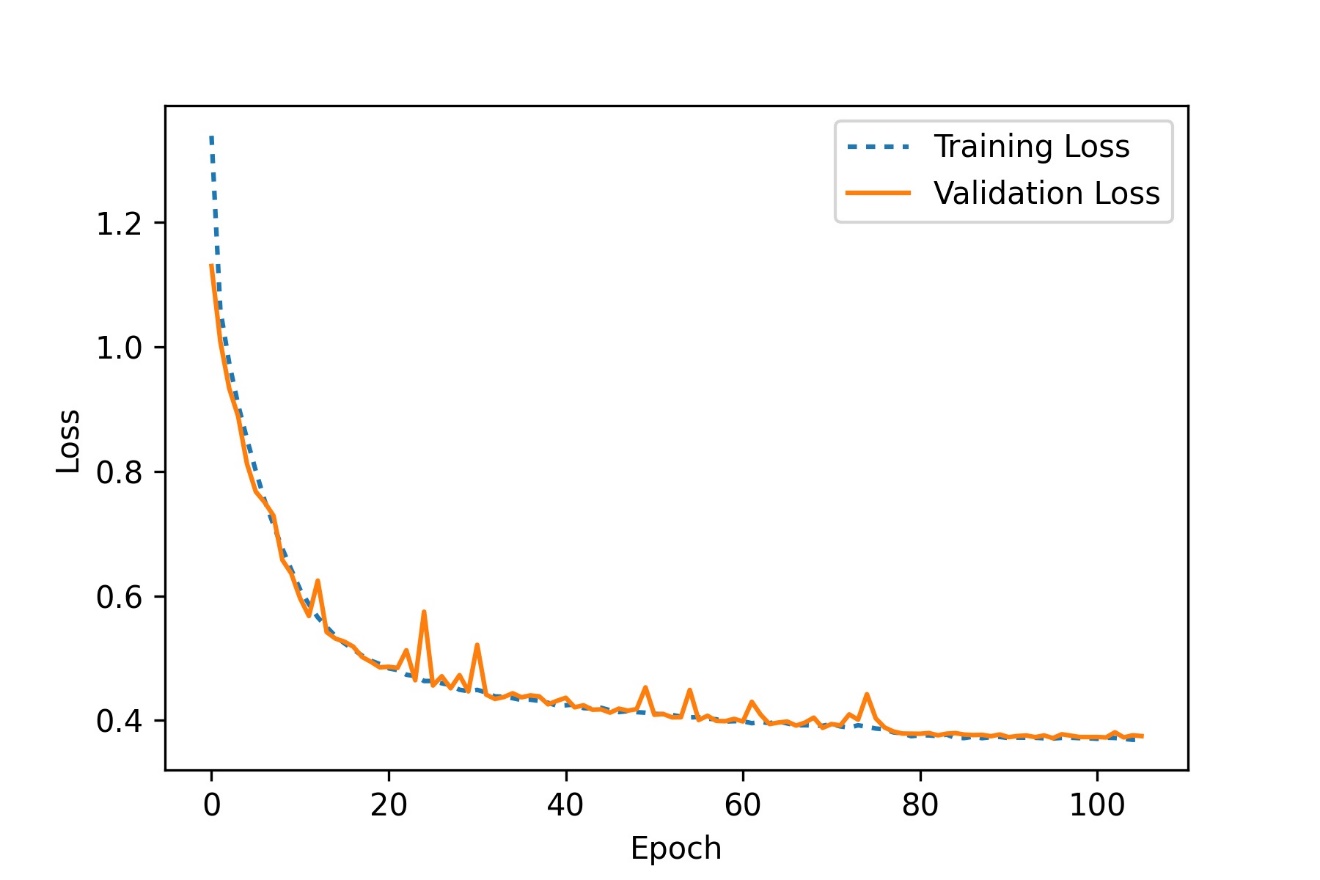


Figure 9S: Validation loss vs training Loss (PUResNet)


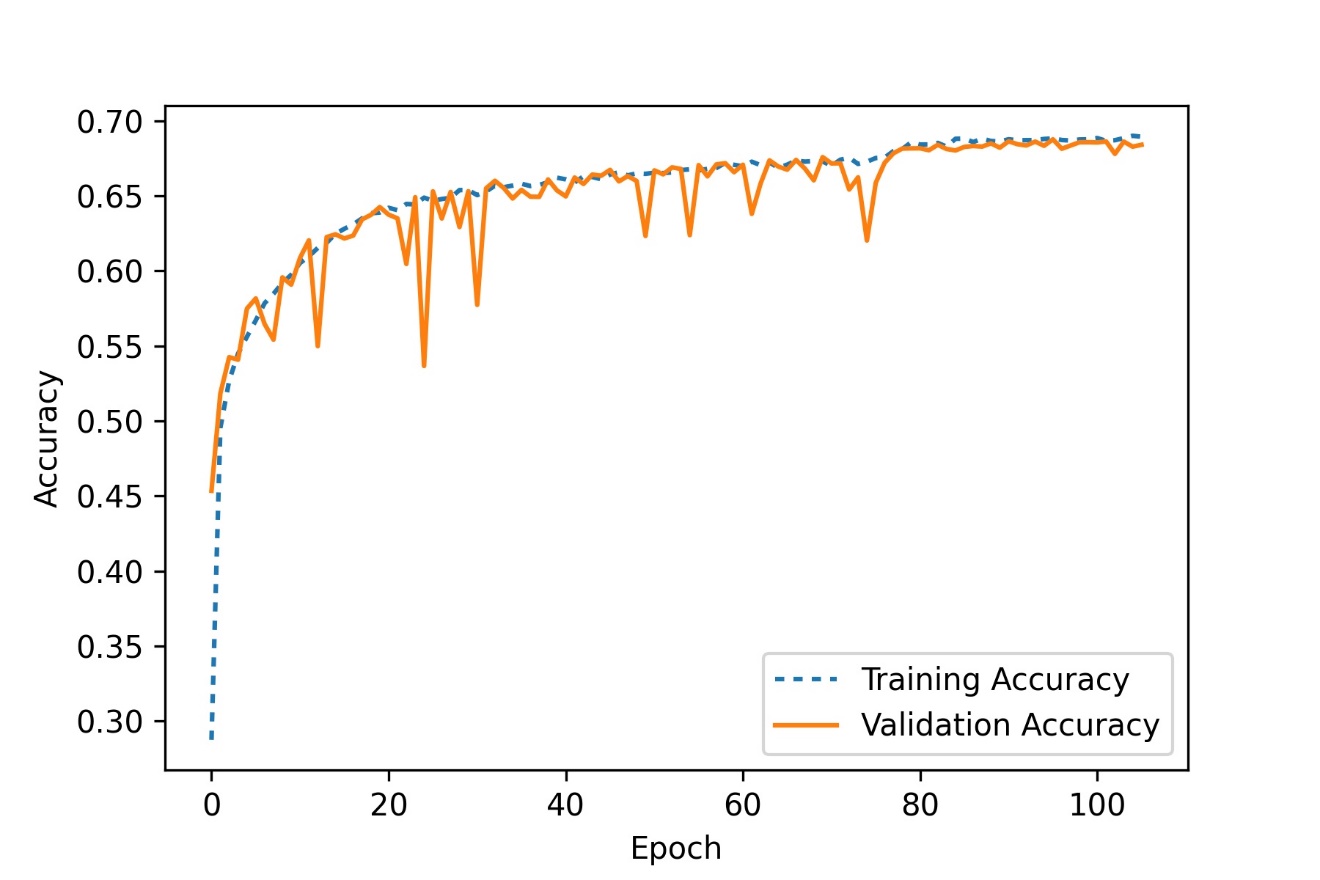


Figure 10S: Validation accuracy vs training accuracy (PUResNet)


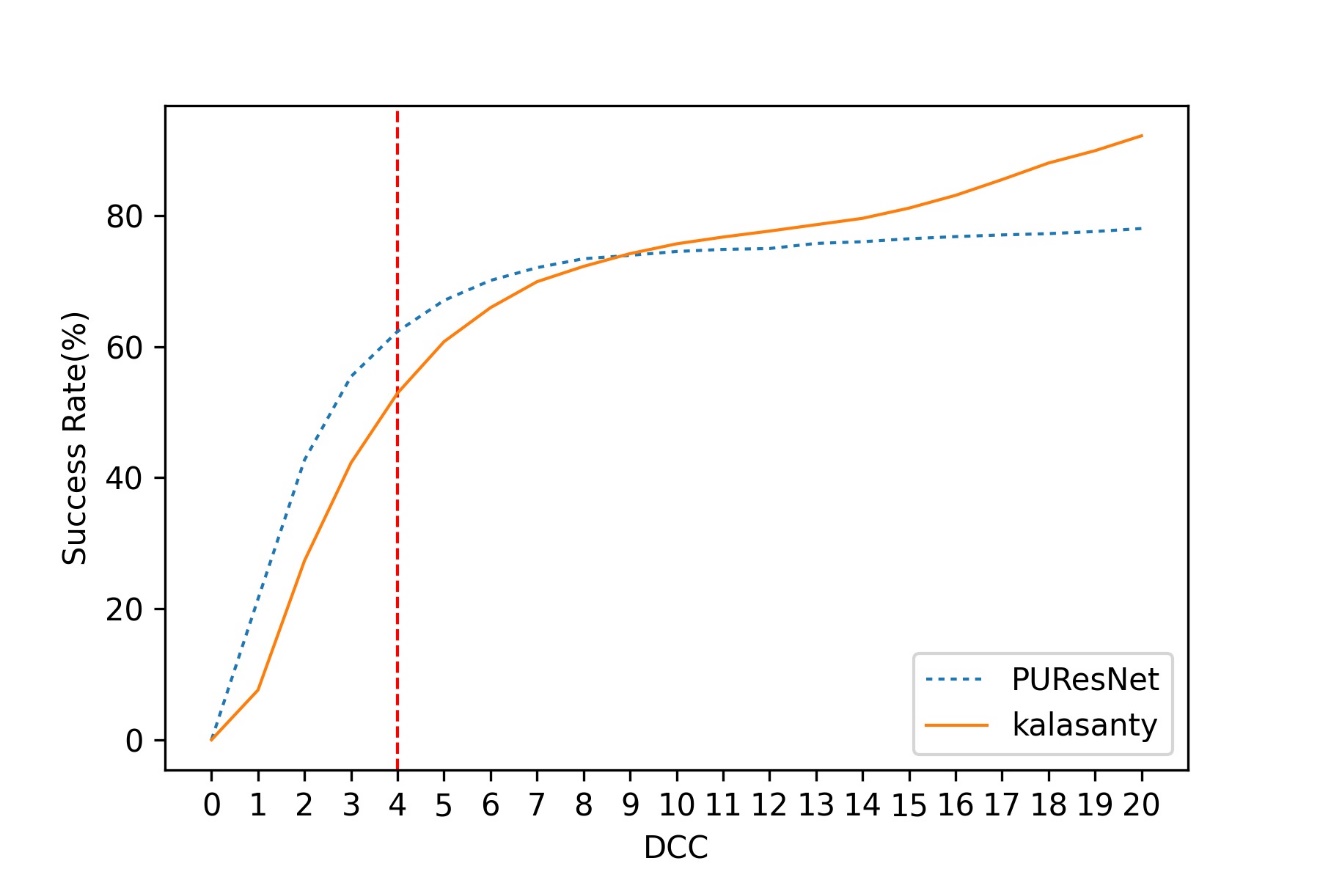


Figure 11S: Success Rate plot for different DCC values (Kalasanty Vs PUResNet)


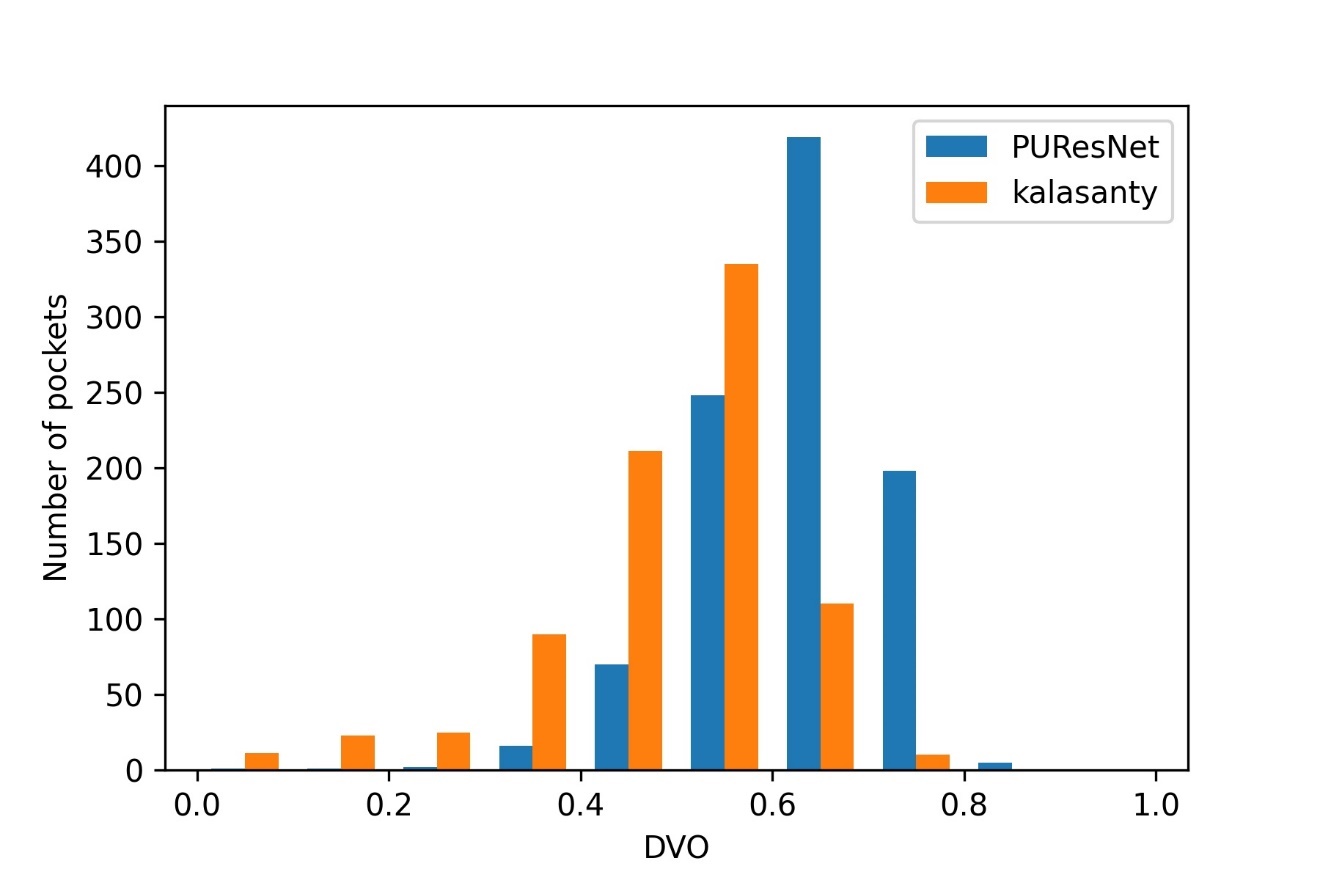


Figure 12S: Histogram of DVO values for protein structure having DCC ≤ 4Å (Kalasanty Vs PUResNet)

### Fold 4


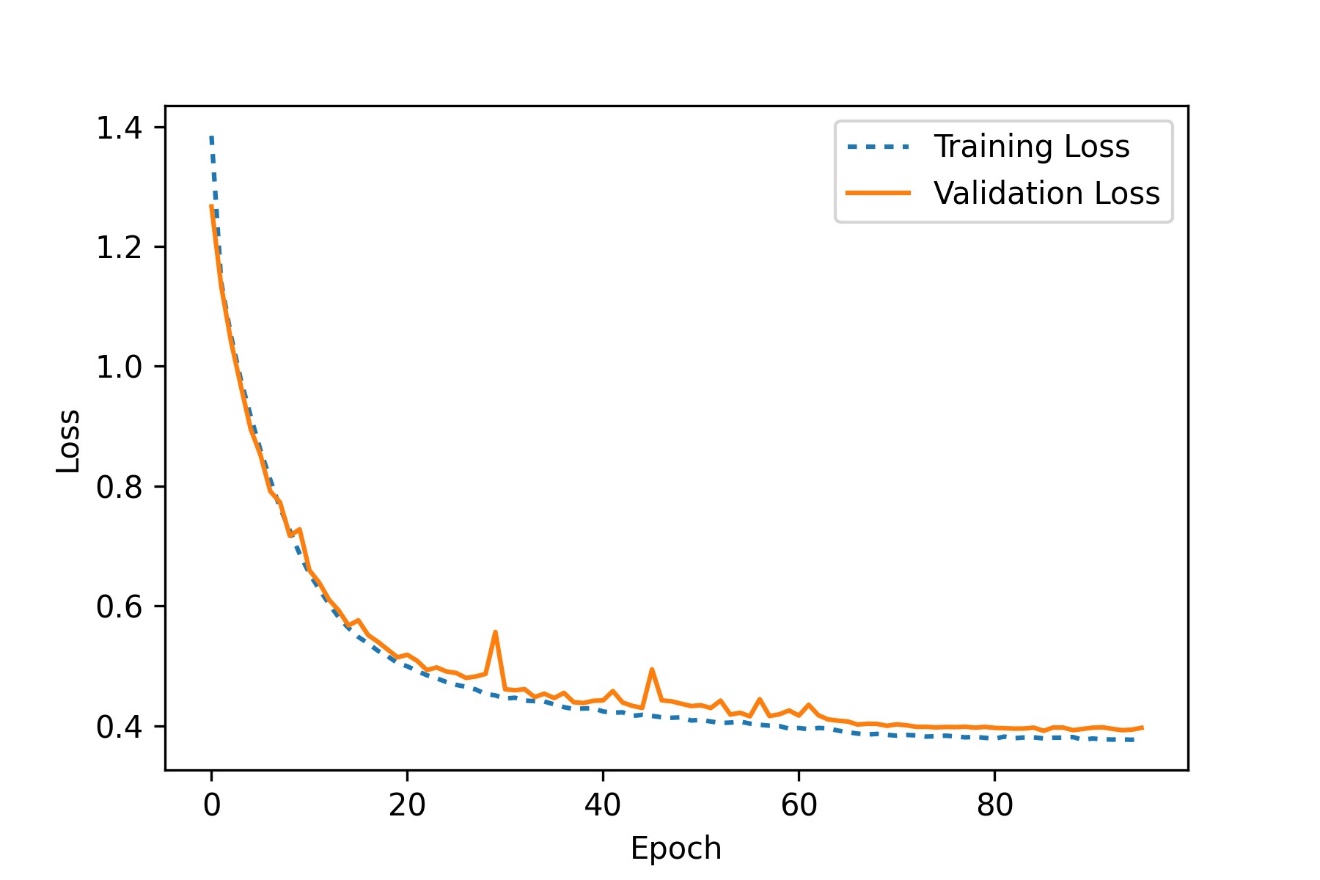


Figure 13S: Validation loss vs training loss (PUResNet)


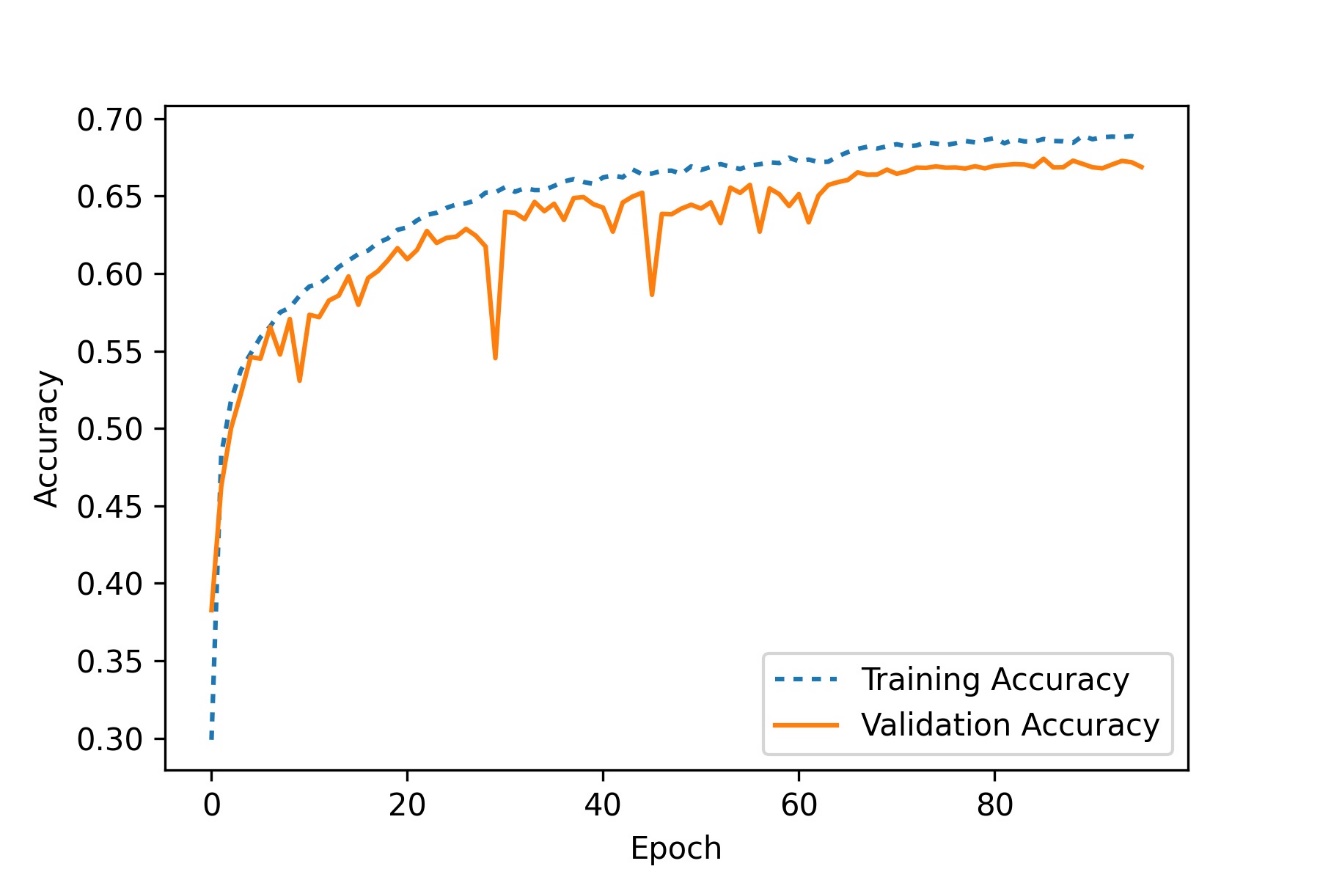


Figure 14S: Validation accuracy vs training accuracy (PUResNet)


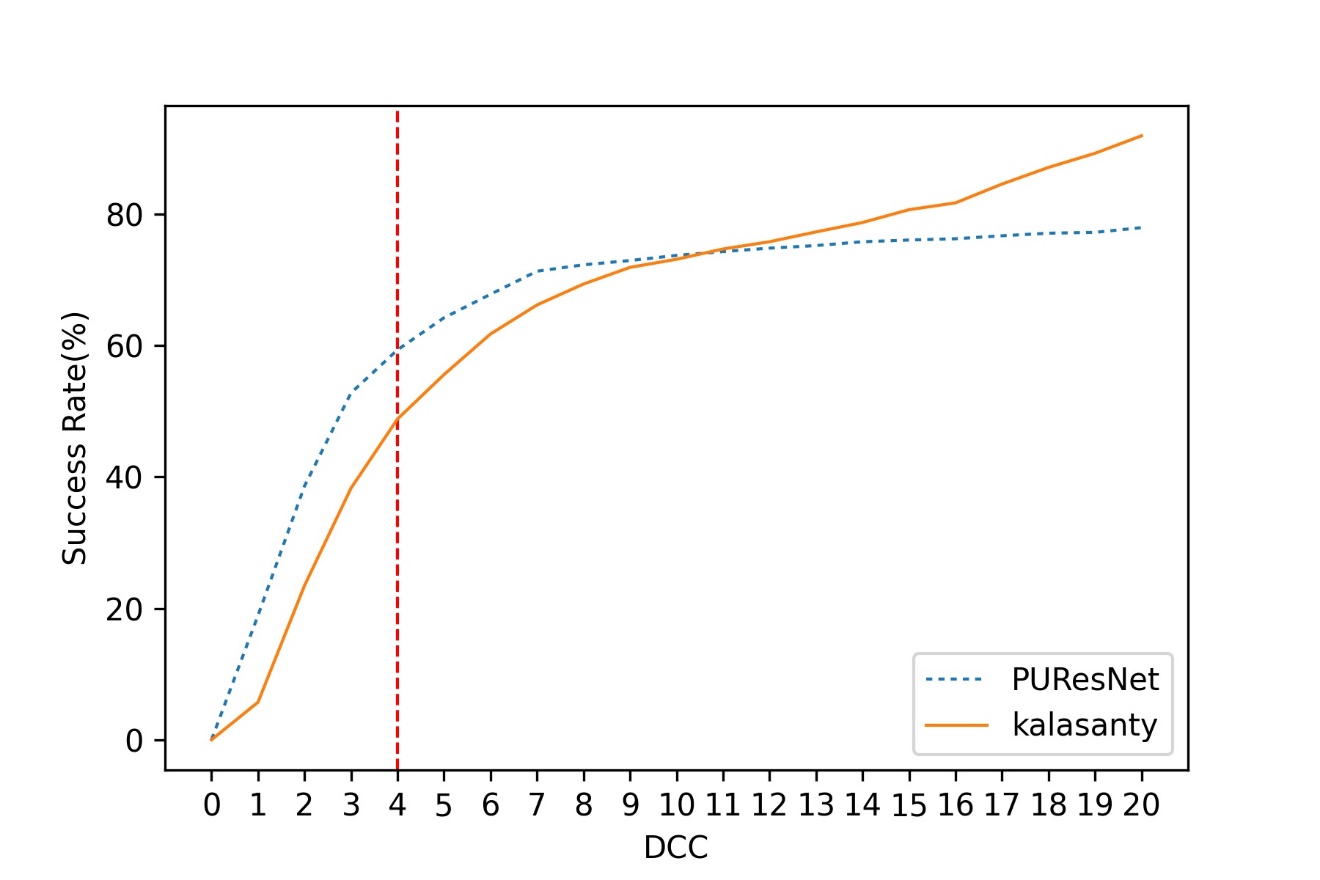


Figure 15S: Success Rate plot for different DCC values (Kalasanty Vs PUResNet)


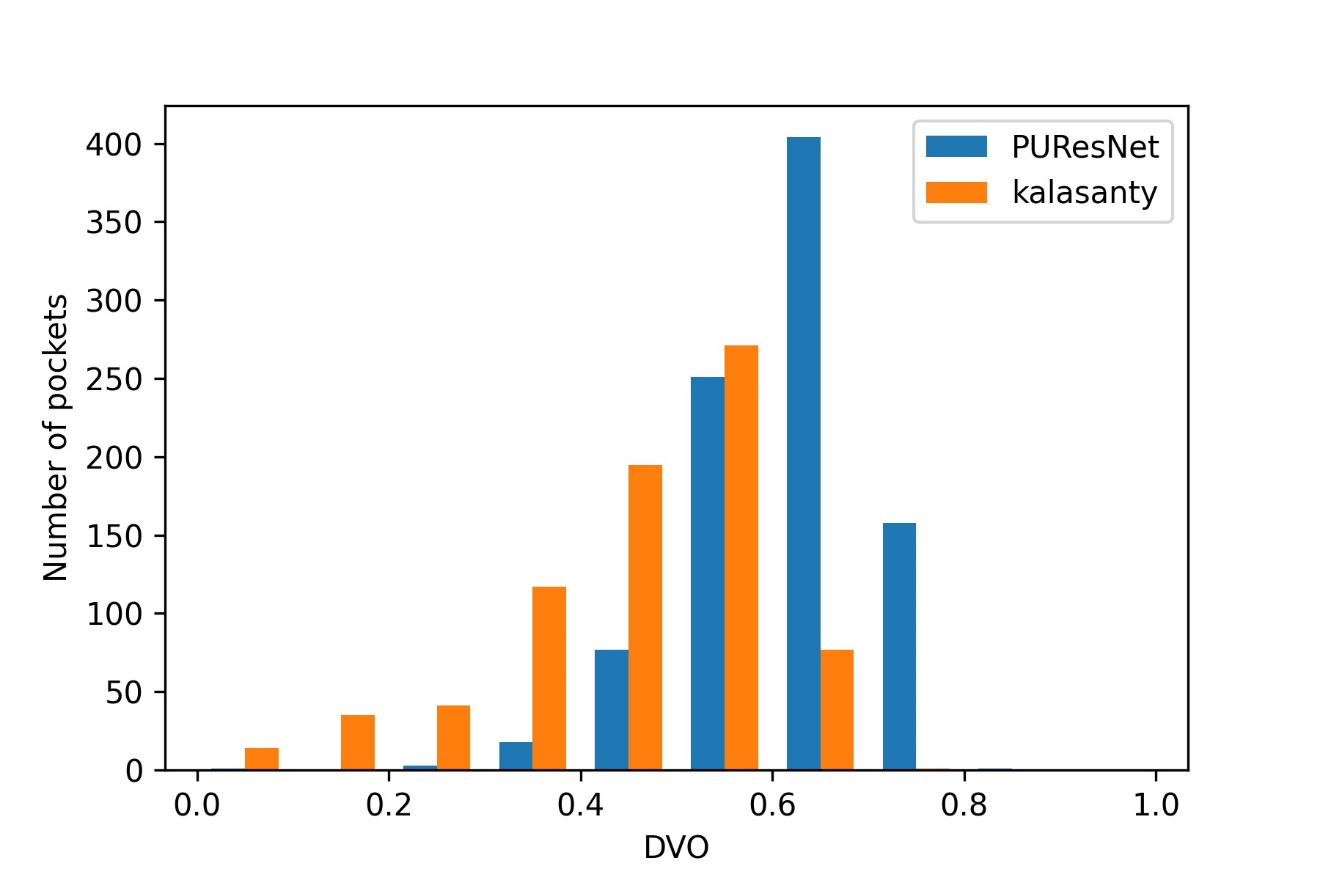


Figure 16S: Histogram of DVO values for protein structure having DCC ≤ 4Å (Kalasanty Vs PUResNet)
