# Supplementary material for: PUResNet: prediction of protein-ligand binding sites using deep residual neural network
Source: J Cheminform. 2021 Sep 8;13:65. doi: 10.1186/s13321-021-00547-7 (PMC8424938; doi:10.1186/s13321-021-00547-7)
Supplement: Supplementary file 4 — Additional file 4. Miscellaneous results. [file 13321_2021_547_MOESM4_ESM.docx]

PUResNet: Predicting protein-ligand binding sites using deep convolutional neural network.

Jeevan Kandel^1^, Hilal Tayara^2*^, and Kil To Chong^4*^

**Additional File 4**

Contents

[Dice Loss vs Binary cross-entropy loss 3](#_Toc80888924)

[Fold 1 3](#_Toc80888925)

[Fold 2 4](#_Toc80888926)

[Fold 3 5](#_Toc80888927)

[Fold 4 6](#_Toc80888928)

[PUResNet without skip connection 7](#_Toc80888929)

[PUResNet with skip connection 8](#_Toc80888930)

[Comparison of PUResNet trained on filtered and non-filtered dataset. 9](#_Toc80888931)

[Coach420 9](#_Toc80888932)

[BU48 10](#_Toc80888933)

# List of Figures

[Figure 1S: Graph showing accuracy comparison between PUResNet trained with binary crossentropy and dice loss. 3](#_Toc80888944)

[Figure 2S: Graph showing loss comparison between PUResNet trained with binary crossentropy and dice loss. 3](#_Toc80888945)

[Figure 3S: Graph showing accuracy comparison between PUResNet trained with binary crossentropy and dice loss. 4](#_Toc80888946)

[Figure 4S: Graph showing loss comparison between PUResNet trained with binary crossentropy and dice loss. 4](#_Toc80888947)

[Figure 5S: Graph showing accuracy comparison between PUResNet trained with binary crossentropy and dice loss. 5](#_Toc80888948)

[Figure 6S: Graph showing loss comparison between PUResNet trained with binary crossentropy and dice loss. 5](#_Toc80888949)

[Figure 7S: Graph showing accuracy comparison between PUResNet trained with binary crossentropy and dice loss. 6](#_Toc80888950)

[Figure 8S: Graph showing loss comparison between PUResNet trained with binary crossentropy and dice loss. 6](#_Toc80888951)

[Figure 9S: Graphs showing loss and accuracy of PUResNet trained on Fold 1 without skip connection. 7](#_Toc80888952)

[Figure 10S: Graph showing gradient norm of layer res6a_branch2b (59^th^ layer), layer res8b_branch2b (105^th^ layer) and last layer (168^th^ layer) during training. 7](#_Toc80888953)

[Figure 11S: Graphs showing loss and accuracy of PUResNet trained on Fold 1 with skip connection. 8](#_Toc80888954)

[Figure 12S: Graph showing gradient norm of layer res6a_branch2b (71^th^ layer), layer res8b_branch2b (132^nd^ layer) and last layer (252^nd^ layer) during training. 8](#_Toc80888955)

[Figure 13S: Success Rate plot for different DCC values 9](#_Toc80888956)

[Figure 14S: Histogram of DVO values for protein structure having DCC ≤ 4Å 9](#_Toc80888957)

[Figure 15S: Histogram of PLI values for protein structure having DCC ≤ 4Å 10](#_Toc80888958)

[Figure 16S: Success Rate plot for different DCC values 10](#_Toc80888959)

[Figure 17S: Histogram of DVO values for protein structure having DCC ≤ 4Å 11](#_Toc80888960)

[Figure 18S: Histogram of PLI values for protein structure having DCC ≤ 4Å 11](#_Toc80888961)

# Dice Loss vs Binary cross-entropy loss

## Fold 1


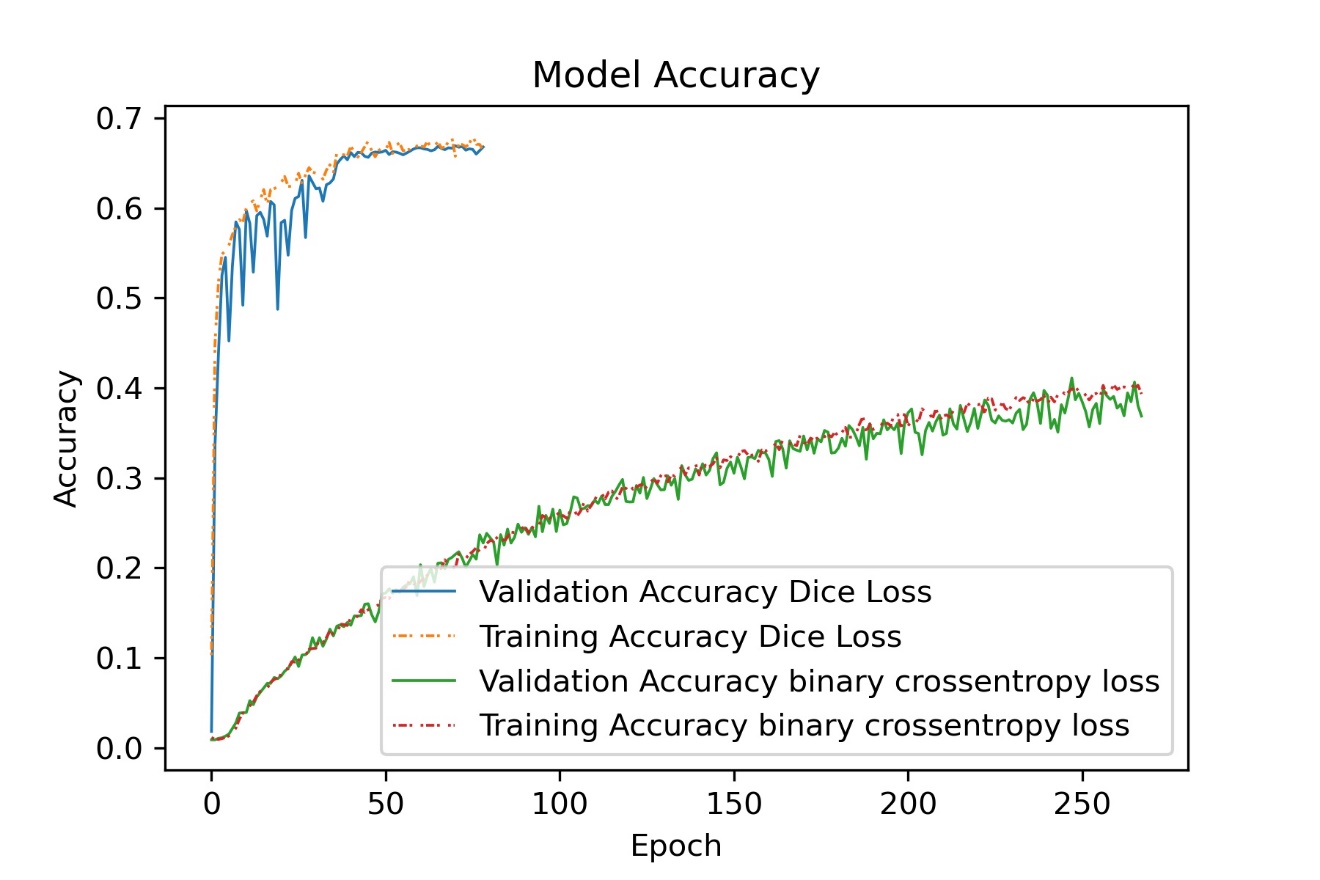


Figure 1S: Graph showing accuracy comparison between PUResNet trained with binary crossentropy and dice loss.


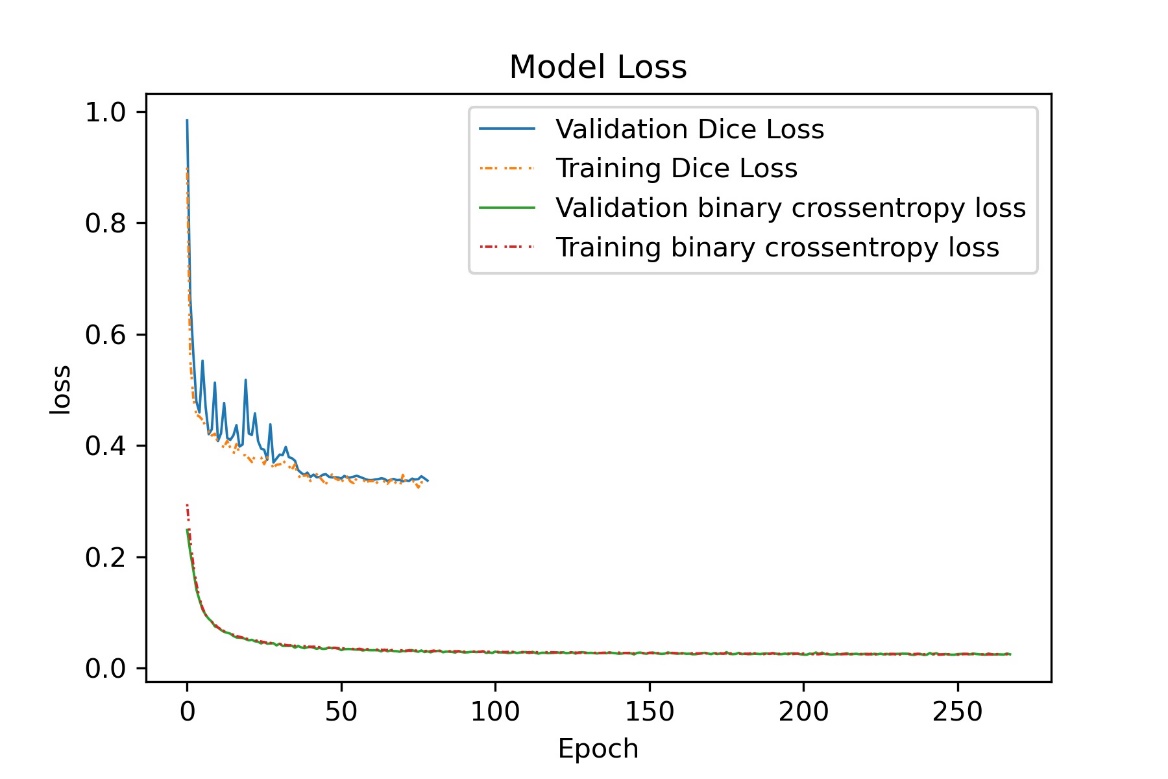


Figure 2S: Graph showing loss comparison between PUResNet trained with binary crossentropy and dice loss.

## Fold 2


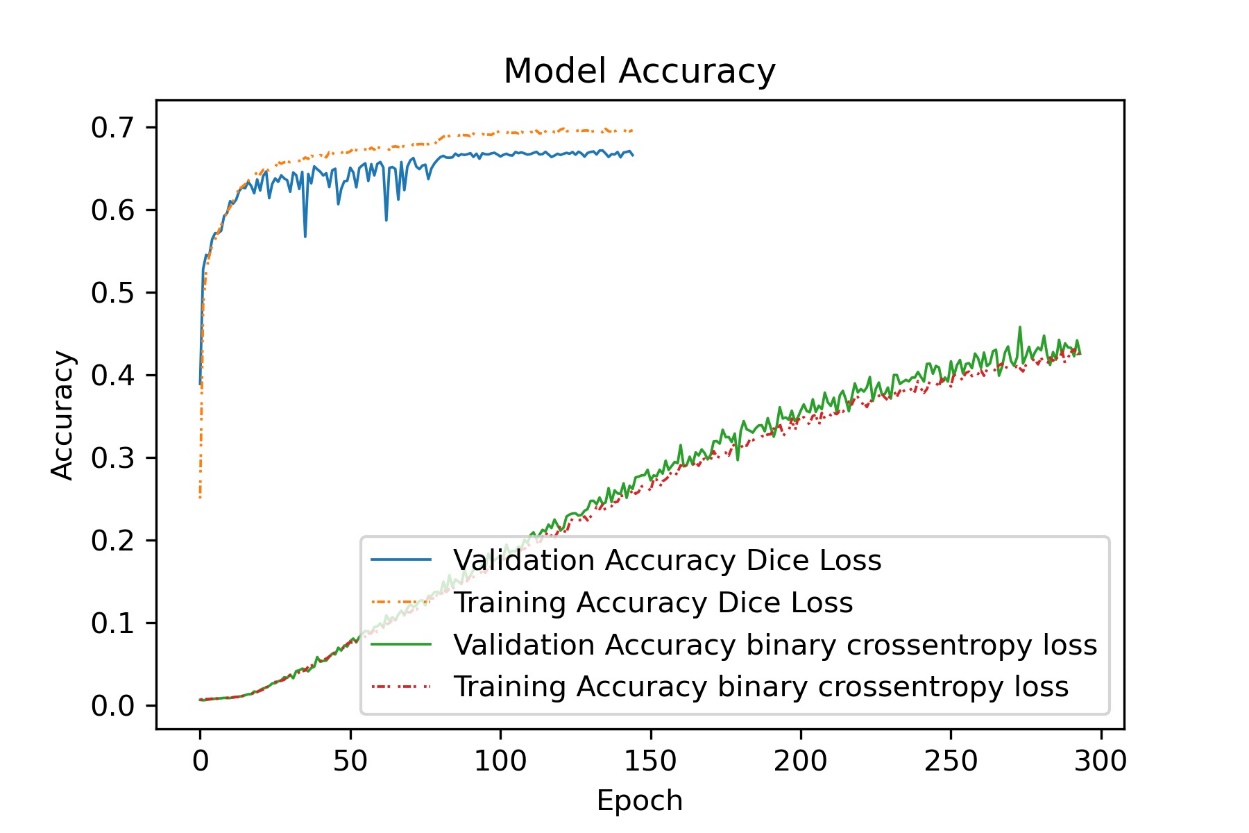


Figure 3S: Graph showing accuracy comparison between PUResNet trained with binary crossentropy and dice loss.


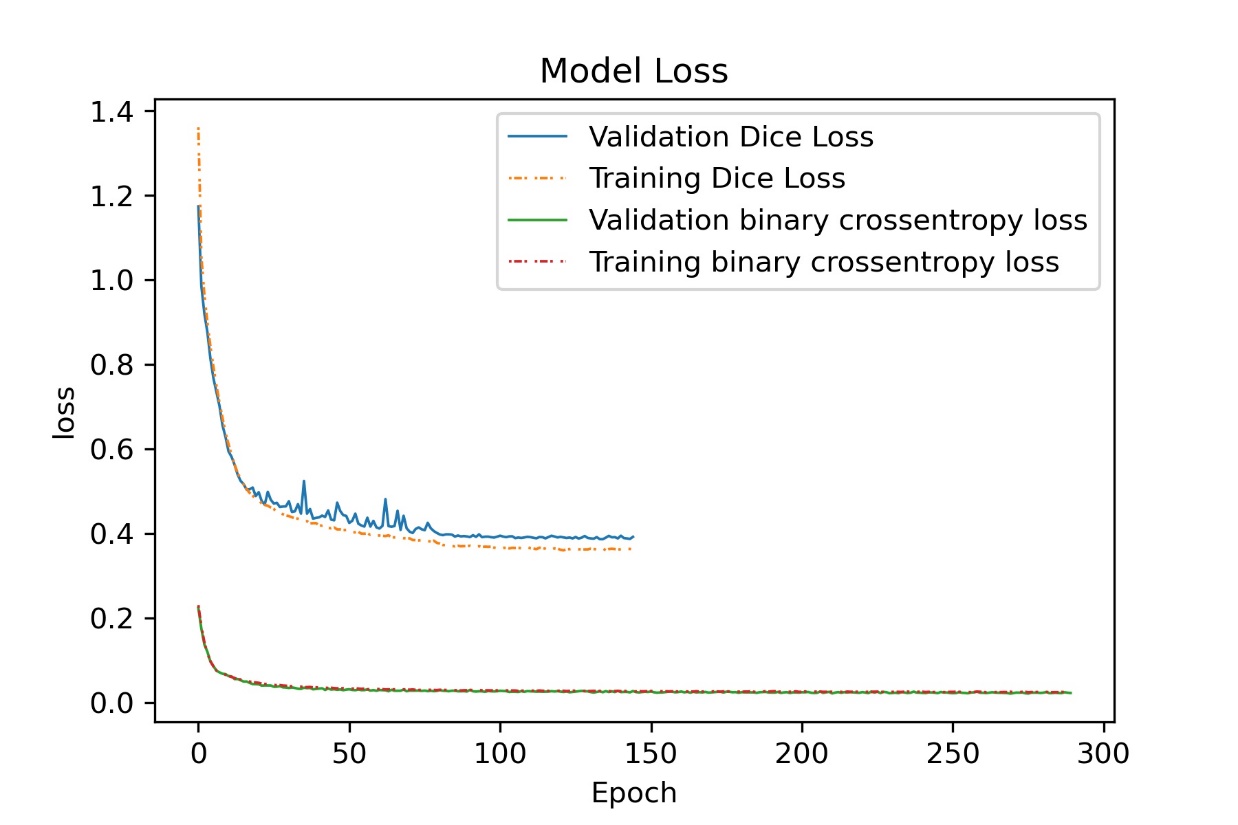


Figure 4S: Graph showing loss comparison between PUResNet trained with binary crossentropy and dice loss.

## Fold 3


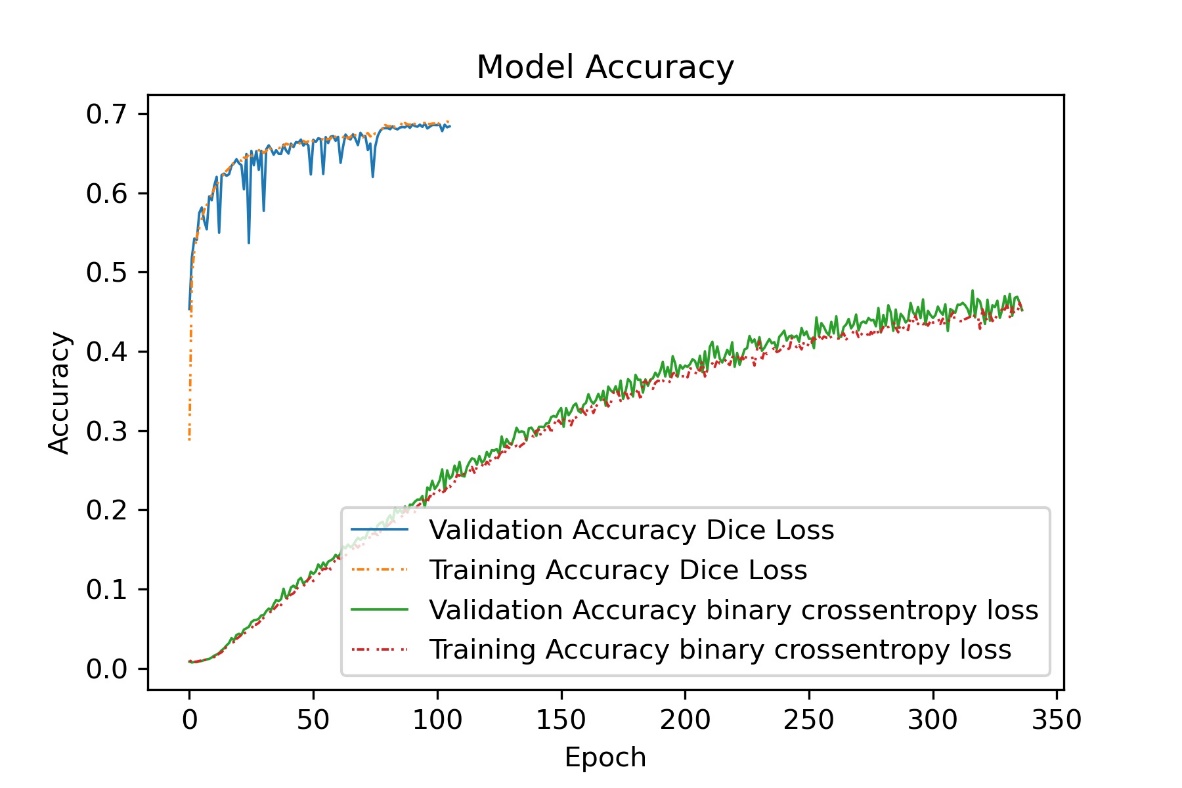


Figure 5S: Graph showing accuracy comparison between PUResNet trained with binary crossentropy and dice loss.


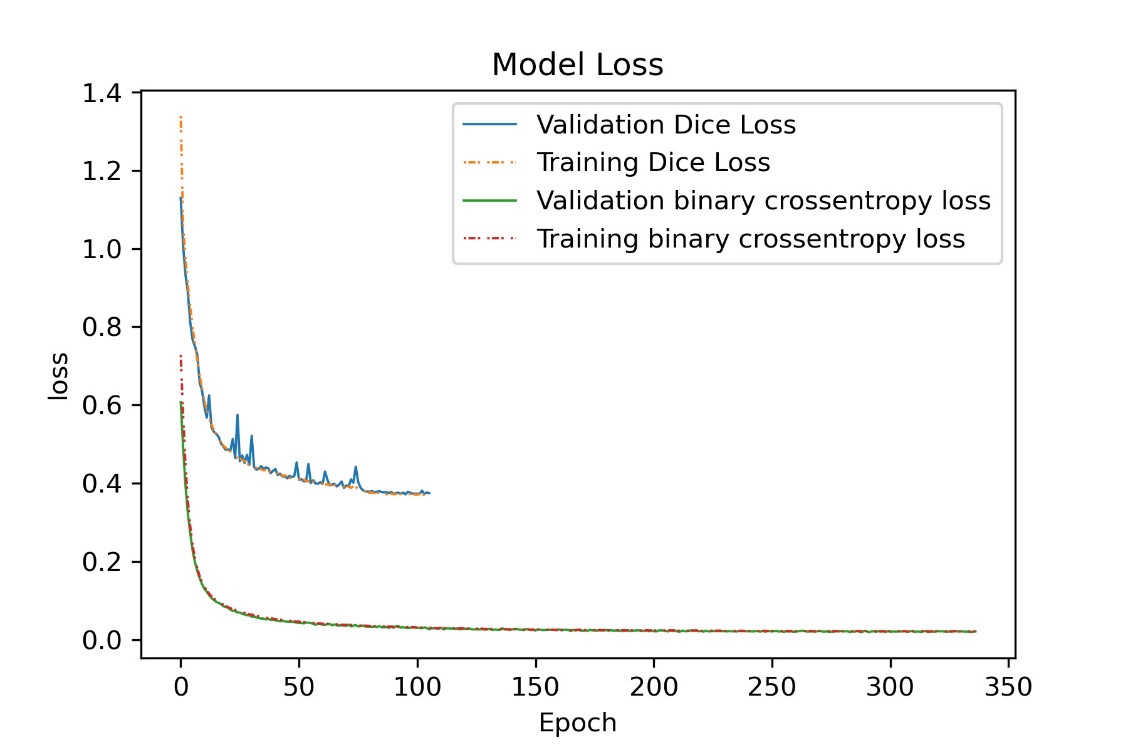


Figure 6S: Graph showing loss comparison between PUResNet trained with binary crossentropy and dice loss.

## Fold 4


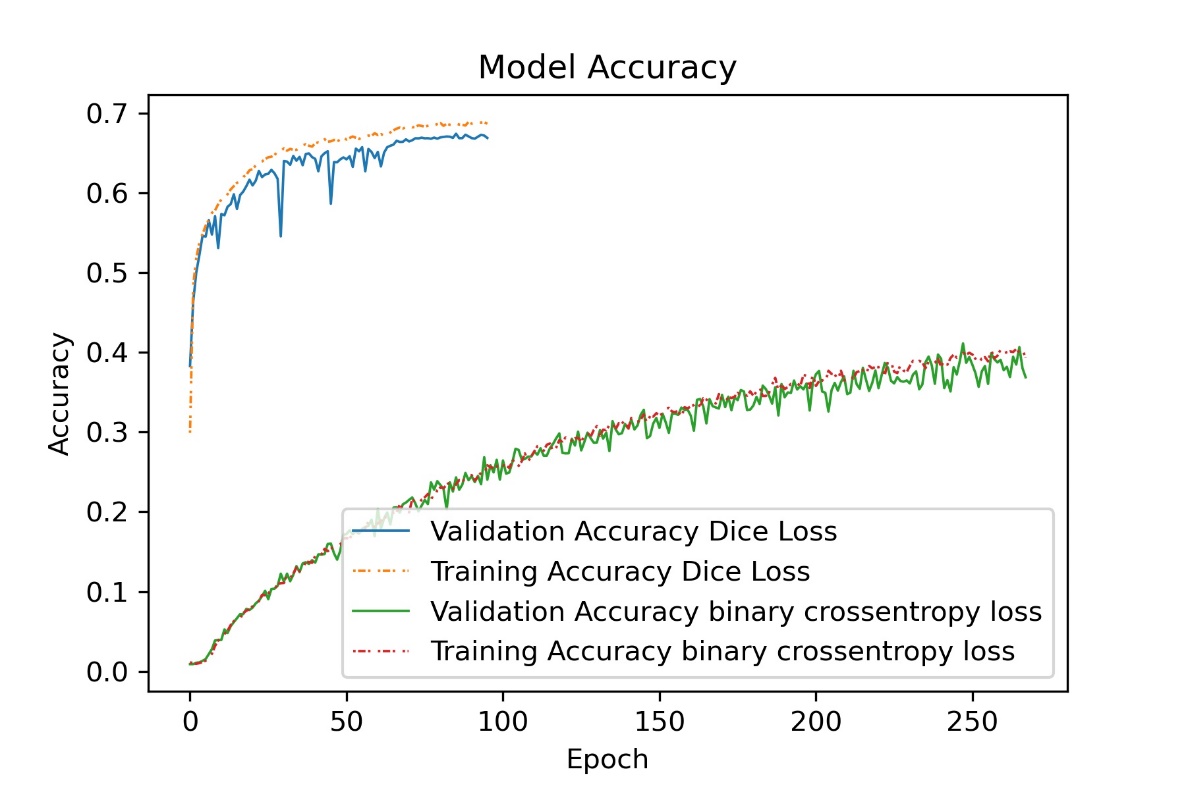


Figure 7S: Graph showing accuracy comparison between PUResNet trained with binary crossentropy and dice loss.


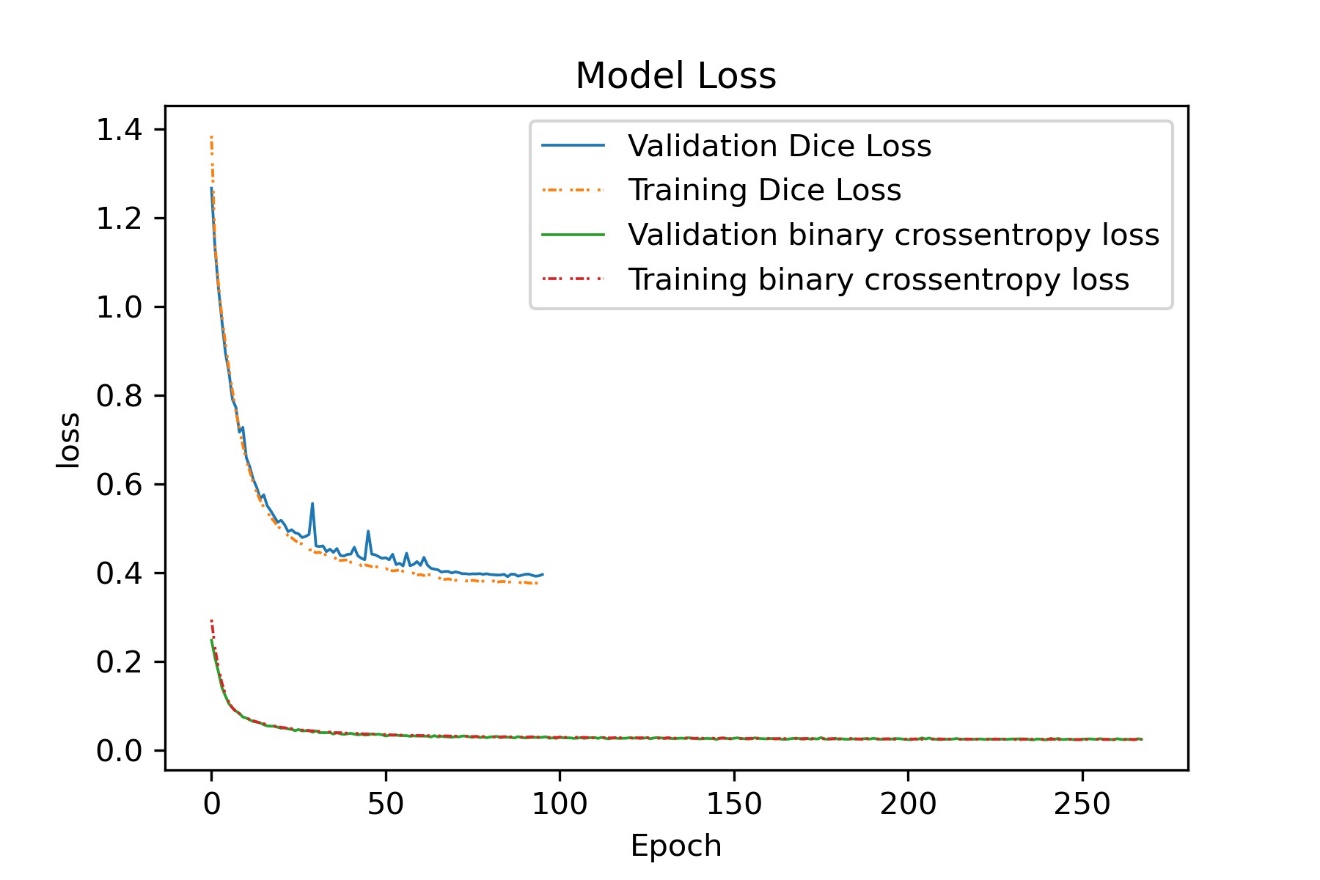


Figure 8S: Graph showing loss comparison between PUResNet trained with binary crossentropy and dice loss.

# PUResNet without skip connection


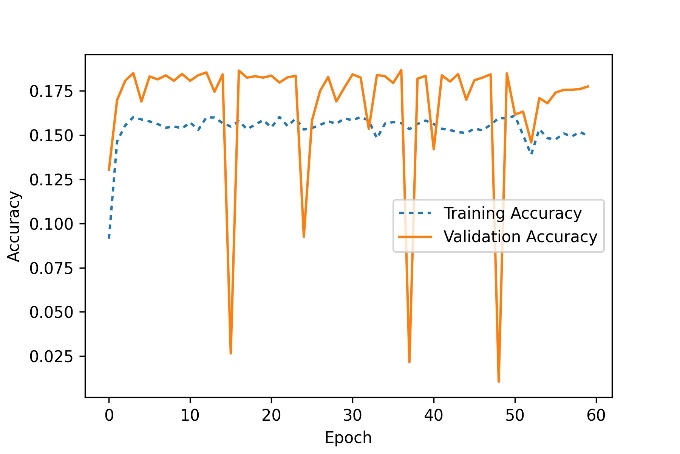

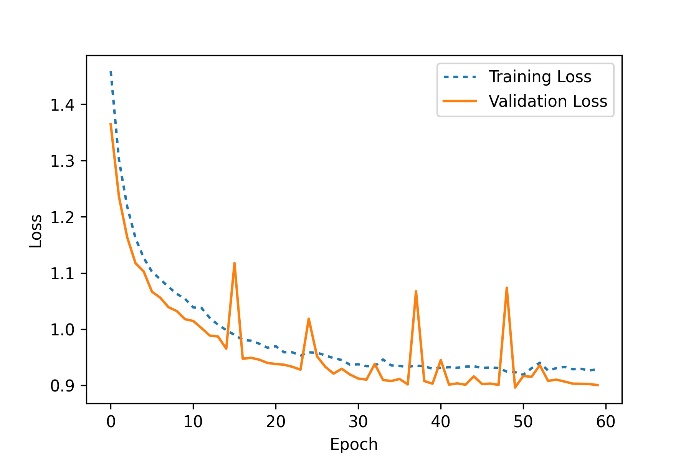


Figure 9S: Graphs showing loss and accuracy of PUResNet trained on Fold 1 without skip connection.


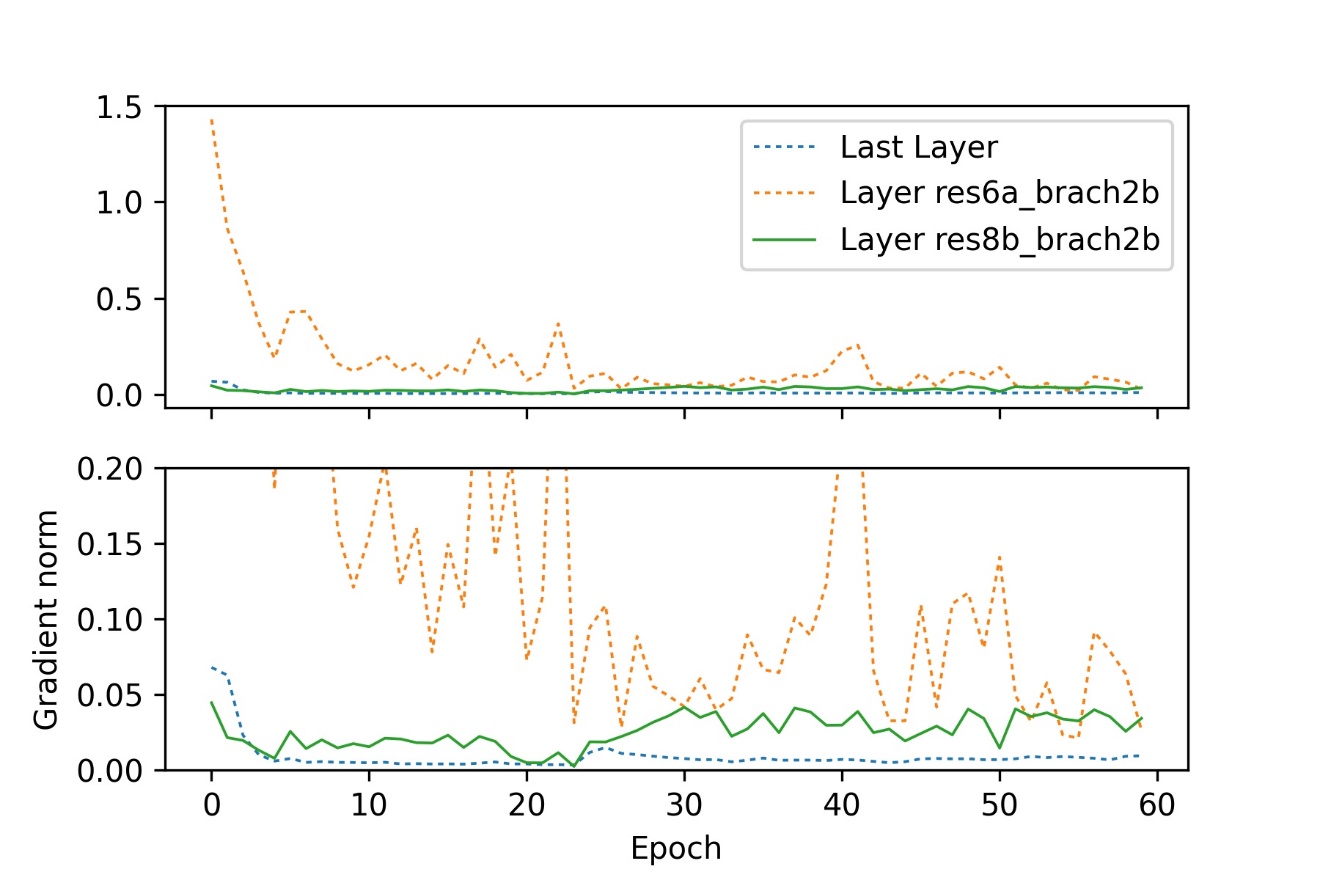


Figure 10S: Graph showing gradient norm of layer res6a_branch2b (59^th^ layer), layer res8b_branch2b (105^th^ layer) and last layer (168^th­^ layer) during training.

# PUResNet with skip connection


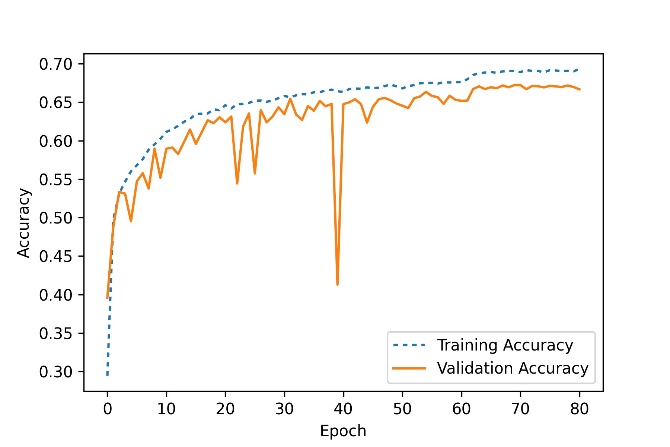

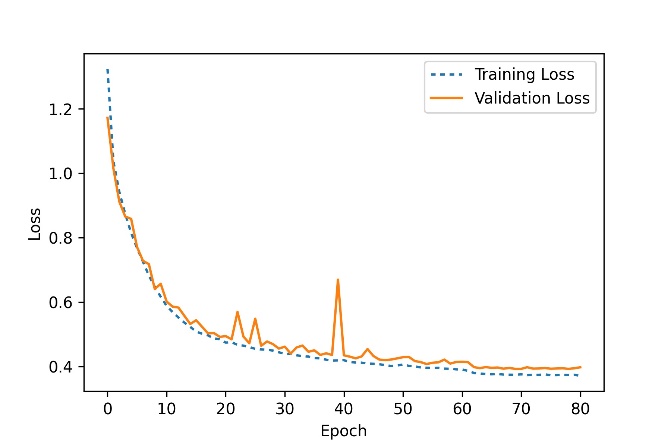


Figure 11S: Graphs showing loss and accuracy of PUResNet trained on Fold 1 with skip connection.


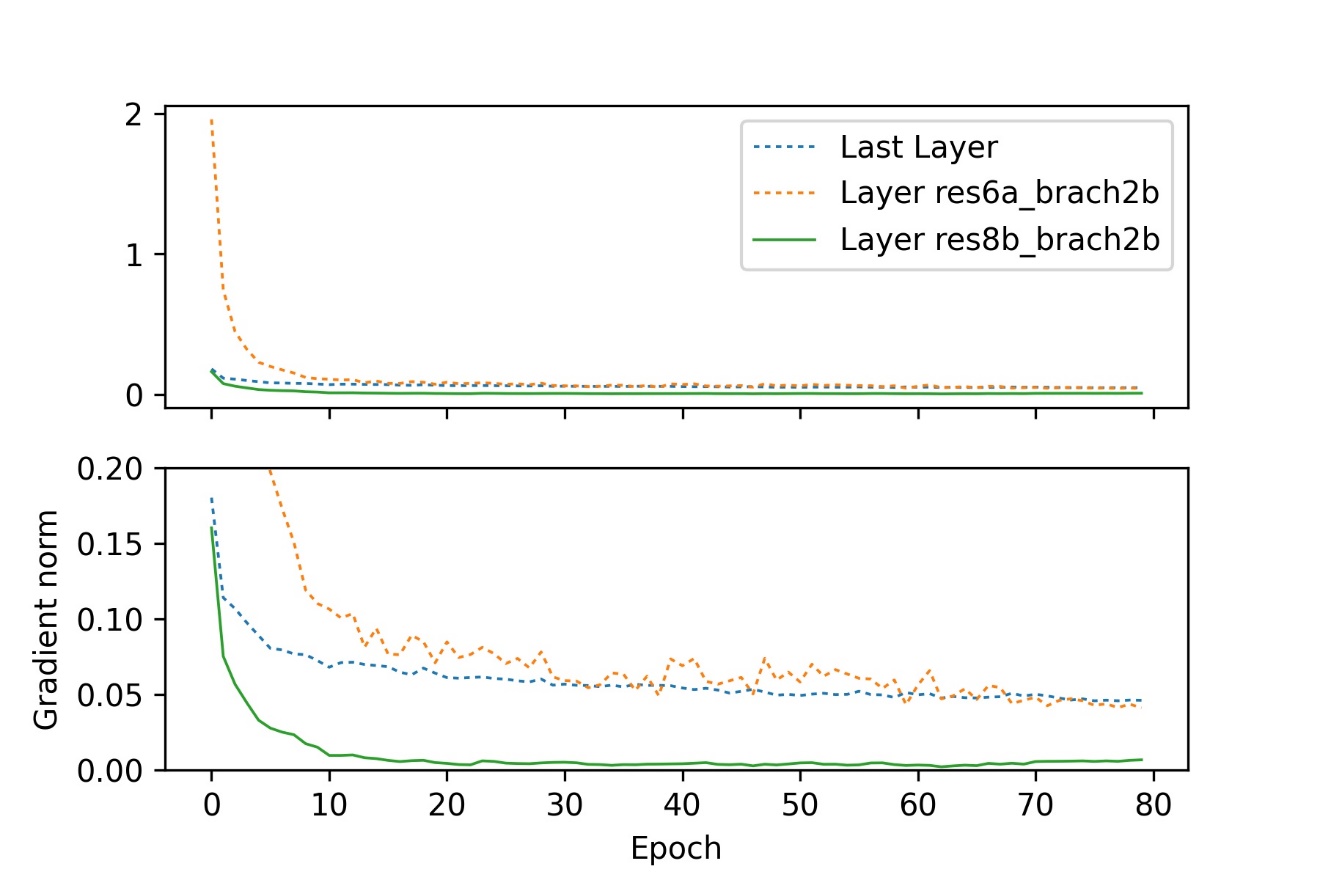


Figure 12S: Graph showing gradient norm of layer res6a_branch2b (71^st^ layer), layer res8b_branch2b (132^nd^ layer) and last layer (252^nd­^ layer) during training.

# Comparison of PUResNet trained on filtered and non-filtered dataset.

## Coach420


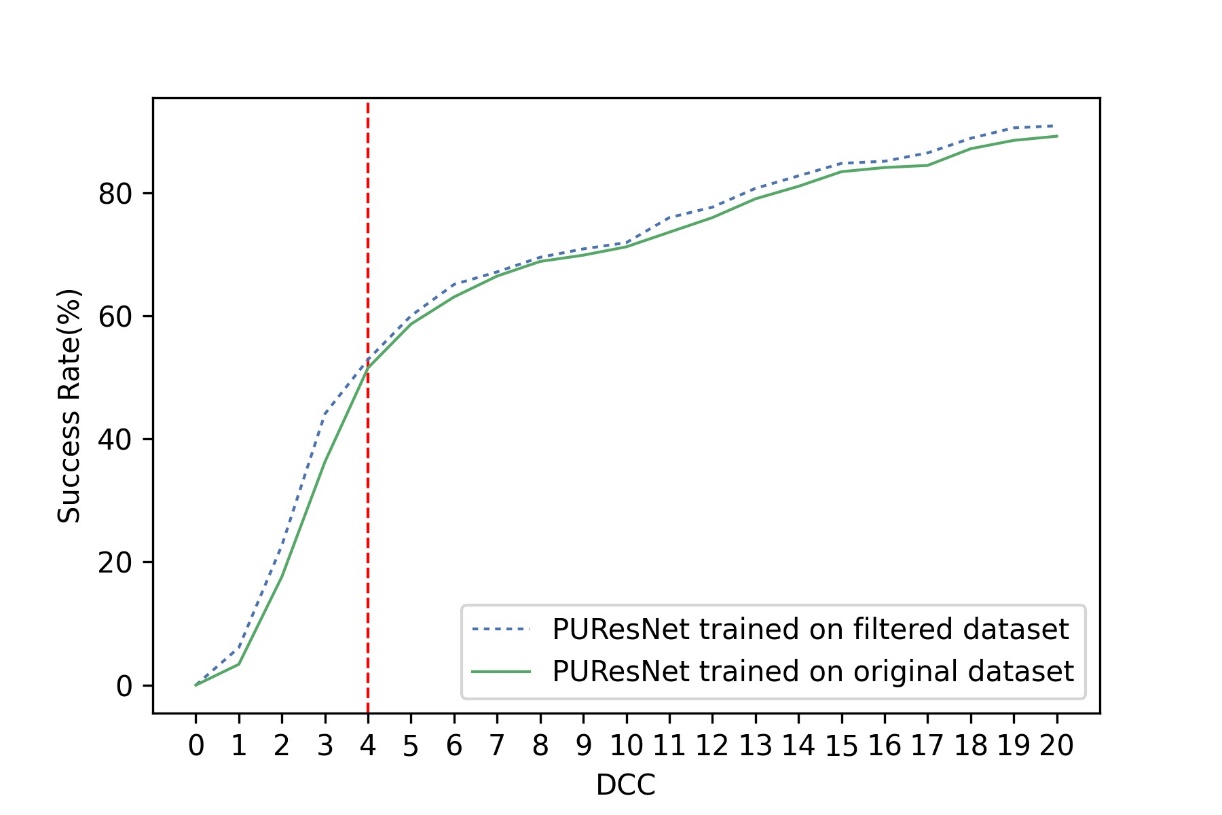


Figure 13S: Success Rate plot for different DCC values


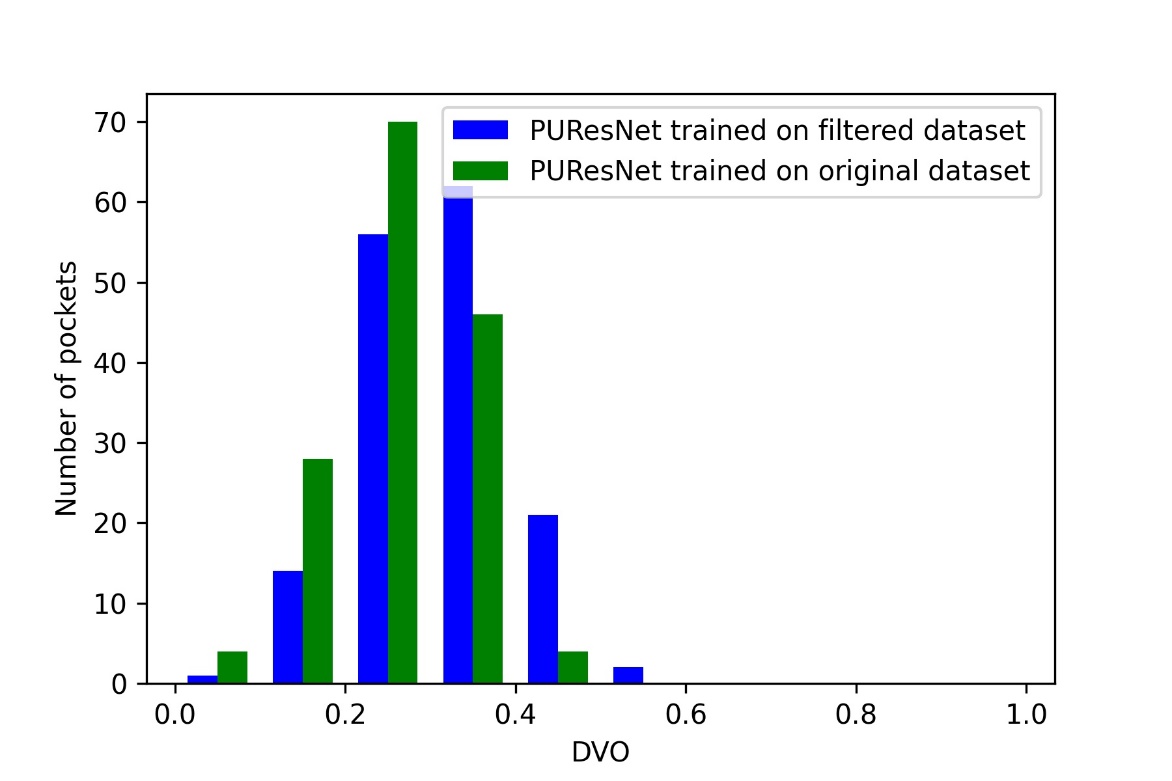


Figure 14S: Histogram of DVO values for protein structure having DCC ≤ 4Å


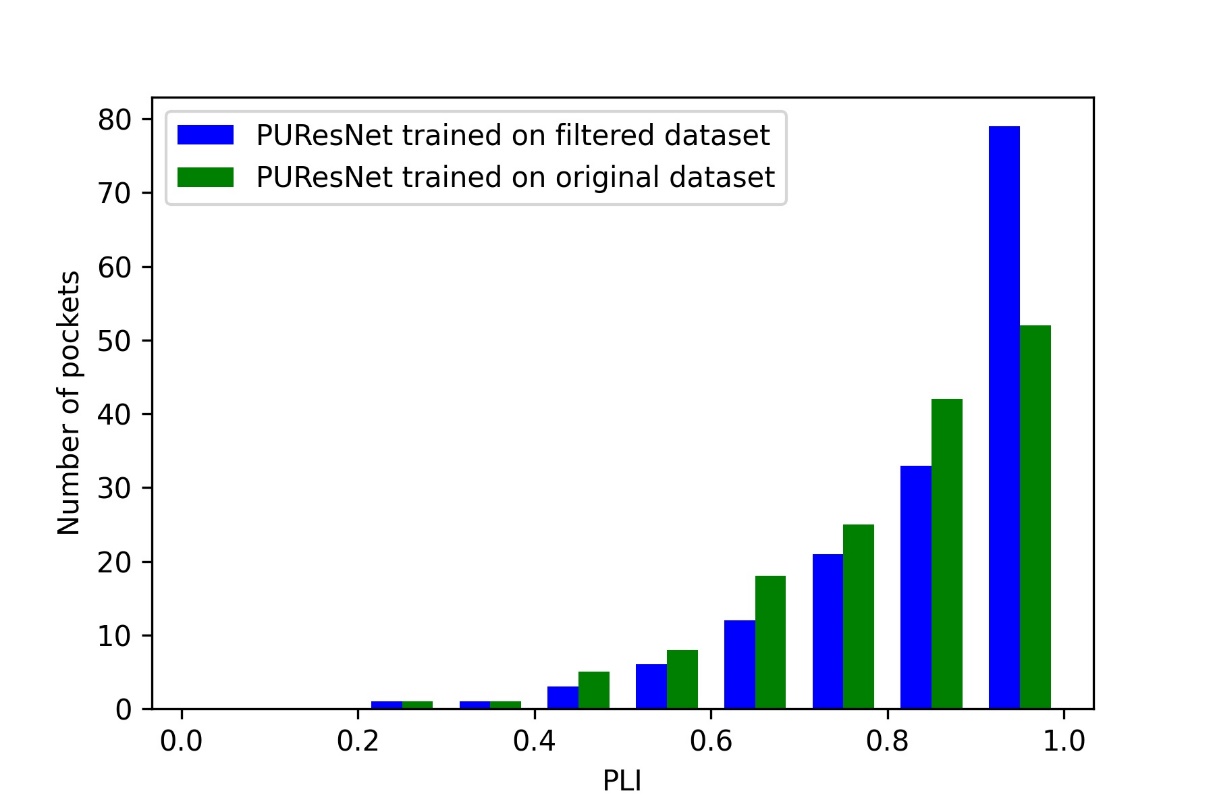


Figure 15S: Histogram of PLI values for protein structure having DCC ≤ 4Å

## BU48


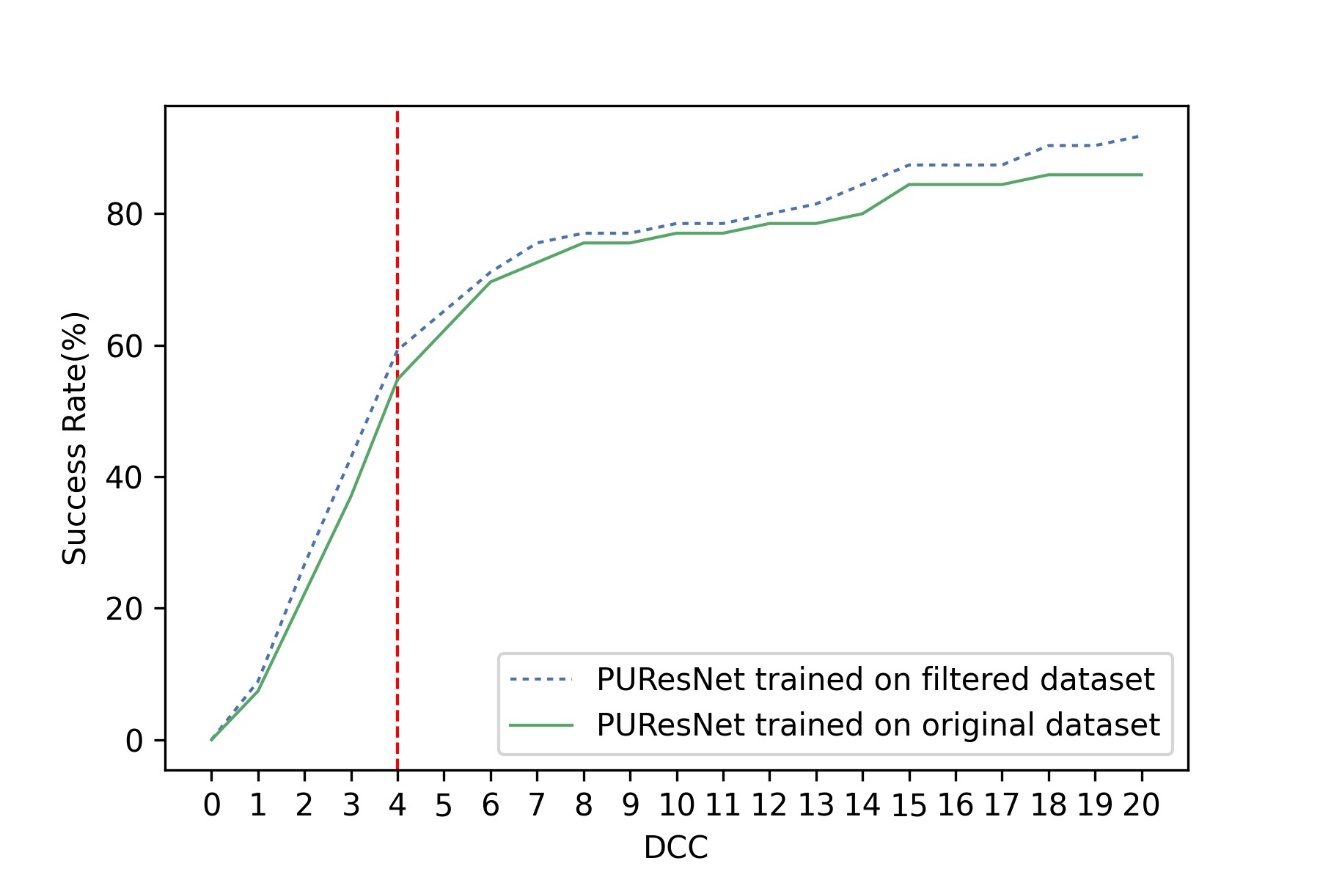


Figure 16S: Success Rate plot for different DCC values


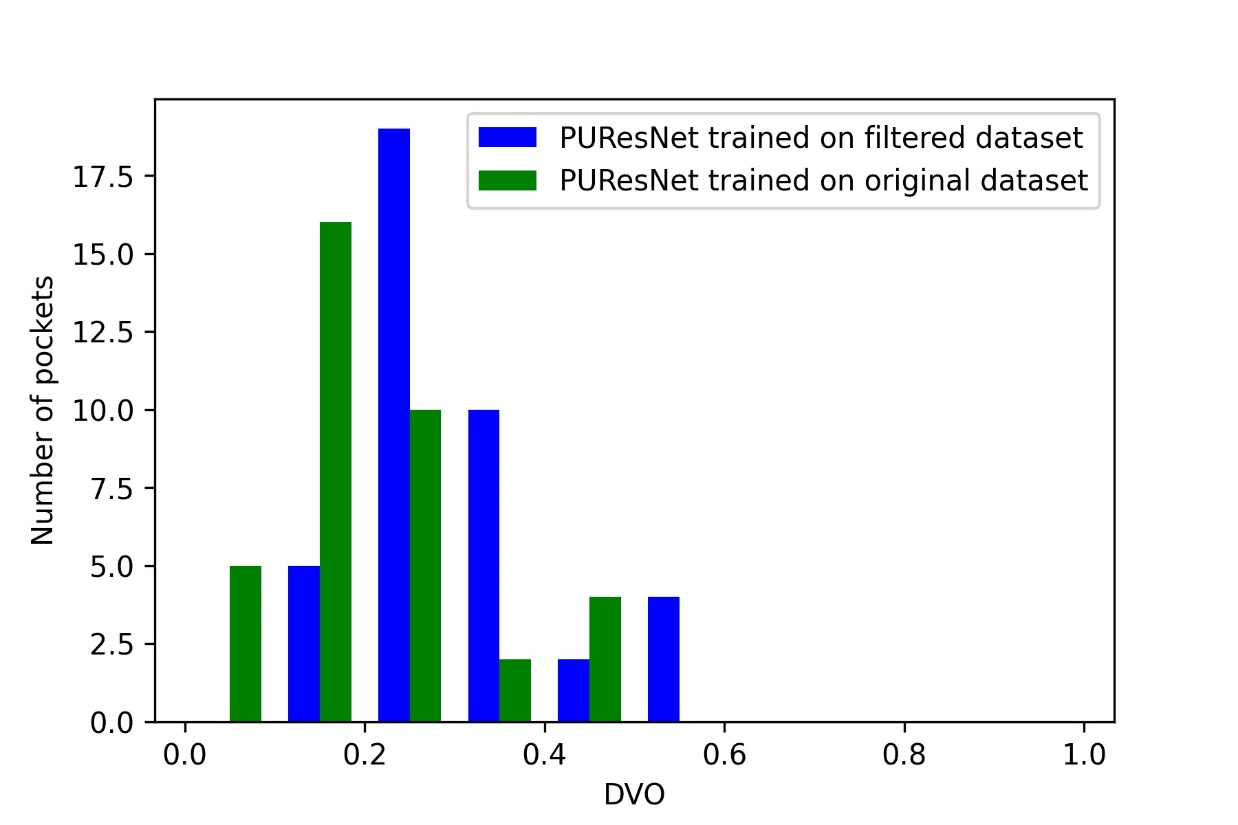


Figure 17S: Histogram of DVO values for protein structure having DCC ≤ 4Å


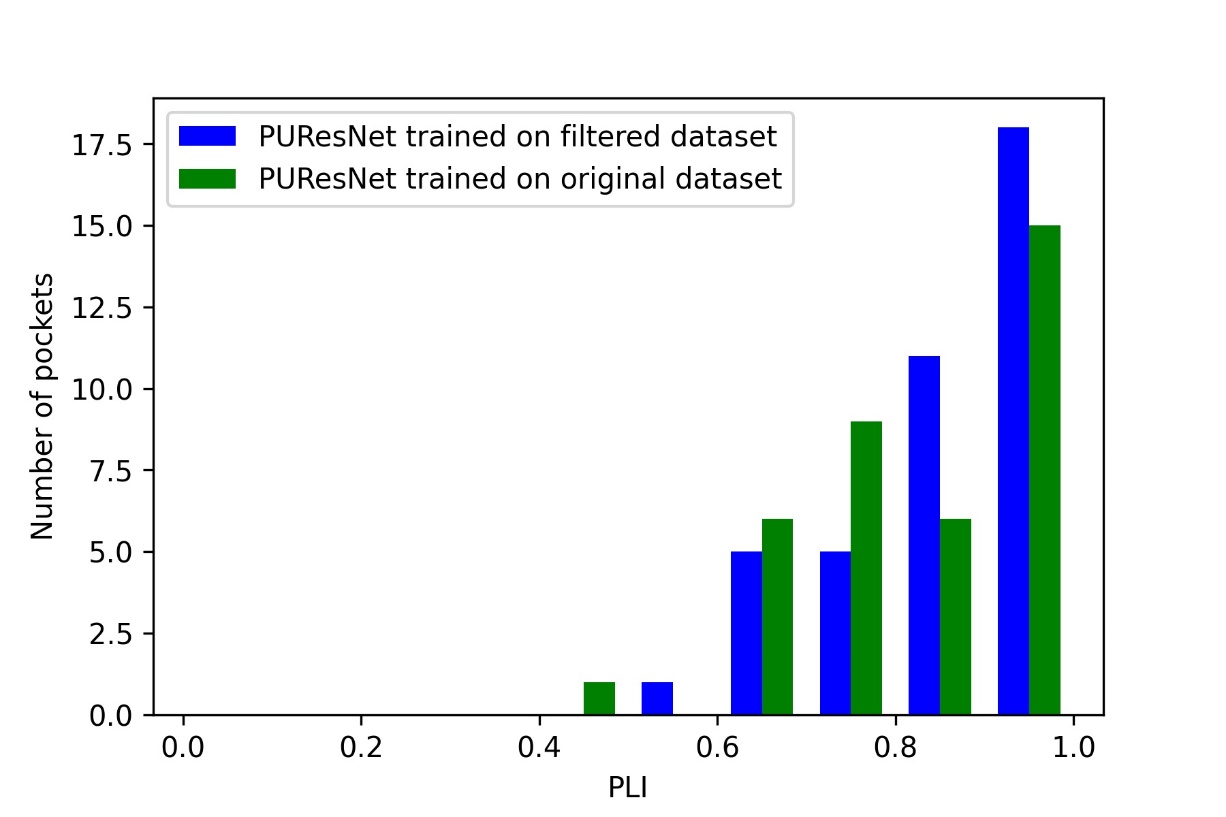


Figure 18S: Histogram of PLI values for protein structure having DCC ≤ 4Å
